# Thiophosphonium–Alkyne Cycloaddition Reactions: A Heavy Congener of the Carbonyl–Alkyne Metathesis

**DOI:** 10.1021/acs.inorgchem.1c02076

**Published:** 2021-09-15

**Authors:** Pawel Löwe, Milica Feldt, Maike B. Röthel, Lukas F. B. Wilm, Fabian Dielmann

**Affiliations:** † Institut für Anorganische und Analytische Chemie, Westfälische Wilhelms-Universität Münster, Corrensstrasse 28-30, 48149 Münster, Germany; ‡ Theoretische Organische Chemie, Organisch-Chemisches Institut and Center for Multiscale Theory and Computation, Westfälische Wilhelms-Universität Münster, Corrensstraße 36, 48149 Münster, Germany; § Department of General, Inorganic and Theoretical Chemistry, Leopold-Franzens-Universität Innsbruck, Innrain 80-82, 6020 Innsbruck, Austria

## Abstract

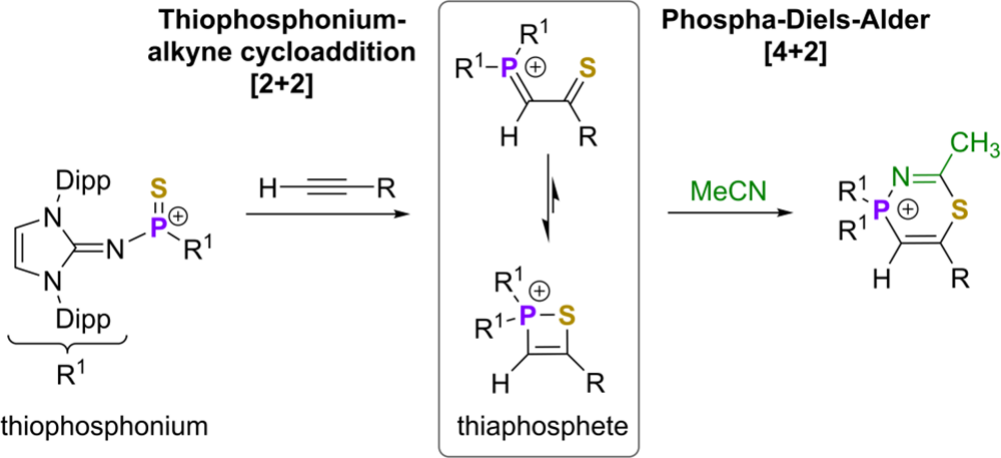

While the metathesis reaction between alkynes and thiocarbonyl compounds has been thoroughly studied, the reactivity of alkynes with isoelectronic main group R_2_E=S compounds is rarely reported and unknown for [R_2_P=S]^+^ analogues. We show that thiophosphonium ions, which are the isoelectronic phosphorus congeners to thiocarbonyl compounds, undergo [2 + 2]-cycloaddition reactions with different alkynes to generate 1,2-thiaphosphete ions. The four-membered ring species are in an equilibrium state with the corresponding P=C–C=S heterodiene structure and thus undergo hetero-Diels–Alder reactions with acetonitrile. Heteroatom and substituent effects on the energy profile of the 1,2-thiaphosphete formation were elucidated by means of quantum chemical methods.

## Abstract 2

We show that thiophosphonium ions, which are the isoelectronic phosphorus congeners to thiocarbonyl compounds, undergo [2 + 2]-cycloaddition reactions with different alkynes to generate 1,2-thiaphosphete ions. The four-membered ring species are in an equilibrium state with the corresponding P=C−C=S heterodiene structure and thus undergo hetero-Diels−Alder reactions with acetonitrile. Heteroatom and substituent effects on the energy profile of the 1,2-thiaphosphete formation were elucidated by means of quantum chemical methods.

## Introduction

**Scheme 1:**
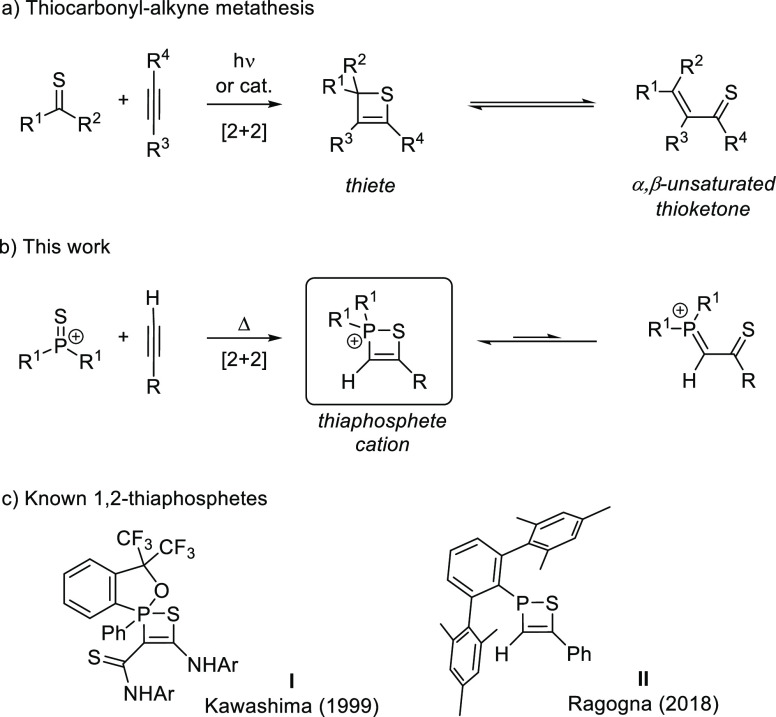
(a) Reaction of Thiocarbonyls with Alkynes, (b) Reaction of Thiophosphonium Ions with Alkynes to Give 1,2-Thiaphosphete Cations Presented in This Work, and (c) Neutral P^V^ 1,2-Thiaphosphete by Kawashima (**I**) and P^III^ 1,2-Thiaphosphete by Ragogna (**II**).

Heavy analogues of carbonyl compounds are generally highly reactive and prone to spontaneous oligomerization owing to the energetic preference of heavy p-block elements in forming σ bonds instead of (p–p)π bonds.^1−4^ In this respect, the thiocarbonyl group (C=S) is an exception, but it reacts, due to its rather weak C=S bond and the aptitude of sulfur to stabilize an adjacent charge or radical center, more easily in nucleophilic reactions and sigmatropic rearrangements than carbonyls.^5^ Both carbonyls and thiocarbonyls undergo (thio)carbonyl–alkyne metathesis reactions, involving the [2 + 2]-cycloaddition reaction of a (thio)carbonyl with an alkyne. These reactions have been extensively utilized in synthetic chemistry.^6^ The carbonyl–alkyne metathesis proceeds via a four-membered oxete intermediate, which is usually directly transformed into the α,β-unsaturated ketone,^7−12^ unless it is stabilized by strongly electron-withdrawing groups.^13−15^ Due to the lower tendency of sulfur to form double bonds, thietes are more stable than oxetes,^16−21^ and a dynamic equilibrium between the “closed” thiete and “open” α,β-unsaturated thioketone form was observed with thioether substituents.^22,23^ Given these differences between oxetes and thietes, we became curious to explore how the introduction of another heavy main group element would affect the stability of the four-membered ring species. Although numerous examples for heavy main group carbonyls R_2_E=O and thiocarbonyls R_2_E=S have been synthesized,^24−34^ the reactivity with alkynes is little developed. Stannanethiones undergo [2 + 2]-cycloaddition reactions with the particularly electron-poor alkyne dimethyl acetylenedicarboxylate in a stepwise mechanism to give 1,2-thiastannete.^35,36^ The reaction mode of stannaneselone and stannanetellone was found to be similar, but ring-opening and formation of the corresponding stannabutadiene was not observed.^35,37^ Similarly, in transition metal chemistry, the elusive zirconasulfide [Cp*_2_Zr=S] (Cp* = pentamethylcyclopentadienyl) was trapped via [2 + 2]-cycloadditions with alkynes yielding 1,2-thiazirconabutenes.^38,39^ Recently, we explored the cycloaddition reaction between oxophosphonium cations and alkynes and showed that by using strong π-donor substituents instead of alkyl groups at the phosphorus atom, the “closed” oxaphosphete and the “open” 1-phospha-4-oxa-butadiene get closer in energy.^40^ Enabled by our recent success in isolating the first Lewis-base-free thiophosphonium ion [R_2_P=S]^+^,^41^ we herein report on [2 + 2]-cycloaddition reactions of thiophosphonium salts with alkynes, yielding 1,2-thiaphosphete cations (Scheme 1b). The first neutral P^V^ 1,2-thiaphosphete was synthesized by Kawashima and co-workers containing a P-center stabilized by the Martin ligand (Scheme 1, **I**).^42^ More recently, Ragogna and co-workers prepared the neutral P^III^ 1,2-thiaphosphete **II** via transfer of a phosphinidene sulfide intermediate to an alkyne.^43^

## Results and Discussion

**Scheme 2:**
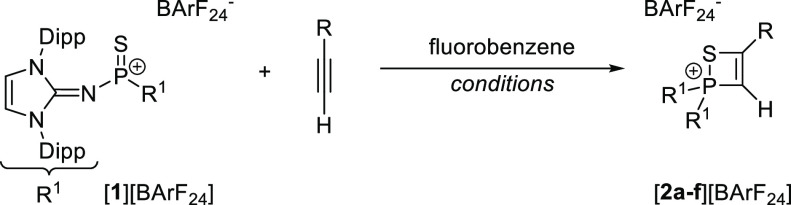
Synthesis of Thiaphosphete Salts [**2a**–**f**][BArF_24_] R = aryl, ethoxy, or methyl(*p*-toluenesulfonyl)amide (see Table 1). Dipp = 2,6-diisopropylphenyl.

We began our studies by reacting thiophosphonium salts [**1**][X] (X = tetrakis[3,5-bis(trifluoromethyl)phenyl]borate [BArF_24_]^−^, trifluoromethanesulfonate [OTf]^−^) with alkynes. Heating a fluorobenzene solution containing [**1**][BArF_24_] and phenylacetylene to 120 °C gave the [2 + 2]-cycloaddition product [**2a**][BArF_24_] as a beige, moisture-sensitive solid in quantitative yield (Scheme 2). The thiaphosphete salt [**2a**][BArF_24_] shows a characteristic doublet at −36.1 ppm (^2^*J*_PH_ = 19 Hz) in the ^31^P NMR spectrum, which appears at lower frequency than the ^31^P NMR resonance of the thiophosphonium ion [**1**]^+^ (116.6 ppm).^41^ The reaction of the triflate salt [**1**][OTf] with phenylacetylene is less selective (see chapter 1.4 in the SI for details). Therefore, [**1**][BArF_24_] was used in the present study.

The formation of the four-membered heterocycle [**2a**]^+^ is further confirmed by the ^13^C{^1^H} NMR spectrum, revealing a doublet at 120.3 ppm (^1^*J*_PC_ = 106 Hz) for the phosphorus-bound carbon atom and a doublet at 153.4 ppm (^2^*J*_PC_ = 5 Hz) of the adjacent carbon atom, which is deshielded by the sulfur atom. The ^1^H NMR resonance of the thiaphosphete ring proton appears at 3.80 ppm and is significantly shifted to lower frequency compared to that of the parent thiete C_4_H_4_S (6.50 ppm).^44^ The effect can be explained by an enhanced polarization of the C=C bond of the thiaphosphete heterocycle, resulting from the negative hyperconjugation of π-electron density from the carbon atom into low-lying σ* orbitals of the phosphorus atom. The ^31^P NMR resonance of the thiaphosphete salt [**2a**][BArF_24_] appears at lower frequency than that of the analogous oxaphosphete salt (−14.6 ppm).^40^ P^V^ thiaphosphete **I** contains a pentavalent phosphorus atom and exhibits a similar ^31^P NMR chemical shift (−40.7 ppm) to [**2a**]^+^,^42^ whereas the resonance of the P^III^ thiaphosphete **II** appears at 37.5 ppm.^43^

In order to explore possible substituent effects on the [2 + 2]-cycloaddition reaction, acetylene derivatives with electron-donating groups were reacted with thiophosphonium salt [**1**][BArF_24_] (Scheme 2 and Table 1), which gave the thiaphosphete salts [**2b**–**e**][BArF_24_] in excellent yields. The cycloaddition reaction with electron-rich alkynes, e.g., *para*-(dimethylamino)phenylacetylene (entry 3) and ethoxyacetylene (entry 4), is significantly faster than that with phenylacetylene. The electron-poor alkyne 1-ethynyl-3,5-bis(trifluoromethyl)benzene (entry 6) reacted with [**2a**][BArF_24_] very slowly, even with prolonged heating at 180 °C. After 16 h, only 12% conversion was observed. This accelerated cycloaddition reaction between [**2a**][BArF_24_] and electron-rich alkynes can be explained by the high electrophilicity of the thiophosphonium cation and is contrary to the reactivity trend of neutral stannanethiones.^35^ The same regioselectivity was observed for all [2 + 2]-cycloaddition reactions, which agrees with that of the 1,2-thiaphosphete **II**.^43^

**Table 1:** Scope of Terminal Alkynes in [2 + 2]-Cycloaddition Reactions with Thiophosphonium Salt [**1**][BArF_24_]^a^

entry	compd.	R	cond.	yield	δ(^31^P) [^2^*J*_PH_]
1	[**2a**]^+^	Ph–	120 °C, 16 h	99%	–36.1 ppm [19 Hz]
2	[**2b**]^+^	*p*-MeO–C_6_H_4_–	60 °C, 3 h	99%	–35.2 ppm [19 Hz]
3	[**2c**]^+^	*p*-Me_2_N–C_6_H_4_–	21 °C, 2 h	99%	–32.2 ppm [20 Hz]
4	[**2d**]^+^	EtO–	21 °C, 2 h	99%	–35.7 ppm [15 Hz]
5	[**2e**]^+^	TsMeN–	21 °C, 2 h	97%	–36.4 ppm [15 Hz]
6	[**2f**]^+^	3,5-CF_3_–C_6_H_3_–	180 °C, 16 h	12%^b^	–40.6 ppm^c^ [18 Hz]

a The NMR data were obtained from CD_2_Cl_2_ solutions. Ts = *p*-toluenesulfonyl.

b Conversion according to ^31^P NMR spectroscopy after 16 h when the reaction was stopped.

c NMR in fluorobenzene.

**Figure 1:**
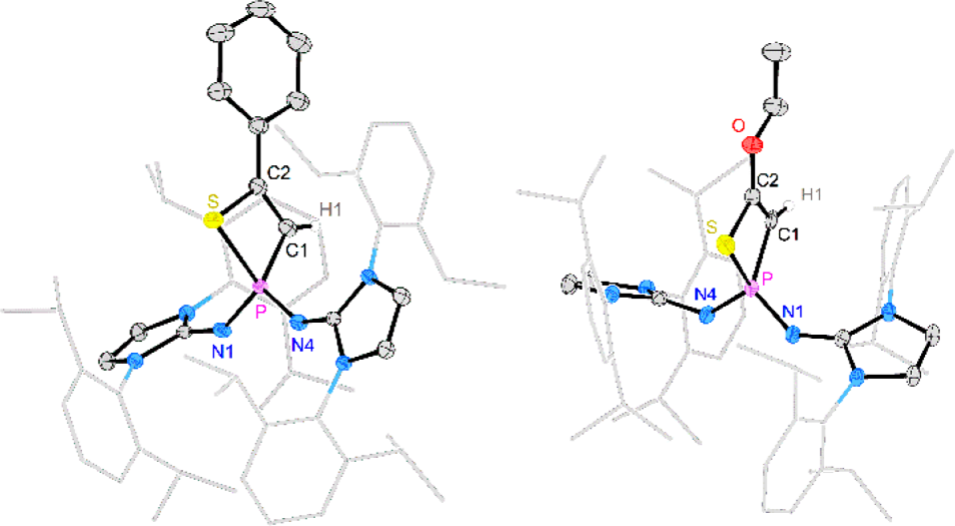
Solid-state structure of [**2a**][BArF_24_] (left) and [**2d**][BArF_24_] (right). Hydrogen atoms (except H1), solvent molecules, and the BArF_24_^–^ anions are omitted for clarity. Ellipsoids are drawn at 50% probability. Dipp groups are shown in wireframe. Selected bond lengths [Å] and angles [°]: [**2a**][BArF_24_]: P–S 2.1541(7), S–C2 1.797(2), C1–C2 1.349(3), P–C1 1.768(2), N1–P 1.576(2), N4–P 1.571(2), P–S–C2 73.61(7), S–C2–C1 107.17(14), C2–C1–P 98.93(14), C1–P–S 80.28(7). [**2d**][BArF_24_]: P–S 2.1665(6), S–C2 1.765(2), C1–C2 1.376(2), P–C1 1.816(2), N1–P 1.5735(14), N4–P 1.6742(13), C2–O 1.327(2), P–S–C2 72.62(6), S–C2–C1 112.03(13), C2–C1–P 93.81(12), C1–P–S 81.52(6).

Single crystals of [**2a**][BArF_24_] were obtained by layering a saturated CH_2_Cl_2_ solution with *n*-pentane. [**2d**][BArF_24_] was crystallized by storing a saturated CH_2_Cl_2_ solution at −40 °C. A single-crystal X-ray diffraction (XRD) study (Figure 1) revealed that the four-membered rings of both thiaphosphete salts are perfectly planar (sum of angles: 360°). The P–S bond length of [**2a**]^+^ (2.154 Å) is shorter than that in the P^III^ 1,2-thiaphosphete **II** (2.161 Å),^43^ as expected for the more electrophilic cationic P^V^ center. Accordingly, the elongated P–S bond (2.167 Å) in [**2d**]^+^ indicates a weaker S–P interaction than in [**2a**]^+^, which is supported by our computational results (vide infra).

## Computational Studies

**Figure 2:**
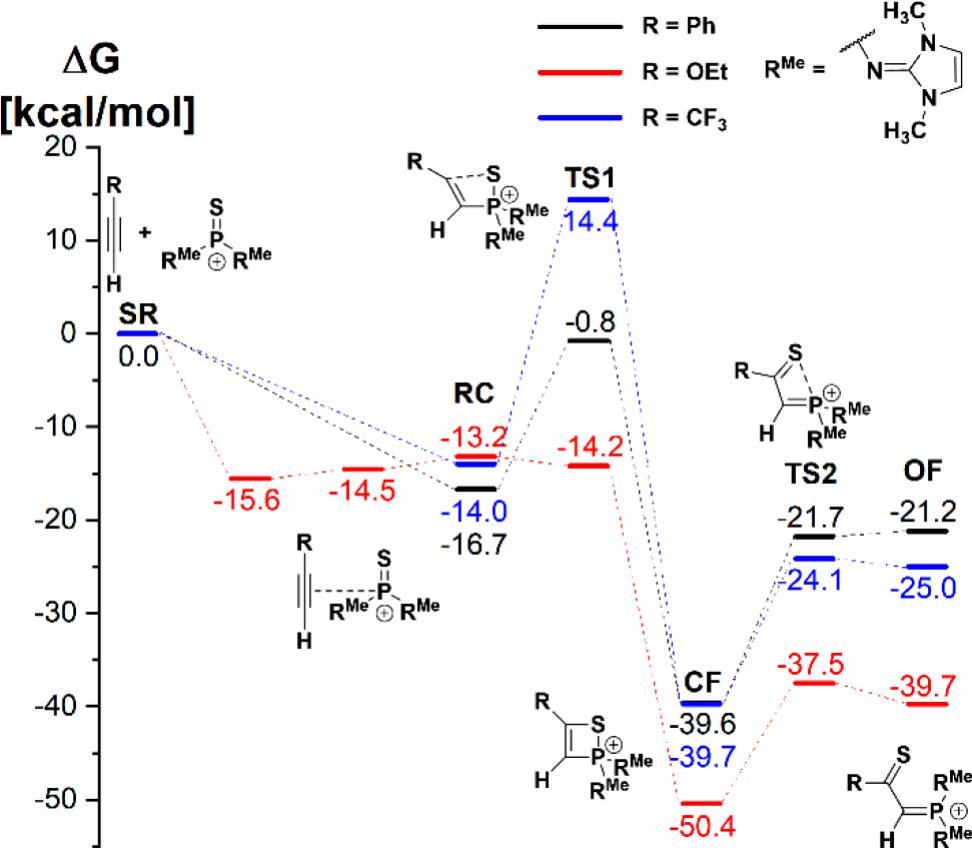
DLPNO–CCSD(T)/def2-TZVPP results including corrections to Gibbs free energy for different reactions of [(R^Me^)_2_PS]^+^ (R^Me^ = 1,3-dimethylimidazolin-2-ylidenamino) with the corresponding alkyne (see legend). Separated reactants (**SR**) have been used as a reference.

We performed DLPNO–CCSD(T)/def2-TZVPP^45−52^ calculations using the simplified thiophosphonium cation [(R^Me^)_2_PS]^+^, which contains methyl groups at the imidazole N atoms instead of the bulky Dipp substituents. Three different model reactions involving phenylacetylene, ethoxyacetylene, and (trifluoromethyl)acetylene were considered as to gain insight into electronic effects on the energy profile (Figure 2). The computed energy barriers of the [2 + 2]-cycloaddition reactions are in line with the experimental observations (cf. Table 1) and show the trend that the electron-rich alkyne ethoxyacetylene reacts much faster than phenylacetylene or (trifluoromethyl)acetylene. The latter has the first transition state of almost 30 kcal/mol, meaning that the cycloaddition reaction would require very harsh conditions. Regardless of the electronic nature of the alkyne, the closed form (**CF**) is thermodynamically favored over the open form (**OF**).

Since we have used the same model substituents R^Me^ in our previous study of the reaction of the oxophosphonium cation [(R^Me^)_2_PO]^+^ with phenylacetylene,^40^ this gives us the opportunity to evaluate how replacing the O atom with the S atom would influence the energy profile. In fact, the first barrier (**TS1**) and the second barrier (**TS2**) are both only ∼1 kcal/mol lower in energy for the thiophosphonium case (cf. Figure 2 and ref (40)). The most notable deviation between the oxo- and thio systems is the energy difference between **CF** and **OF**. In the case of oxophosphonium, the closed form was more stable by 13.3 kcal/mol, while in the case of the thiophosphonium, the closed form was more stable by 18.4 kcal/mol, putting the open form slightly above the transition state.

**Figure 3:**
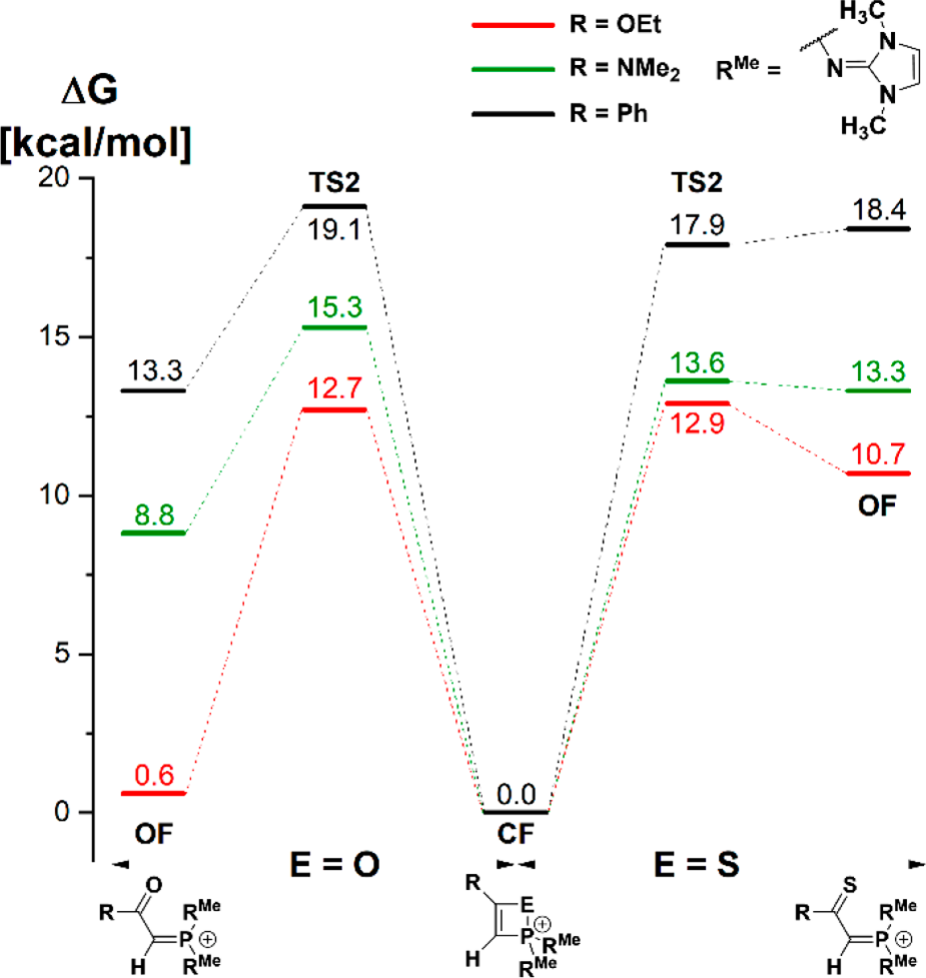
DLPNO–CCSD(T)/def2-TZVPP results including corrections to Gibbs free energy for ring-opening reactions of model oxa- and thiaphosphetes (R^Me^ = 1,3-dimethylimidazolin-2-ylidenamino). The closed forms (**CF**) have been used as a reference.

The heavy atom α,β-unsaturated ketones contain reactive double bonds and thus provide a platform for rich follow-up chemistry. Phosphabutadiene derivatives have been extensively used in cycloaddition reactions for the construction of phosphorus-containing heterocycles,^53−61^ and many examples of P=C–C=O compounds reacting in hetero-Diels–Alder reactions were reported.^62,63^ Since the analogous reactivity with a P=C–C=S moiety is unexplored, we attempted to identify substituent effects that would stabilize this acyclic structure. The rather low transition state with ethoxyacetylene indicates that electron-donating groups might be beneficial in this respect. Hence, the cyclization step was computed for the reaction of oxo- and thiophosphonium ions with acetylene derivatives carrying phenyl, ethoxy, and dimethylamino substituents (Figure 3). The comparison of the relative energy levels of **CF** and **OF** structures indicates that with an increasing number of heavy atoms in the system, the four-membered ring gets stabilized over the α,β-unsaturated ketone structure, which is consistent with the double-bond rule, as heavy atom (p–p)π bonds are formed upon electrocyclic ring-opening. Remarkably, the ethoxy substituent is most effective in facilitating the ring-opening reaction, leading to a thermoneutral reaction for the oxaphosphete system.

## [4 + 2]-Hetero-Diels–Alder Reactions

**Scheme 3:**
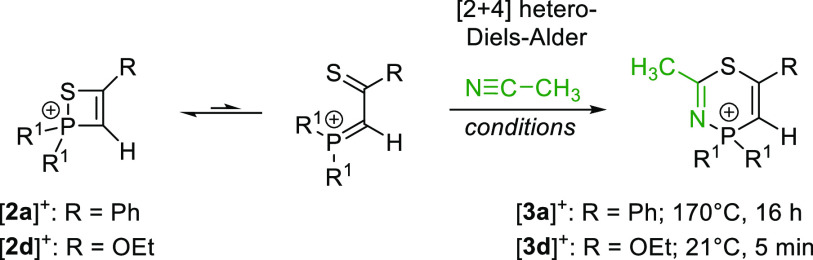
[4 + 2]-Cycloaddition Reactions of [**2a**][BArF_24_] and [**2d**][BArF_24_] with Acetonitrile [BArF_24_]^−^ anions are omitted.

The low energy barrier of 12.9 kcal/mol for the ring-opening reaction of [**2d**]^+^ suggests the possibility of employing the acyclic P=C–C=S platform in hetero-Diels-alder reactions. In fact, dissolving [**2d**][BArF_24_] in acetonitrile gave a clear solution from which the [2 + 4] cycloaddition product [**3d**][BArF_24_] precipitates within 5 min (Scheme 3).

The formation of the six-membered thiazaphosphinine ring in [**3d**]^+^ is confirmed by the deshielded doublet of the S–C–N carbon atom at 164.6 ppm (^2^*J*_CP_ = 6 Hz) in the ^13^C NMR spectrum. The ^31^P resonance (−22.2 ppm) appears at higher frequency compared to the precursor [**2d**]^+^ (−35.7 ppm). The thiaphosphete salt [**2a**][BArF_24_] shows no reaction with acetonitrile below 60 °C and only very slow conversion at 100 °C. Heating the mixture to 170 °C for 16 h gave [**3a**][BArF_24_] in quantitative yield. The ^31^P NMR resonance of the heterocycle appears at −34.0 ppm. The different reaction conditions for the ring expansion reactions indicate that ring-opening of the thiaphosphetes is required prior to the hetero-Diels–Alder reactions, which, in agreement with the computational results, is more easily accessible for [**2d**]^+^ than for [**2a**]^+^. The analogous ring expansion reaction with oxaphosphetes proceeds at lower temperature than that with thiaphosphetes,^40^ which again is consistent with the energy barrier of the electrocyclic ring-opening reaction.

**Figure 4:**
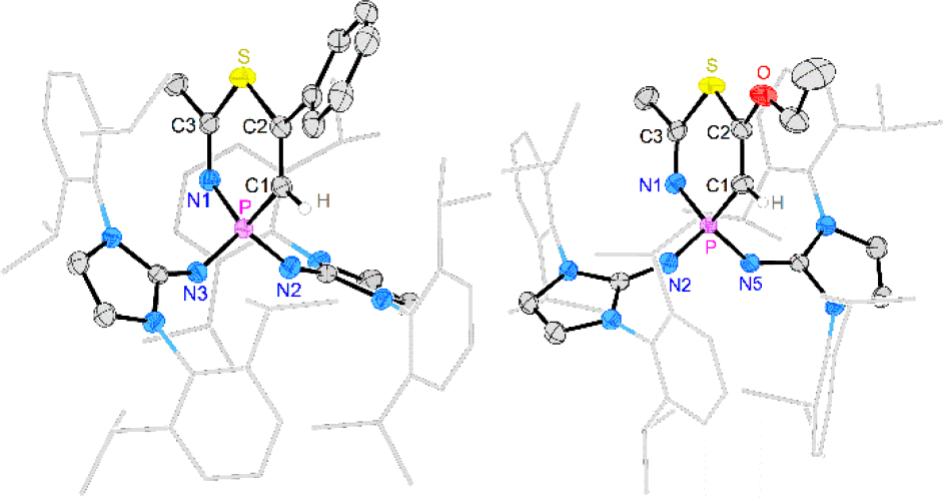
Solid-state structures of [**3a**][BArF_24_] (left) and [**3d**][BArF_24_] (right). Hydrogen atoms (except H1) and the BArF_24_^–^ anions are omitted for clarity. Ellipsoids are drawn at 50% probability. Dipp groups are shown in wireframe. Selected bond lengths [Å] and angles [°]: [**3a**][BArF_24_]: P–N1 1.662(2), N1–C3 1.268(3), S–C3 1.768(2), S–C2 1.752(2), C1–C2 1.338(3), P–C1 1.761(2), N2–P 1.565(2), N3–P 1.576(2), P–N1–C3 127.4(2), N1–C3–S 128.9(2), C3–S–C2 104.96(11), S–C2–C1 124.5(2), C2–C1–P 126.9(2), C1–P–N1 107.21(11). [**3d**][BArF_24_]: P–N1 1.671(2), N1–C3 1.264(3), S–C3 1.766(3), S–C2 1.752(3), C1–C2 1.336(3), P–C1 1.758(2), N2–P 1.573(2), N5–P 1.587(2), P–N1–C3 127.5(2), N1–C3–S 128.5(2), C3–S–C2 104.37(11), S–C2–C1 126.8(2), C2–C1–P 124.4(2), C1–P–N1 108.12(11).

Single-crystal XRD studies of [**3a**][BArF_24_] and [**3d**][BArF_24_] revealed planar thiazaphosphinine rings (sum of angles: 720°) flanked by the bulky substituents at the phosphorus atom (Figure 4). Both structures have very similar geometrical parameters. The C–N bonds ([**3a**]^**+**^: 1.268 Å, [**3d**]^**+**^: 1.264 Å) and the C–C bonds ([**3a**]^**+**^: 1.338 Å, [**3d**]^**+**^: 1.336 Å) of the six-membered rings are in the range of double bonds.^64^ The hexagonal shape of the heterocycles is significantly distorted due to the small bond angles centered around the sulfur ([**3a**]^**+**^: 105°, [**3d**]^**+**^: 104°) and phosphorus ([**3a**]^**+**^: 107°, [**3d**]^**+**^: 108°) atoms.

## Conclusions

The P=S double bond of a Lewis-base-free thiophosphonium ion undergoes [2 + 2]-cycloadditions with terminal alkynes to generate thiaphosphete cations [**2a**–**f**]^+^. The four-membered rings undergo electrocyclic ring-opening reactions to the acyclic 1-phospha-4-thia-butadiene structure, which was used to generate the six-membered heterocycles [**3a**]^+^ and [**3d**]^+^ via [4 + 2]-hetero-Diels–Alder reactions. Quantum chemical calculations reveal that electron-donating substituents at the alkyne facilitate both the [2 + 2]-cycloaddition reaction and the ring-opening reaction, while an increasing number of heavy atoms generally stabilizes the four-membered ring structure.

The presented heavy congener of a thioketone–alkyne metathesis is an appealing example for the diagonal relationship between carbon and phosphorus in the periodic table. The great potential of the R_2_P^+^ fragment to act in a thermoneutral fashion in bond metathesis reactions is indicated by the ring-opening reaction of the ethoxy substituted oxaphosphete. Further studies into this direction will be reported in due course.

## Supporting Information Available

Synthetic procedures, NMR spectra, mass spectrometry data, crystallographic data, and computational details (PDF)

## Accession Codes

CCDC 2094371–2094374 contain the supplementary crystallographic data for this paper. These data can be obtained free of charge via www.ccdc.cam.ac.uk/data_request/cif, or by emailing data_request@ccdc.cam.ac.uk, or by contacting The Cambridge Crystallographic Data Centre, 12 Union Road, Cambridge CB2 1EZ, UK; fax: +44 1223 336033.
