# Supplementary material for: Thiophosphonium–Alkyne Cycloaddition Reactions: A Heavy Congener of the Carbonyl–Alkyne Metathesis
Source: Inorg Chem. 2021 Sep 15;60(19):14509–14. doi: 10.1021/acs.inorgchem.1c02076 (PMC8493552; doi:10.1021/acs.inorgchem.1c02076)
Supplement: Supplementary file 1 — ic1c02076_si_001.pdf [file ic1c02076_si_001.pdf]

Supplementary Material for

Thiophosphonium-Alkyne Cycloaddition Reactions: A Heavy Congener of the Carbonyl-Alkyne Metathesis

Pawel Löwe,[a] Milica Feldt,[b] Maike B. Röthel,[a] Lukas F. B. Wilm,[a] and Fabian Dielmann[a,c]*

[a]Institut für Anorganische und Analytische Chemie, Westfälische Wilhelms-Universität Münster, Corrensstrasse 28-30, 48149 Münster (Germany)

[b]Theoretische Organische Chemie, Organisch-Chemisches Institut and Center for Multiscale Theory and Computation, Westfälische Wilhelms-Universität Münster, Corrensstraße 36, 48149 Münster, (Germany)

[c]Department of General, Inorganic and Theoretical Chemistry, Leopold-Franzens-Universität Innsbruck, Innrain 80-82, 6020 Innsbruck (Austria)

E-mail: Fabian.Dielmann@uibk.ac.at

Homepage: https://www.uibk.ac.at/aatc/mitarbeiter/dielmann

CONTENTS:

1 Experimental procedures **3**

1.1 Synthetic Details 3

1.3 Preparation of [**2a**][$BArF_{24}$] 4

1.4 Reactivity study of [**1**][OTf] with phenylacetylene 8

1.5 Preparation of [**2b**][$BArF_{24}$] 9

1.6 Preparation of [**2c**][$BArF_{24}$] 11

1.7 Preparation of [**2d**][$BArF_{24}$] 14

1.8 Preparation of [**2e**][$BArF_{24}$] 18

1.9 Preparation of [**2f**][$BArF_{24}$] 22

1.10 Preparation of [**3a**][$BArF_{24}$] 23

1.11 Preparation of [**3d**][$BArF_{24}$] 28

2 X-ray Diffraction Studies **32**

2.1 Crystal structure data of compound [**2a**][$BArF_{24}$] 33

2.2 Crystal structure data of compound [**2d**][$BArF_{24}$] 34

2.3 Crystal structure data of compound [**3a**][$BArF_{24}$] 35

2.4 Crystal structure data of compound [**3d**][$BArF_{24}$] 36

3 Computational studies **37**

3.1 General 37

3.2 Calculated energies 38

3.3 Natural population Analysis 42

3.4 Computed Energies (au) and Cartesian Coordinates (Å) 44

4 References **65**

1 Experimental procedures

1.1 Synthetic Details

General remarks: All manipulations were performed under an inert atmosphere of dry argon, using standard Schlenk and drybox techniques. Dry and oxygen-free solvents were employed. All glassware was oven-dried at 160 °C prior to use. ^{1}H, ^{11}B, ^{13}C, ^{19}F and ^{31}P NMR spectra were recorded at 300 K on Agilent DD2 600, Bruker AVANCE I 400, Bruker AVANCE III 400 or Bruker AVANCE II 200 spectrometers. Chemical shifts are given in parts per million (ppm) relative to $SiMe_4$ (^{1}H, ^{13}C), 15% $BF_3{\cdot}OEt_2$ in $CDCl_3$ (^{11}B), CCl_3F (^{19}F), 85% H_3PO_4 (^{31}P) and they were referenced to the residual solvent signals (CD_2Cl_2: ^{1}H $\delta_H = 5.32$, ^{13}C $\delta_C = 53.84$; CD_3CN: $\delta_H = 1.94$, ^{13}C $\delta_C = 118.26$) or internally by the instrument after locking and shimming to the deuterated solvent (^{11}B, ^{19}F, ^{31}P). Chemical shifts (δ) are reported in ppm. NMR multiplicities are abbreviated as follows: s = singlet, d = doublet, t = triplet, p = pentet, sept = septet, m = multiplet, br = broad signal. Mass spectrometry was recorded using an Orbitrap LTQ XL (Thermo Scientific) spectrometer.

Safety remarks: The use of a burst shield is strongly recommended for all reactions that require heating of closed Schlenk flasks above the boiling point of the respective solvent!

Reagents and Handling: All compounds were purchased from commercial sources (Sigma Aldrich, Alfa Aesar, Tokyo Chemical Industry, Activate Scientific) and used as received, if not stated differently. Liquid alkynes were dried by storing them over 4 Å molecular sieves, solid alkynes were dried *in vacuo* and stored under argon atmosphere. Thiophosphonium salts [**1**][$BArF_{24}$] and [**1**][OTf] were synthesized according to literature procedures.[1] For the synthesis of the thiophosphonium salts, 2,6-Diisopropylaniline was obtained from Alfa Aesar (technical grade) and distilled. After distillation it still contained two isomeric impurities (2.1% and 0.6%, respectively, confirmed by GC-MS and ^{1}H NMR). These isomers are also found in the synthesized compounds.

1.3 Preparation of [2a][BArF24]

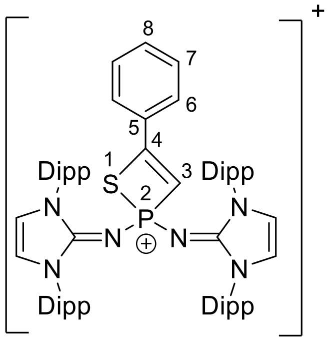

$[BArF_{24}]^-$ or $[OTf]^-$

[**1**][$BArF_{24}$] (0.173 mmol, 300 mg, 1.00 eq.) was dissolved in fluorobenzene and phenylacetylene (0.190 mmol, 17.7 mg, 1.10 eq.) was added to the solution. The reaction was heated in a sealed tube at 120 h for 16 h. All volatiles were removed *in vacuo*. The product was obtained as an off-white solid.

Yield: Quantitative.

^{1}H NMR (CD_2Cl_2, 400 MHz, 300 K): δ (ppm) = 7.76 (m, 8 H, $BArF_{24}$; *ortho*), 7.58 (m, 4 H, $BArF_{24}$; *para*), 7.44 (t, $^3J_{HH}$ = 7.8 Hz, 4 H, *p*-Dipp), 7.43 (m, 1 H, 8), 7.35 (m, 2 H, 7), 7.23-7.21 (m, 4 H, *m*-Dipp), 7.11-7.09 (m, 4 H, *m*-Dipp), 6.91 - 6.89 (m, 2 H, 6), 6.64 (s, 4 H, N-CH=CH-N), 3.80 (d, $^3J_{HH}$ = 18.9 Hz, 1 H, 3), 2.63 (sept, $^3J_{HH}$ = 6.9 Hz, 4 H, *i*Pr-C*H*), 2.53 (sept, $^3J_{HH}$ = 6.9 Hz, 4 H, *i*Pr-C*H*), 1.04 (d, $^3J_{HH}$ = 6.9 Hz, 12 H, *i*Pr-C*H*$_3$), 1.03 (d, $^3J_{HH}$ = 6.9 Hz, 12 H, *i*Pr-C*H*$_3$), 0.99 (d, $^3J_{HH}$ = 6.9 Hz, 12 H, *i*Pr-C*H*$_3$), 0.83 (d, $^3J_{HH}$ = 6.9 Hz, 12 H, *i*Pr-C*H*$_3$).

^{13}C{^{1}H} NMR (CD_2Cl_2, 101 MHz, 300 K): δ (ppm) = 162.2 (q, $^1J_{CB}$ = 50 Hz, $BArF_{24}$; ipso), 153.4 (d, $^2J_{CP}$ = 5 Hz, 4), 142.4 (d, $^2J_{CP}$ = 21 Hz, N-C-N), 135.3 ($BArF_{24}$; *ortho*), 132.0 (*i*-Dipp), 131.7 (8), 131.4 (d, $^3J_{CP}$ = 33 Hz, 5), 131.2 (*p*-Dipp), 129.4 (qq, $^2J_{CF}$ = 32 Hz, $^4J_{CF}$ = 3 Hz, $BArF_{24}$; *meta*), 128.5 (7) 127.4 (d, $^4J_{CP}$ = 2 Hz, 6), 125.3 (*m*-Dipp), 125.1 (q, $^1J_{CF}$ = 272 Hz, $BArF_{24}$; *C*F$_3$), 124.9 (*m*-Dipp), 120.3 (d, $^1J_{CP}$ = 106 Hz, 3), 117.9 (sept, J_{CF} = 4 Hz, $BArF_{24}$; *para*), 29.3 (*i*Pr-*C*H), 29.2 (*i*Pr-*C*H), 24.9 (*i*Pr-*C*H$_3$), 24.6 (*i*Pr-*C*H$_3$), 23.1 (*i*Pr-*C*H$_3$), 23.1 (*i*Pr-*C*H$_3$).

^{11}B NMR (CD_2Cl_2, 128 MHz, 300 K): δ (ppm) = -6.6.

^{19}F NMR (CD_2Cl_2, 376 MHz, 300 K): δ (ppm) = -62.8.

^{31}P NMR (CD_2Cl_2, 162 MHz, 300 K): δ (ppm) = -36.1 (d, $^2J_{PH}$ = 19 Hz).

HR-MS(ESI): m/z calculated for $[C_{62}H_{78}N_6PS]^+$ as [**2a**]$^+$: 969.57408 found: 969.57475. Fitting isotope pattern: Yes.

Single crystal X-ray diffraction analysis: Single crystals suitable for X-ray diffraction analysis were obtained by layering saturated solution of [**2a**][$BArF_{24}$] in CH_2Cl_2 with n-pentane.

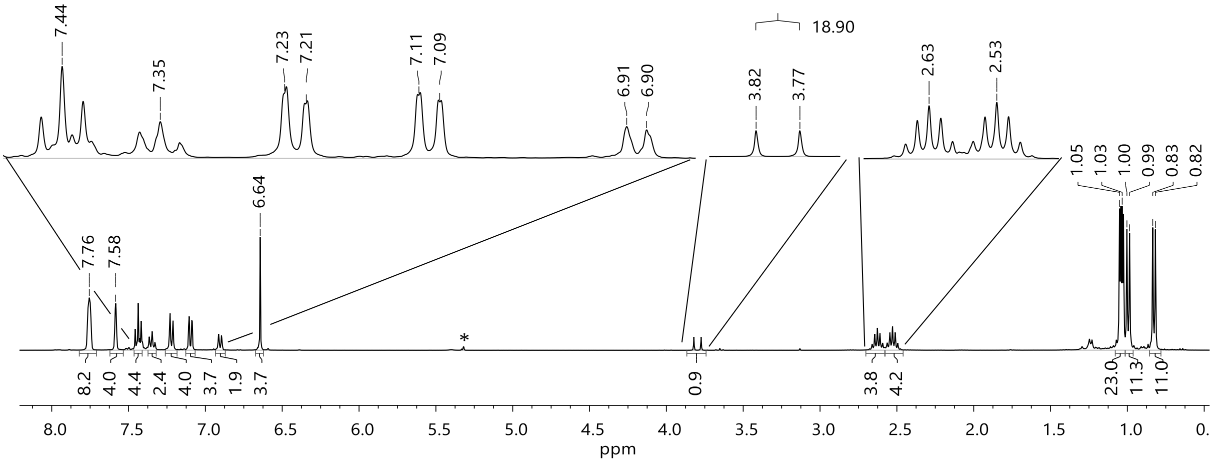

Figure S 1: ^{1}H NMR spectrum of [**2a**][$BArF_{24}$] in CD_2Cl_2. *solvent residue signal.

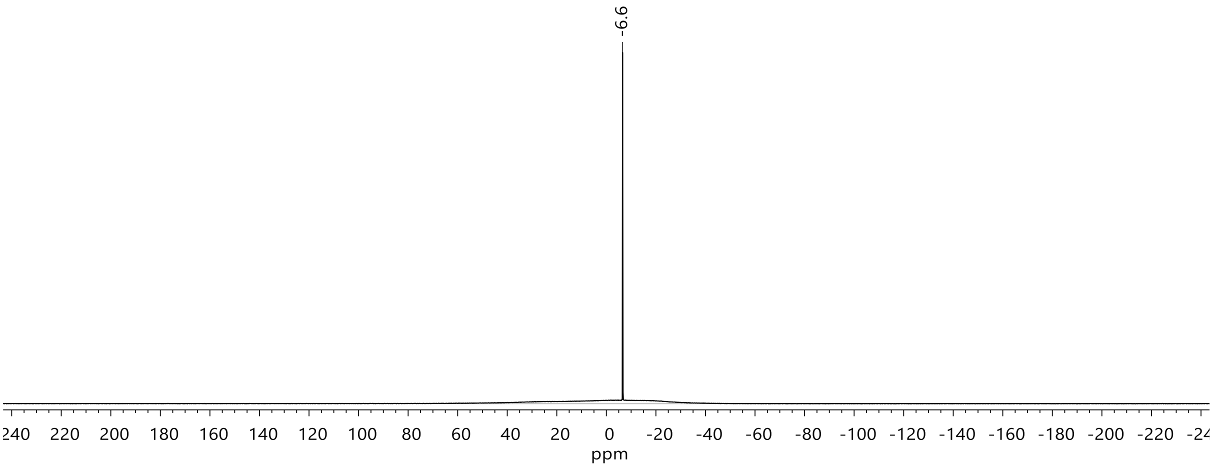

Figure S 2: ^{11}B NMR spectrum of [**2a**][BArF$_{24}$] in CD_2Cl_2.

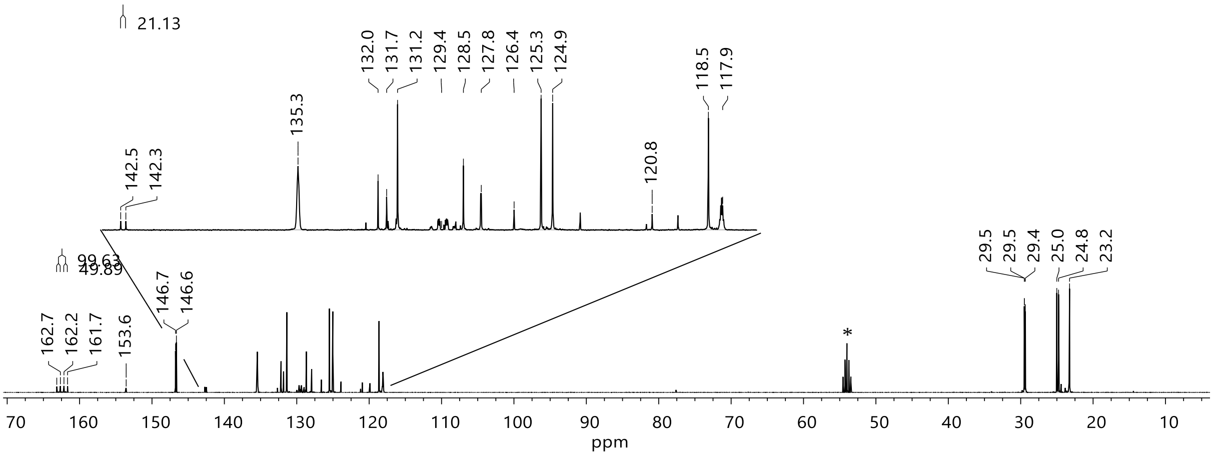

Figure S 3: ^{13}C{^{1}H} NMR spectrum of [**2a**][BArF$_{24}$] in CD_2Cl_2. *solvent residue signal.

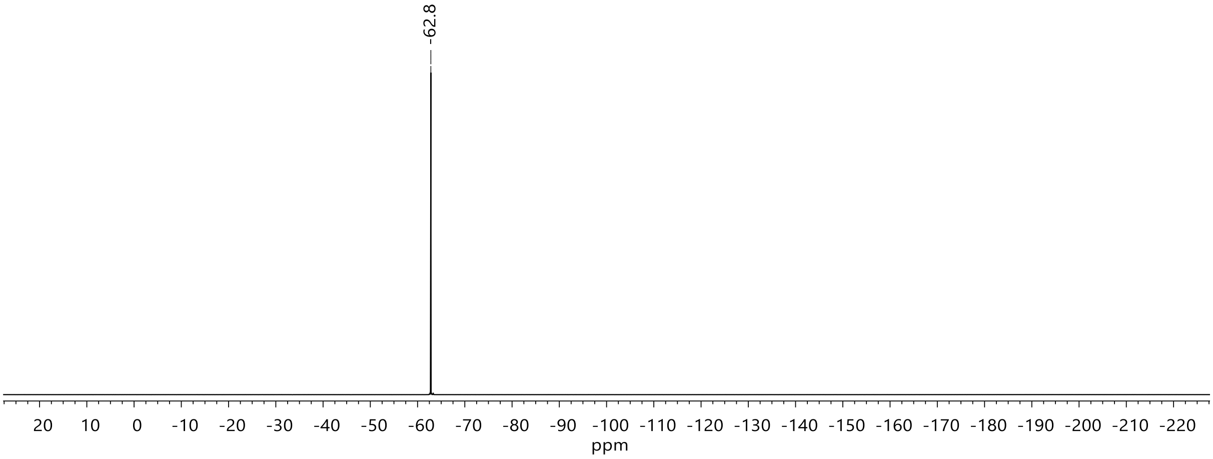

Figure S 4: ^{19}F NMR spectrum of [**2a**][BArF$_{24}$] in CD_2Cl_2.

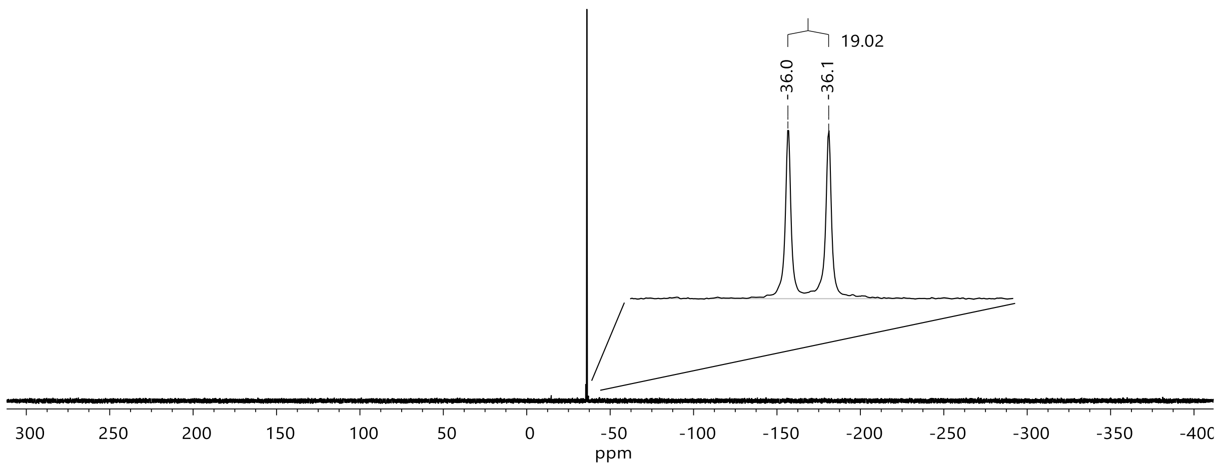

Figure S 5: ^{31}P NMR spectrum of [**2a**][BArF$_{24}$] in CD_2Cl_2.

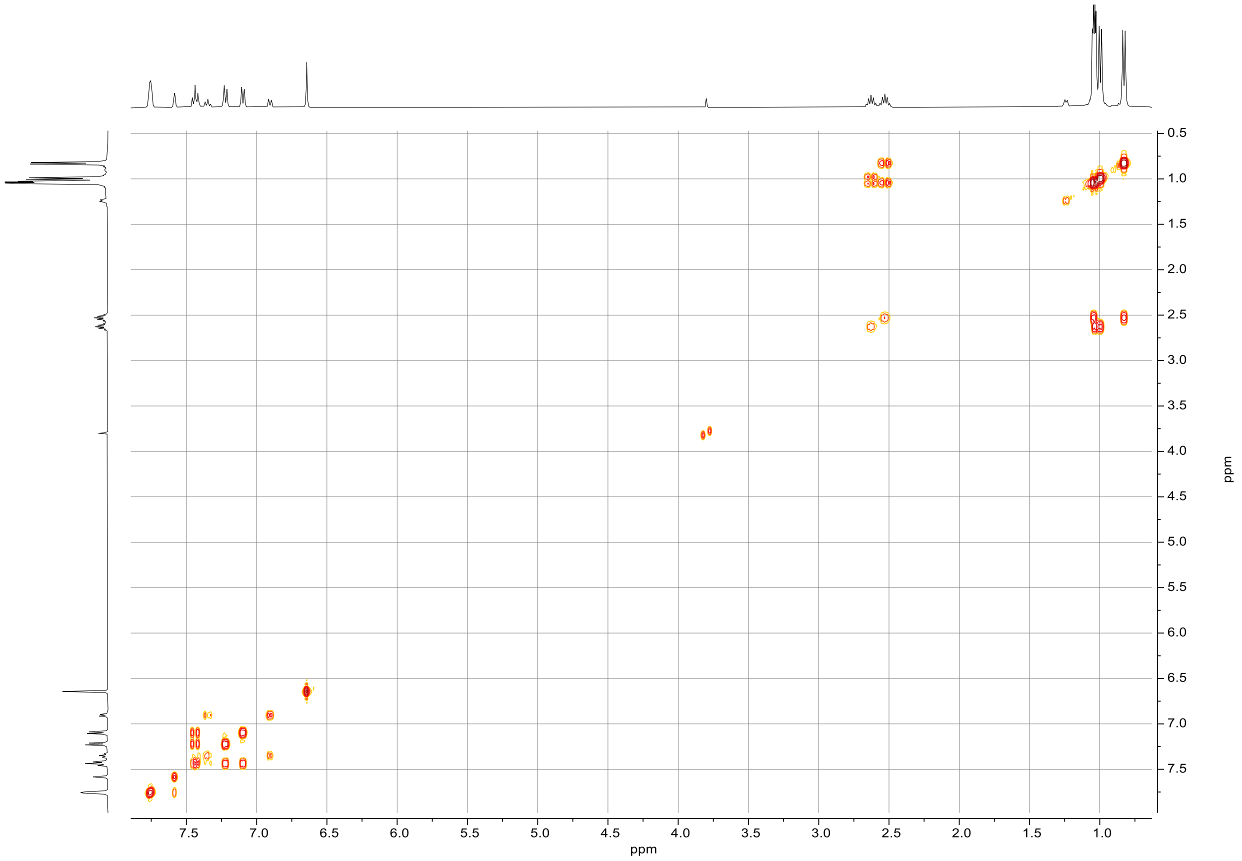

Figure S 6: ^{1}H/^{1}H COSY NMR spectrum of [**2a**][BArF$_{24}$] in CD_2Cl_2.

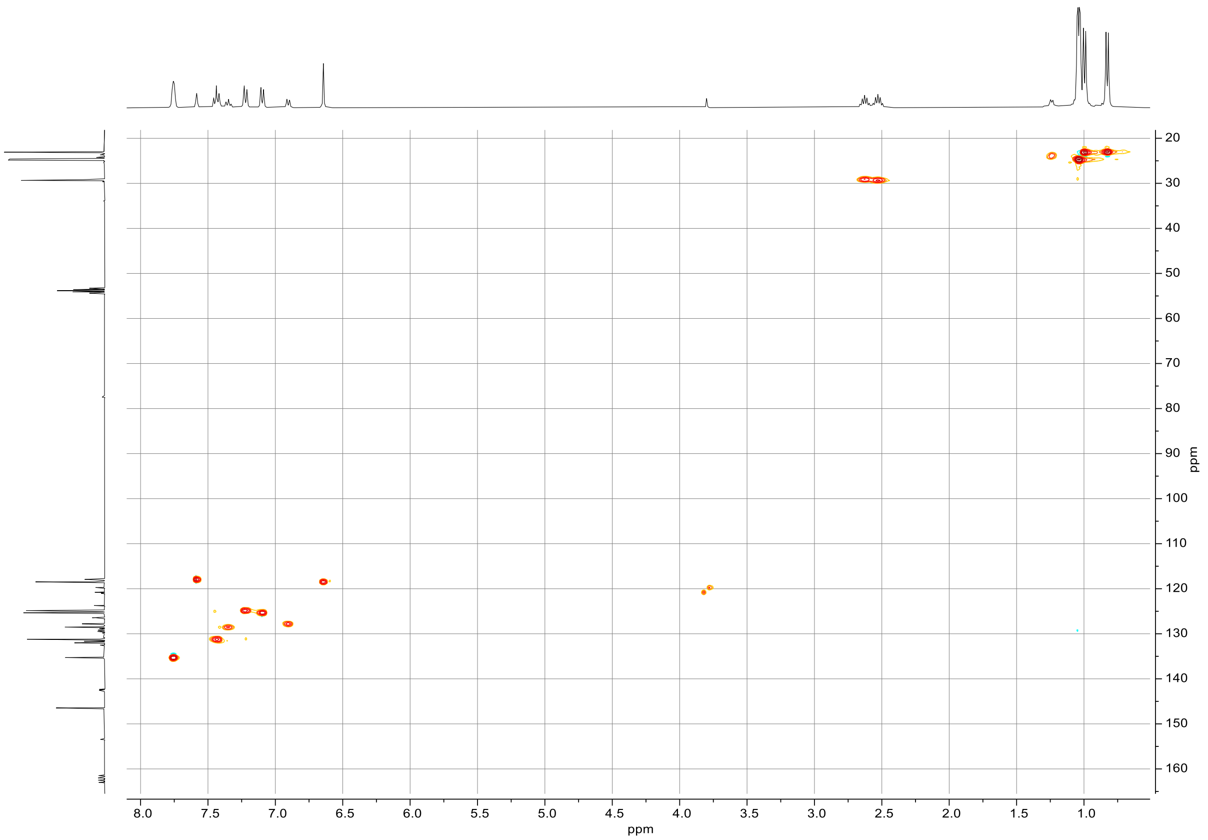

Figure S 7: $^{1}H/^{13}C$ HSQC spectrum o of [**2a**][$BArF_{24}$] in CD_2Cl_2.

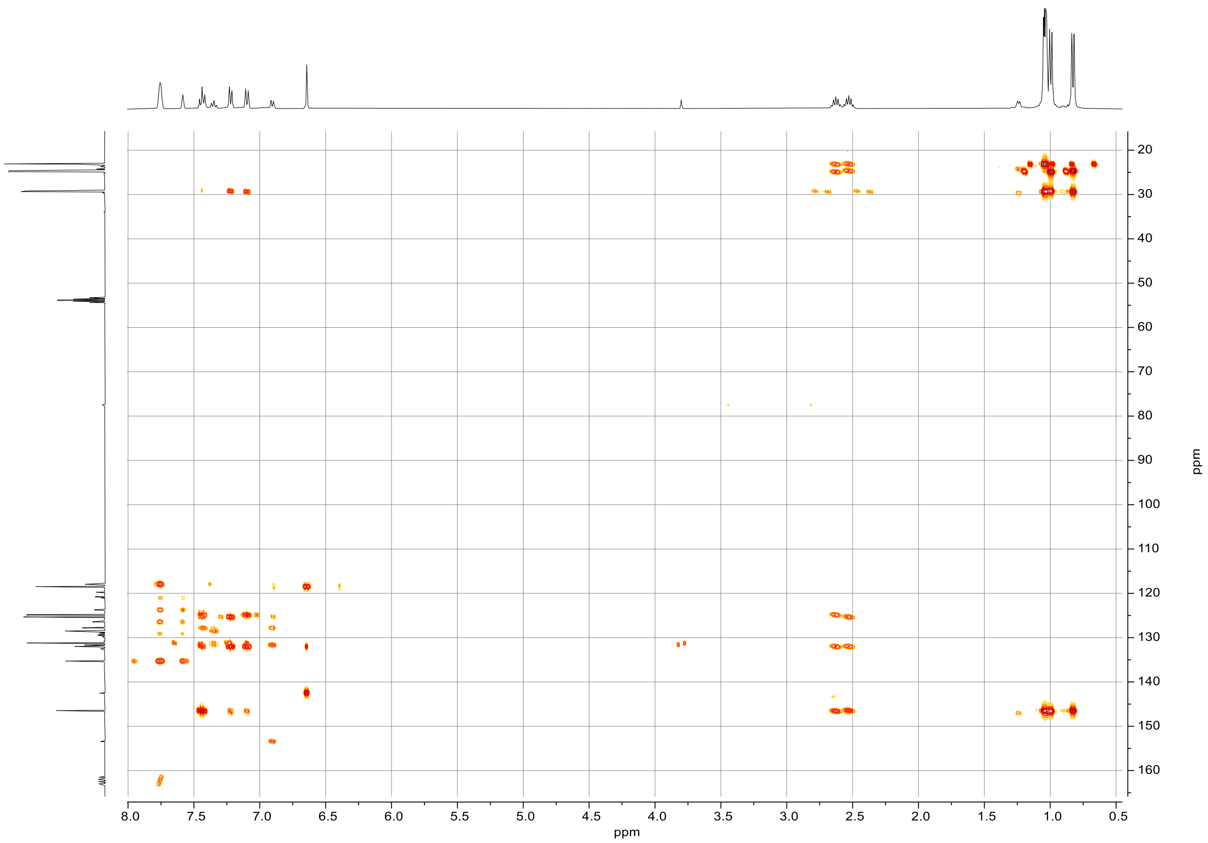

Figure S 8: $^{1}H/^{13}C$ HMBC NMR spectrum of [**2a**][$BArF_{24}$] in CD_2Cl_2.

1.4 **Reactivity study of [1][OTf] with phenylacetylene**

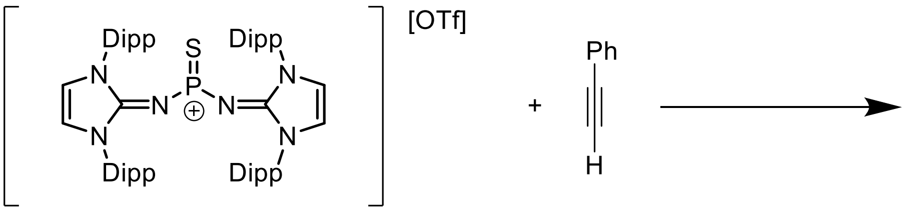

$[(R^1)_2PS][OTf]$ ([**1**][OTf]) (0.320 mmol, 317 mg, 1.00 eq.) was dissolved in fluorobenzene and phenylacetylene (0.320 mmol, 33.0 mg, 1.00 eq.) was added to the solution. The reaction was heated in a sealed vessel at 120 °C for 65 h. All volatiles were removed *in vacuo*. The residue was dissolved in CD_3CN. The ^{31}P NMR spectrum of the reaction mixture indicated the formation of several phosphorus species. According to the characteristic doublet at -36.6 ppm in the ^{31}P NMR spectrum, the desired four-membered heterocycle [**2a**][OTf] is formed in about 86 % yield.

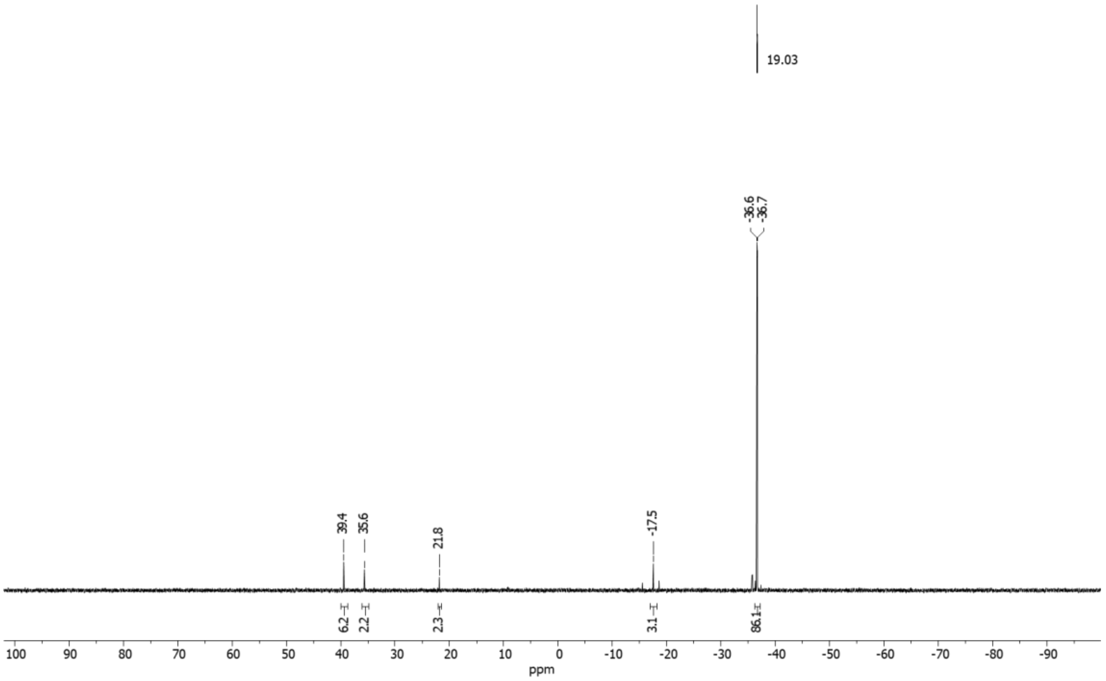

Figure S 9: ^{31}P NMR spectrum of the reaction of [**1**][OTf] with phenylacetylene in CD_3CN.

1.5 **Preparation of [2b][$BArF_{24}$]**

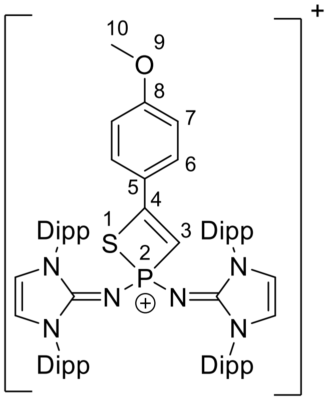

[$BArF_{24}$]$^-$

[**1**][$BArF_{24}$] (0.014 mmol, 25 mg, 1.0 eq.) was dissolved in fluorobenzene and 4-methoxyphenylacetylene (0.014 mmol, 1.9 mg, 1.0 eq.) was added to the solution. The reaction was heated in a sealed tube at 60 °C for 16 h. All volatiles were removed *in vacuo*. The product was obtained as a pale-yellow solid .

Yield: quantitative.

^{1}H NMR (CD_2Cl_2, 400 MHz, 300 K): δ (ppm) = 7.74 (m, 8 H, $BArF_{24}$; *ortho*), 7.57 (m, 4 H, $BArF_{24}$; *para*), 7.43 (t, $^3J_{HH}$ = 7.8 Hz, 4 H, *p*-Dipp), 7.21 (dd, $^3J_{HH}$ = 7.8 Hz, $^4J_{HH}$ = 1.4 Hz, 4 H, *m*-Dipp), 7.09 (dd, $^3J_{HH}$ = 7.8 Hz, $^4J_{HH}$ = 1.4 Hz, 4 H, *m*-Dipp), 6.83 (s, 2 H, 6), 6.83 (s, 2 H, 7), 6.62 (s, 4 H, N-C*H*=C*H*-N), 3.61 (d, $^2J_{HP}$ = 19.1 Hz, 1 H, 3), 3.85 (s, 3H, 10), 2.62 (sept, $^3J_{HH}$ = 6.9 Hz, 4 H, *i*Pr-C*H*), 2.53 (sept, $^3J_{HH}$ = 6.9 Hz, 4 H, *i*Pr-C*H*), 1.04 (d, $^3J_{HH}$ = 6.9 Hz, 12 H, *i*Pr-C*H*$_3$), 1.02 (d, $^3J_{HH}$ = 6.9 Hz, 12 H, *i*Pr-C*H*$_3$), 0.98 (d, $^3J_{HH}$ = 6.9 Hz, 12 H, *i*Pr-C*H*$_3$), 0.83 (d, $^3J_{HH}$ = 6.9 Hz, 12 H, *i*Pr-C*H*$_3$).

^{31}P NMR (CD_2Cl_2, 162 MHz, 300 K): δ (ppm) = -35.2 (d, $^2J_{PH}$ = 19 Hz).

HR-MS(ESI): m/z was calculated for [$C_{63}H_{80}N_6OPS$]$^+$ as [**2b**]$^+$: 999.58465 found was: 999.58319. Fitting isotope pattern: Yes.

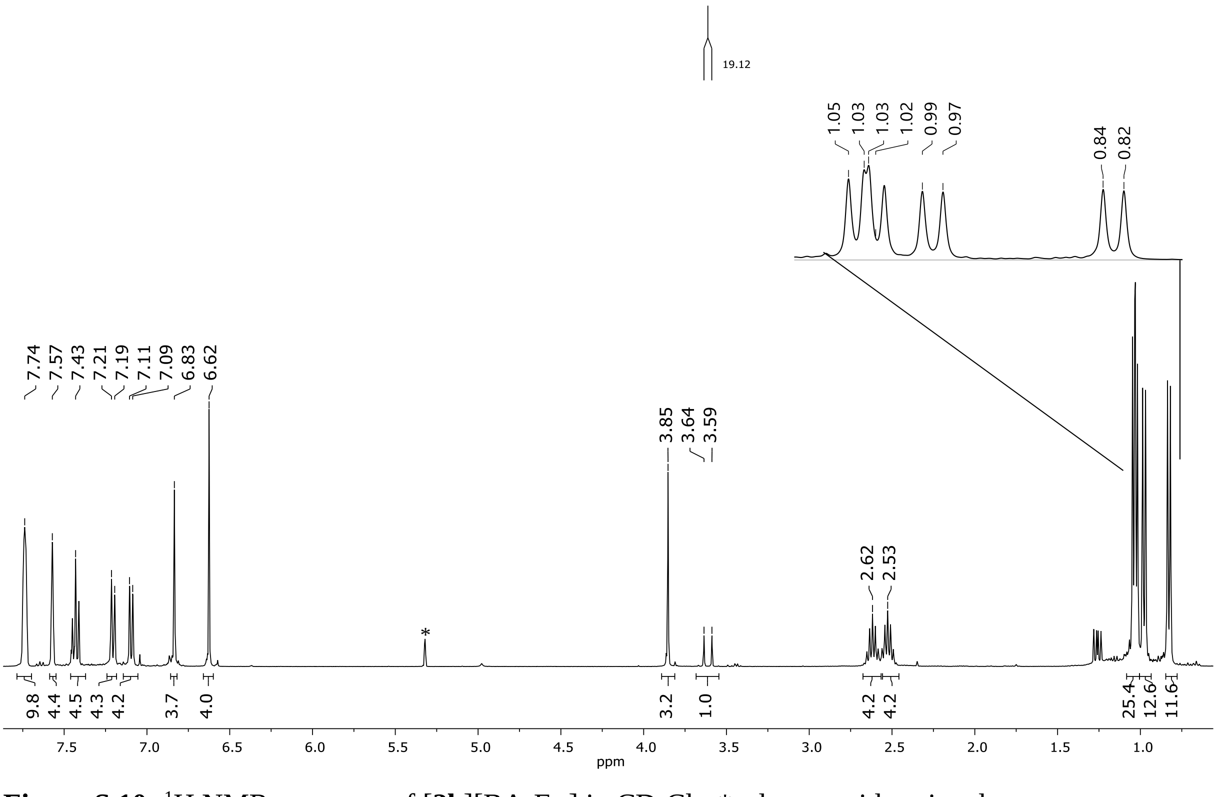

Figure S 10: ^{1}H NMR spectrum of [**2b**][$BArF_{24}$] in CD_2Cl_2. *solvent residue signal.

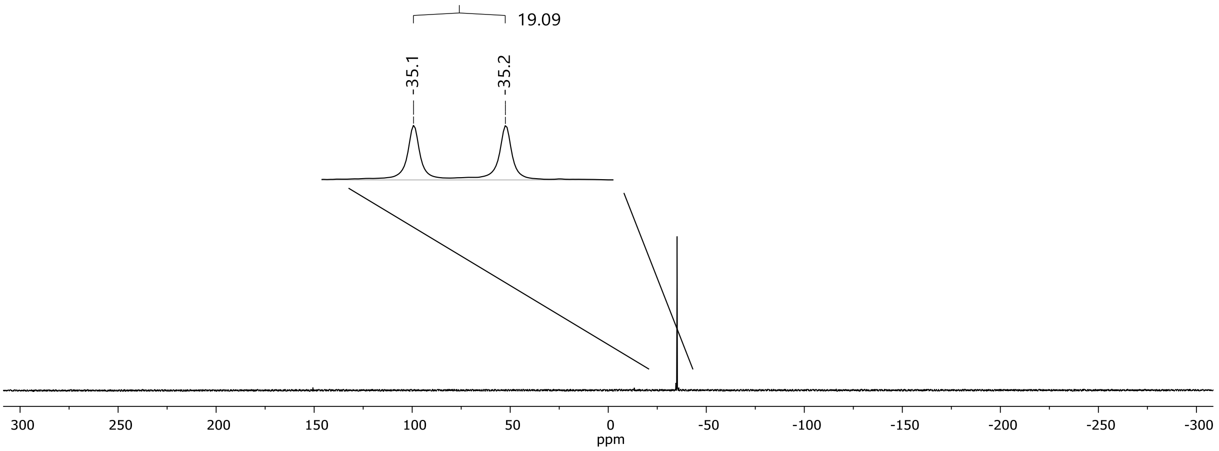

Figure S 11: ^{31}P NMR spectrum of [**2b**][BArF$_{24}$] in CD_2Cl_2.

1.6 Preparation of [2c][BArF$_{24}$]

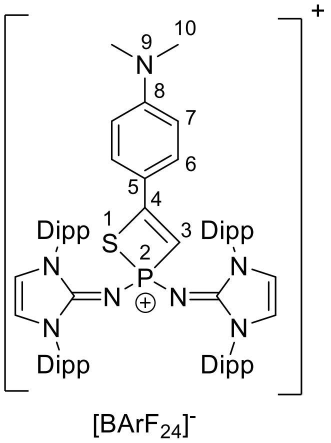

[**1**][BArF$_{24}$] (0.035 mmol, 60 mg, 1.0 eq.) was dissolved in fluorobenzene and 4-Dimethylaminophenylacetylene (0.035mmol, 5.0 mg, 1.0 eq.) was added to the solution. After 2 h, all volatiles were removed *in vacuo*. The product was obtained as a green solid.

Yield: Quantitative.

^{1}H NMR (CD_2Cl_2, 500 MHz, 300 K): δ (ppm) = 7.75 (m, 8 H, BArF$_{24}$; *ortho*), 7.58 (m, 4 H, BArF$_{24}$; *para*), 7.44 (t, $^{3}J_{HH}$ = 7.8 Hz, 4 H, *p*-Dipp), 7.21 (dd, $^{3}J_{HH}$ = 7.8 Hz, $^{4}J_{HH}$ = 1.4 Hz, 4 H, *m*-Dipp), 7.12 (dd, $^{3}J_{HH}$ = 7.8 Hz, $^{4}J_{HH}$ = 1.4 Hz, 4 H, *m*-Dipp), 6.76 (m, 2 H, 6), 6.61 (s, 4 H, N-C*H*=C*H*-N), 6.57 (m, 2 H, 7), 3.48 (d, $^{2}J_{HP}$ = 19.6 Hz, 1 H, 3), 3.04 (s, 6H, 10) 2.63 (sept, $^{3}J_{HH}$ = 6.9 Hz, 4 H, *i*Pr-C*H*), 2.55 (sept, $^{3}J_{HH}$ = 6.9 Hz, 4 H, *i*Pr-C*H*), 1.04 (d, $^{3}J_{HH}$ = 6.9 Hz, 12 H, *i*Pr-CH_3), 1.03 (d, $^{3}J_{HH}$ = 6.9 Hz, 12 H, *i*Pr-CH_3), 0.97 (d, $^{3}J_{HH}$ = 6.9 Hz, 12 H, *i*Pr-CH_3), 0.84 (d, $^{3}J_{HH}$ = 6.9 Hz, 12 H, *i*Pr-CH_3).

^{13}C{^{1}H} NMR (CD_2Cl_2, 126 MHz, 300 K): δ (ppm) = 164.3 (d, $^{2}J_{CP}$ = 29 Hz, 4), 162.2 (q, $^{1}J_{CB}$ = 50 Hz, BArF$_{24}$; ipso), 152.5 (8), 146.7 (*o*-Dipp), 146.5 (*o*-Dipp), 142.6 (d, $^{2}J_{CP}$ = 20 Hz, N-C-N), 135.3 (BArF$_{24}$; *ortho*), 132.2 (*p*-Dipp), 131.0 (6), 129.7 (*i*-Dipp), 129.3 (qq, $^{2}J_{CF}$ = 32 Hz, $^{4}J_{CF}$ = 3 Hz, BArF$_{24}$; *meta*), 125.2 (*m*-Dipp), 125.1 (q, $^{1}J_{CF}$ = 272 Hz, BArF$_{24}$; *C*F$_3$), 124.8 (*m*-Dipp), 118.3 (N-*C*H=*C*H-N), 117.9 (sept, $^{3}J_{CF}$ = 4 Hz, BArF$_{24}$; *para)*, 115.6 (d, $^{3}J_{CP}$ = 21 Hz, 5), 113.5 (d, $^{1}J_{CP}$ = 109 Hz, 3), 110.7 (7), 40.2 (10), 29.3 (*i*Pr-*C*H), 29.2 (*i*Pr-*C*H), 24.9 (*i*Pr-*C*H$_3$), 24.6 (*i*Pr-*C*H$_3$), 23.1 (*i*Pr-*C*H$_3$), 23.1 (*i*Pr-*C*H$_3$).

^{11}B NMR (CD_2Cl_2, 160 MHz, 300 K): δ (ppm) = -6.6.

^{19}F NMR (CD_2Cl_2, 471 MHz, 300 K): δ (ppm) = -62.8.

^{31}P NMR (CD_2Cl_2, 202 MHz, 300 K): δ (ppm) = -32.2 (d, $^{2}J_{PH}$ = 20 Hz).

HR-MS(ESI): m/z was calculated for [$C_{64}H_{83}N_7PS$]$^+$ as [**2c**]$^+$: 1012.61628 found was: 1012.61582. Fitting isotope pattern: Yes.

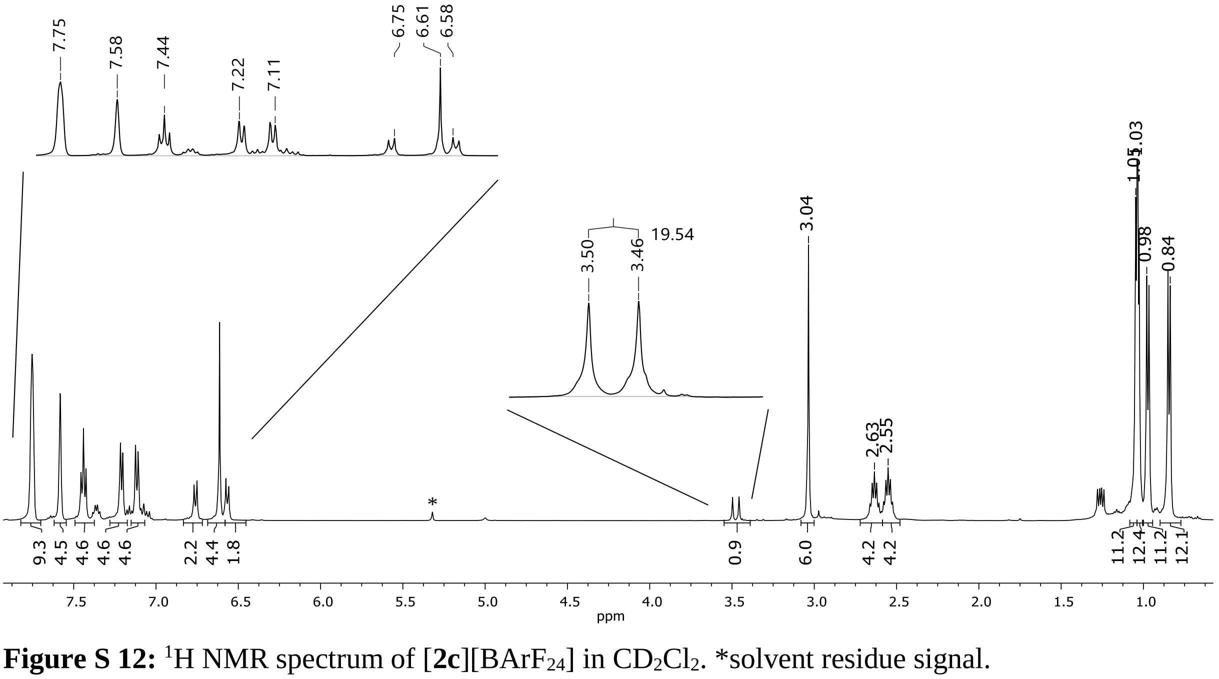

Figure S 12: ^{1}H NMR spectrum of [**2c**][BArF$_{24}$] in CD_2Cl_2. *solvent residue signal.

*

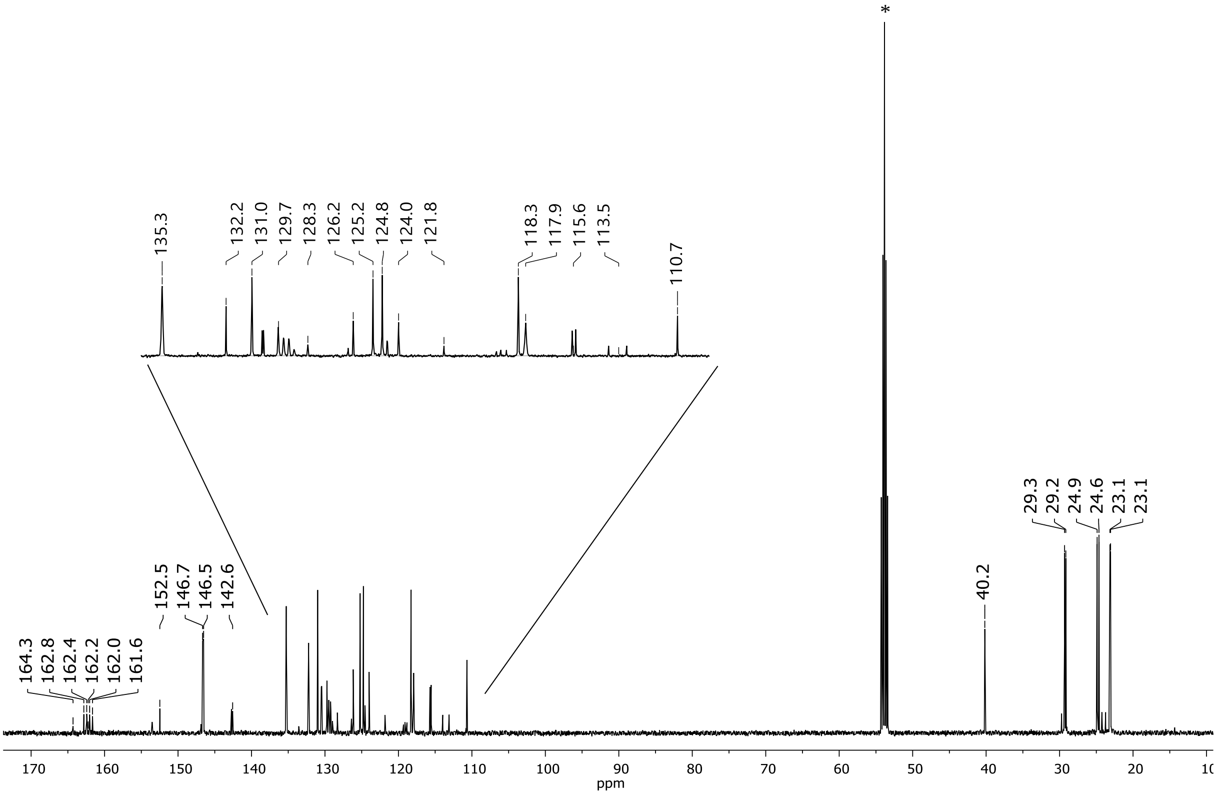

Figure S 13: $^{13}C\{^{1}H\}$ NMR spectrum of [**2c**][$BArF_{24}$] in CD_2Cl_2. *solvent residue signal.

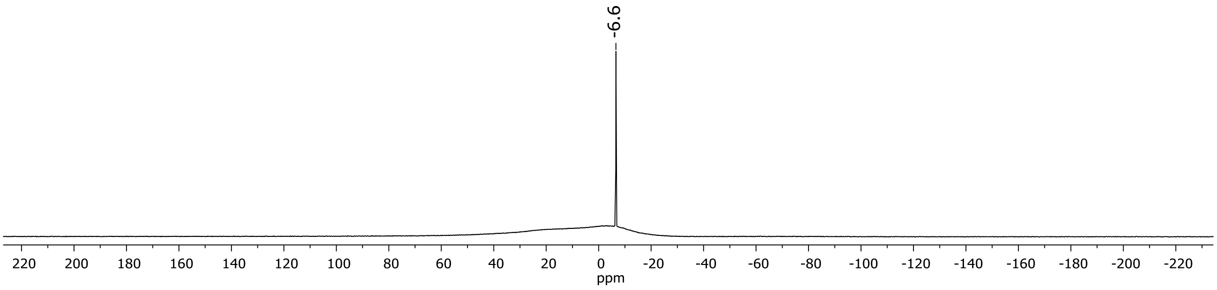

Figure S 14: ^{11}B NMR spectrum of [**2c**][$BArF_{24}$] in CD_2Cl_2.

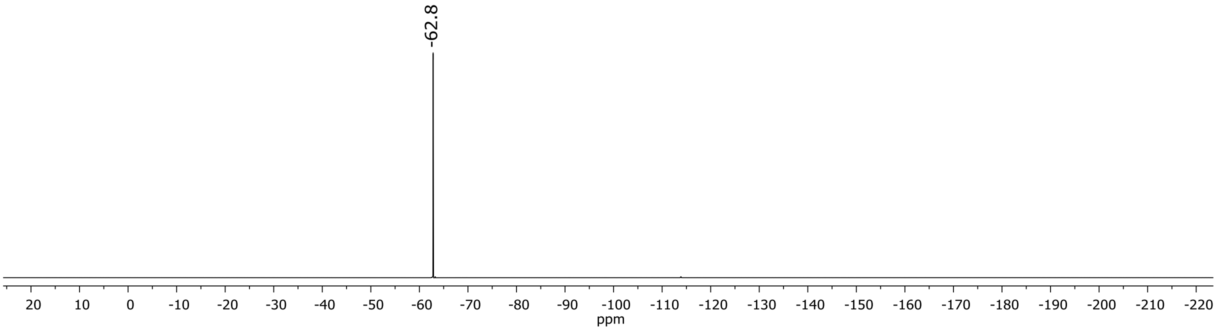

Figure S 15: ^{19}F NMR spectrum of [**2c**][$BArF_{24}$] in CD_2Cl_2.

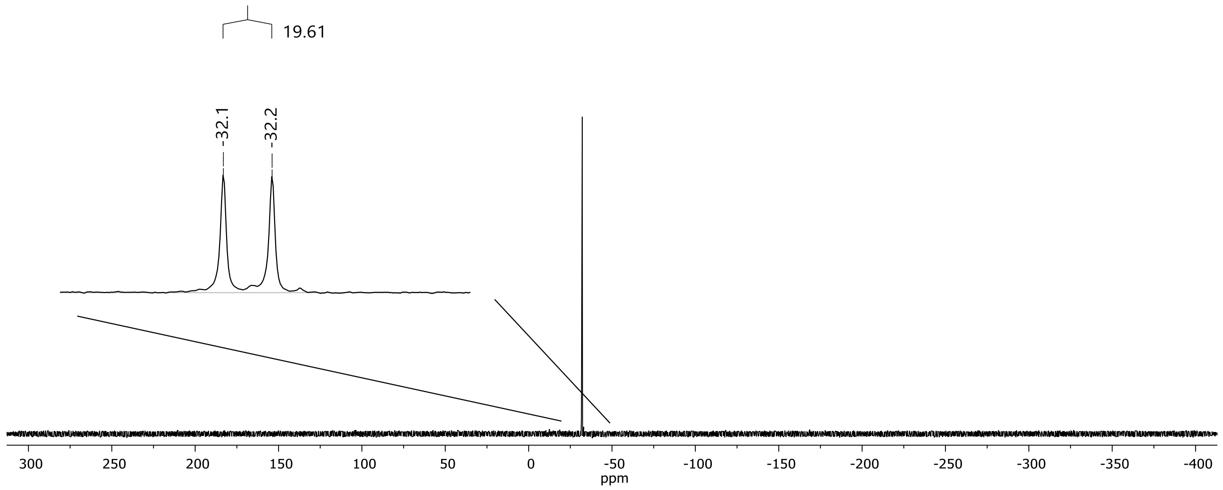

Figure S 16: ^{31}P NMR spectrum of [**2c**][BArF$_{24}$] in CD_2Cl_2.

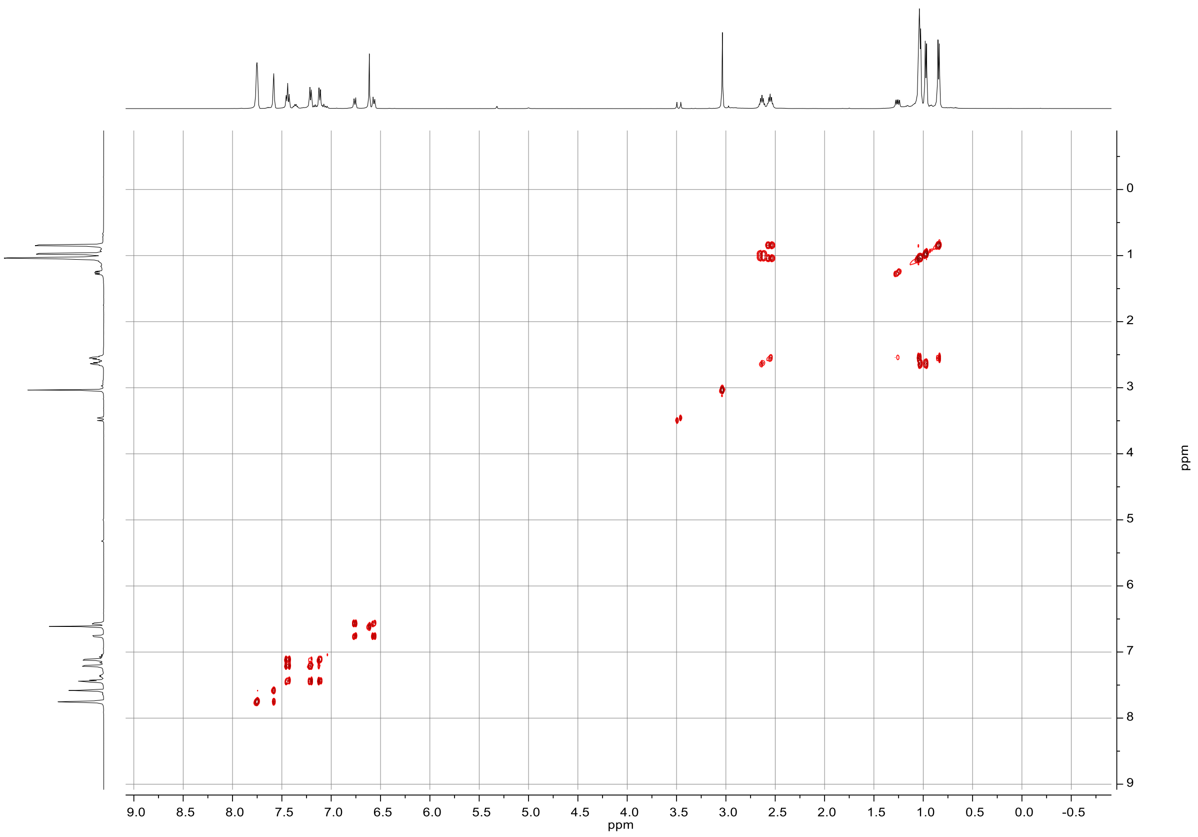

Figure S 17: ^{1}H/^{1}H COSY NMR spectrum of [**2c**][BArF$_{24}$] in CD_2Cl_2.

1.7 **Preparation of [2d][BArF24]**

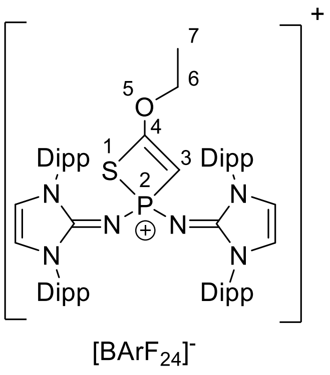

[**1**][BArF24] (0.012 mmol, 20 mg, 1.0 eq.) was dissolved in fluorobenzene and a stock solution of ethoxyacetylene (1.7 wt% in fluorobenzene/hexanes, 0.023 mmol, 94 mg, 2.0 eq.) was added to the solution. After 2 hours, all volatiles were removed *in vacuo*. The product was obtained as a pale-yellow solid .

Remarks: 1) For the synthesis, a commercially availabe stock solution of ethoxyacetylene (~40 wt% in hexanes) was used and diluted with fluorobenzene according to the procedure. 2) The reaction requires one equivalent of ethoxyacetylene. An excess was used owing to the unprecise concentration of the commercially available alkyne and to difficulties in adding small quantities stoichiometrically.

Yield: quantitative.

^{1}H NMR (CD_2Cl_2, 500 MHz, 300 K): δ (ppm) = 7.75 (m, 8 H, BArF24; *ortho*), 7.58 (m, 4 H, BArF24; *para*), 7.44 (t, $^{3}J_{HH}$ = 7.7 Hz, 4 H, *p*-Dipp), 7.19 (dd, $^{3}J_{HH}$ = 7.7 Hz, $^{4}J_{HH}$ = 2.2 Hz, 8 H, *m*-Dipp), 6.66 (s, 4 H, N-C*H*=C*H*-N), 3.34 (q, $^{3}J_{HH}$ = 7.1 Hz, 2 H, 6), 2.28 (d, $^{2}J_{HP}$ = 15.0 Hz, 1 H, 3), 2.59 (m, 8 H, *i*Pr-C*H*), 1.21 (t, $^{3}J_{HH}$ = 7.1 Hz, 2 H, 7) 1.11 (d, $^{3}J_{HH}$ = 6.9 Hz, 12 H, *i*Pr-C*H*3), 1.05 (d, $^{3}J_{HH}$ = 6.9 Hz, 12 H, *i*Pr-C*H*3), 0.97 (d, $^{3}J_{HH}$ = 6.9 Hz, 12 H, *i*Pr-C*H*3), 0.94 (d, $^{3}J_{HH}$ = 6.9 Hz, 12 H, *i*Pr-C*H*3).

^{13}C{^{1}H} NMR (CD_2Cl_2, 126 MHz, 300 K): δ (ppm) = 164.3 (d, $^{2}J_{CP}$ = 13 Hz, 4), 162.2 (q, $^{1}J_{CB}$ = 50 Hz, BArF24; ipso), 146.7 (*o*-Dipp), 146.6 (*o*-Dipp), 142.8 (d, $^{2}J_{CP}$ = 20 Hz, N-*C*-N), 135.3 (BArF24; *ortho*), 132.2 (*i*-Dipp), 131.0 (*p*-Dipp), 129.3 (qq, $^{2}J_{CF}$ = 32 Hz, $^{4}J_{CF}$ = 3 Hz, BArF24; *meta*), 125.1 (q, $^{1}J_{CF}$ = 272 Hz, BArF24; *C*F3), 125.0 (*m*-Dipp), 124.9 (*m*-Dipp), 118.4 (N-*C*H=*C*H-N), 117.9 (sept, $^{3}J_{CF}$ = 4 Hz, BArF24; *para*), 69.1 (6); 93.4 (d, $^{1}J_{CP}$ = 118 Hz, 3), 29.3 (*i*Pr-*C*H), 29.2 (*i*Pr-*C*H), 24.9 (*i*Pr-*C*H3), 24.9 (*i*Pr-*C*H3), 23.1 (*i*Pr-*C*H3), 23.0 (*i*Pr-*C*H3), 14.0 (7).

^{11}B NMR (CD_2Cl_2, 160 MHz, 300 K): δ (ppm) = -6.6.

^{19}F NMR (CD_2Cl_2, 471 MHz, 300 K): δ (ppm) = -62.8.

^{31}P NMR (CD_2Cl_2, 202 MHz, 300 K): δ (ppm) = -35.7 (d, $^{2}J_{PH}$ = 15 Hz).

HR-MS(ESI): m/z was calculated for $[C_{58}H_{78}N_6OPS]^+$ as [**2d**]$^+$: 937.56899 found as: 937.56868. Fitting isotope pattern: Yes.

Single crystal X-ray diffraction analysis: Single crystals suitable for X-ray diffraction analysis were obtained by cooling down a saturated solution of [**2d**][BArF24] in CH_2Cl_2. A molecular structure was obtained.

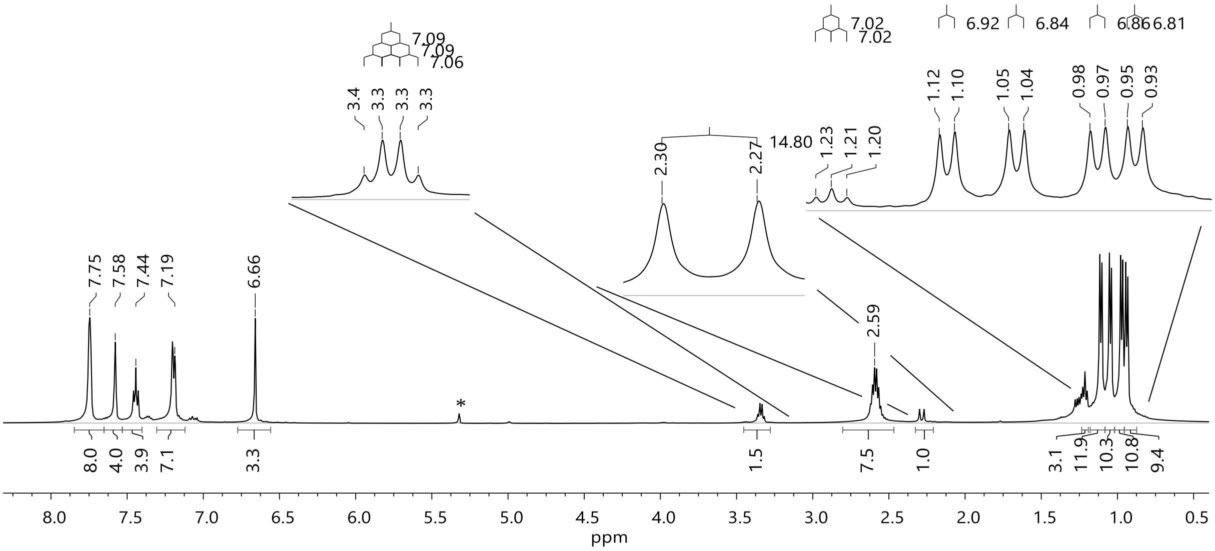

Figure S 18: ^{1}H NMR spectrum of [**2d**][BArF$_{24}$] in CD_2Cl_2. *solvent residue signal.

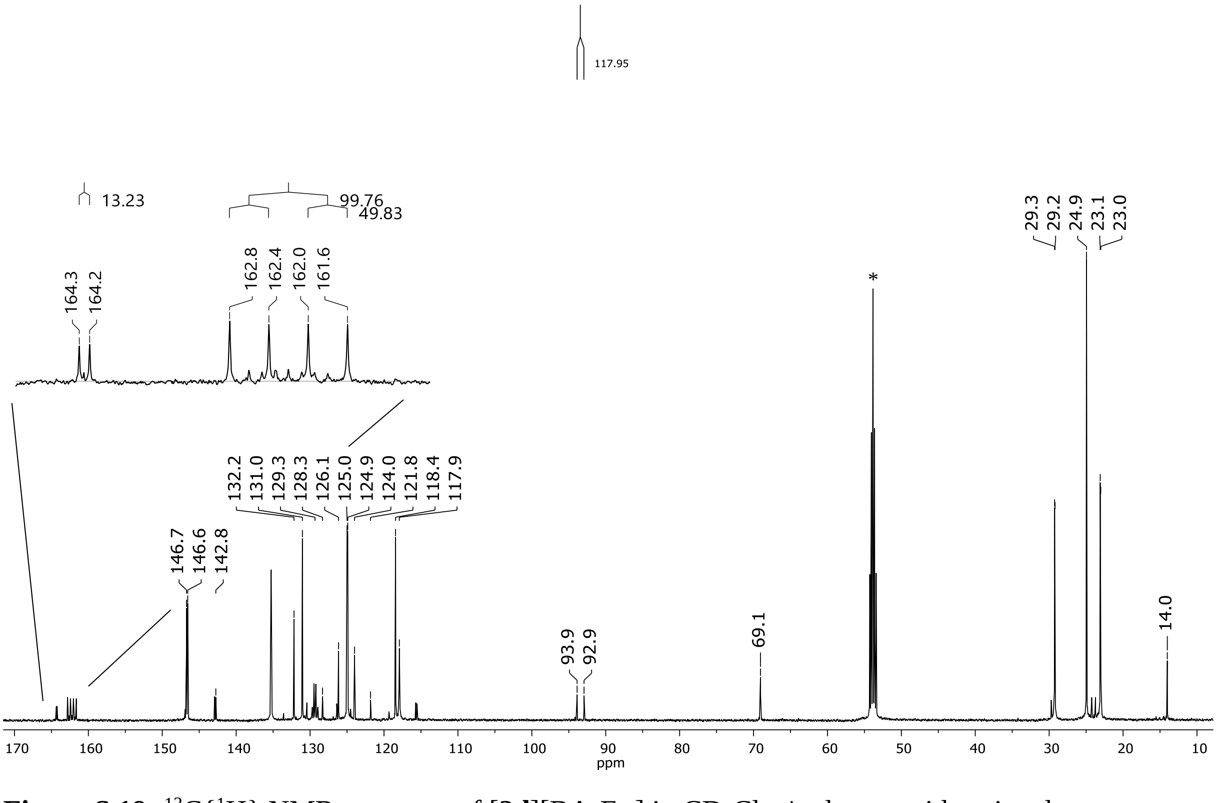

Figure S 19: $^{13}C\{^{1}H\}$ NMR spectrum of [**2d**][BArF$_{24}$] in CD_2Cl_2. *solvent residue signal.

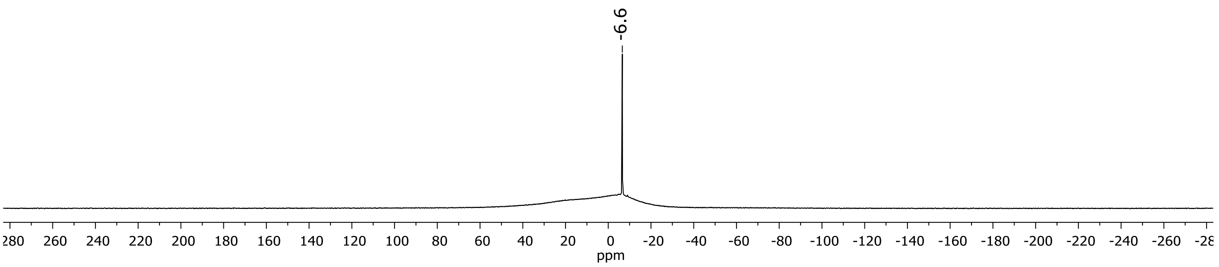

Figure S 20: ^{11}B- NMR spectrum of [**2d**][$BArF_{24}$] in CD_2Cl_2.

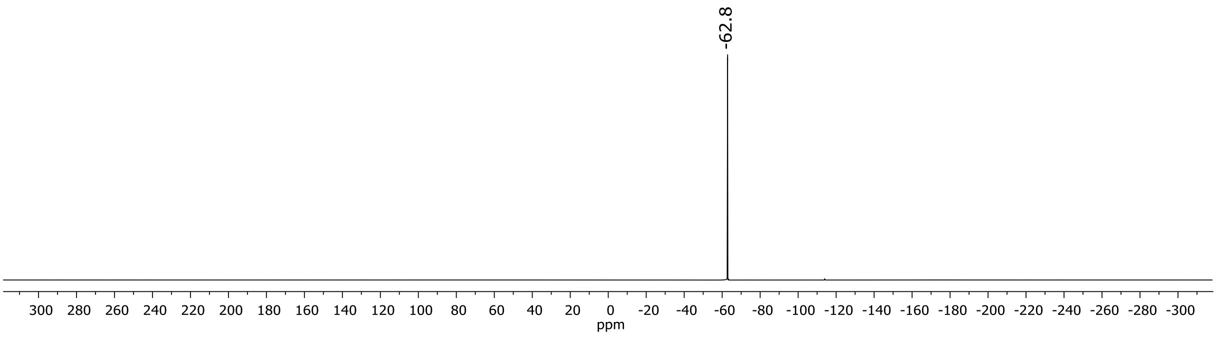

Figure S 21: ^{19}F NMR spectrum of [**2d**][$BArF_{24}$] in CD_2Cl_2.

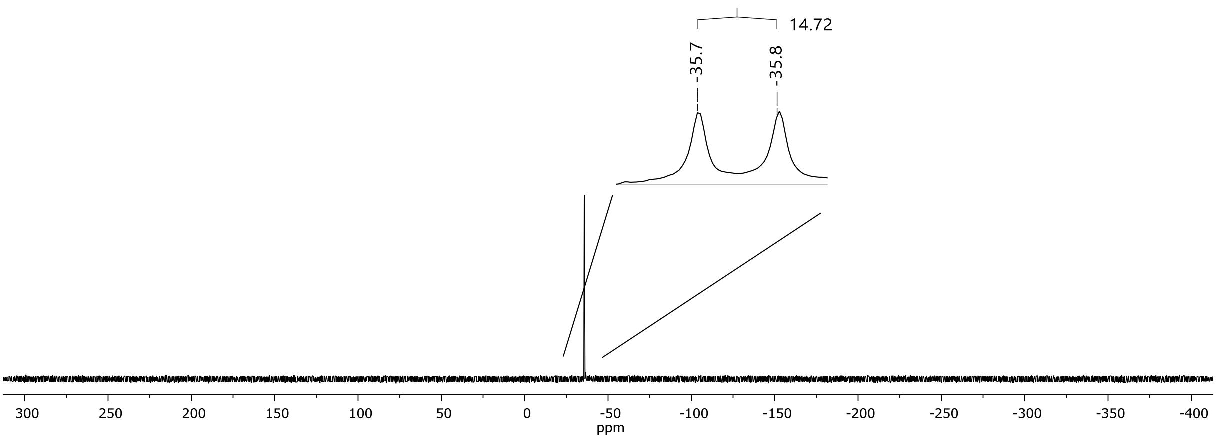

Figure S 22: ^{31}P NMR spectrum of [**2d**][$BArF_{24}$] in CD_2Cl_2.

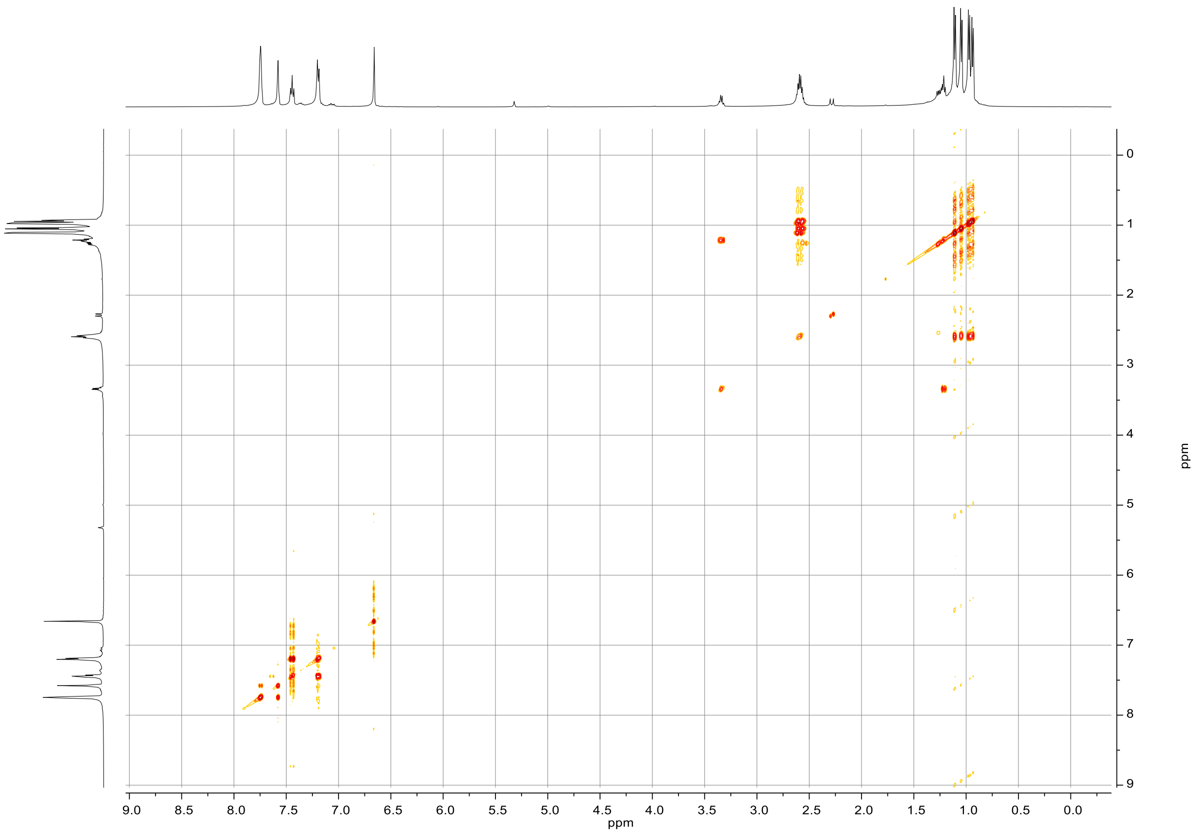

Figure S 23: $^{1}H/^{1}H$ COSY NMR spectrum of [**2d**][BArF$_{24}$] in CD_2Cl_2.

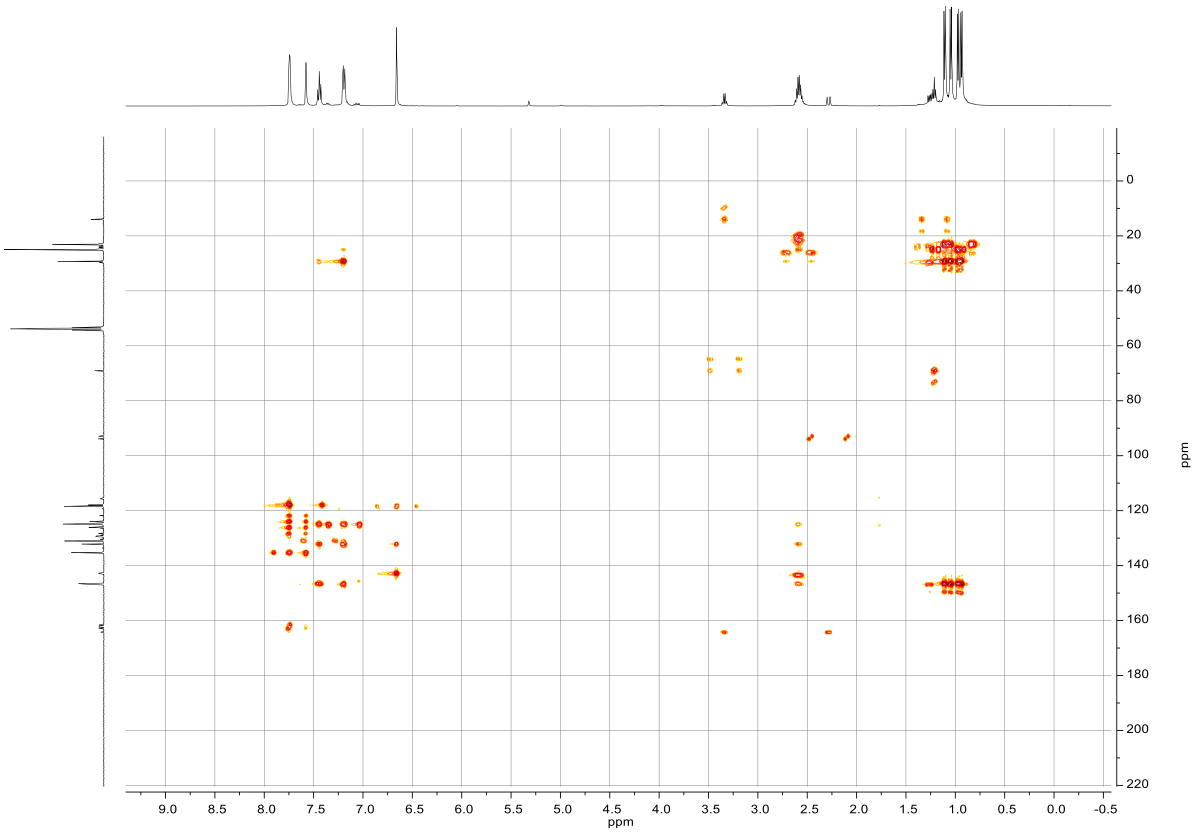

Figure S 24: $^{1}H/^{13}C$ HMBC NMR spectrum of [**2d**][BArF$_{24}$] in CD_2Cl_2.

1.8 Preparation of [2e][BArF$_{24}$]

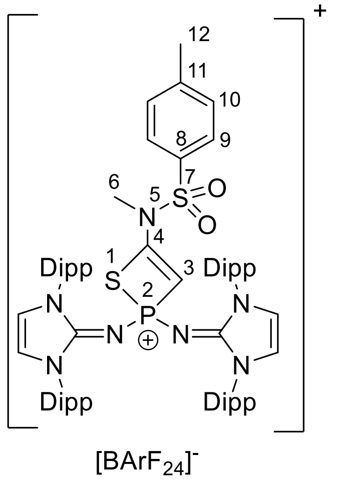

[**1**][BArF$_{24}$] (0.058 mmol, 100 mg, 1.0 eq.) was dissolved in dichloromethane and N-ethynyl-N,4-dimethylbenzenesulfonamide (0.058mmol, 12 mg, 1.0 eq.) was added to the solution. After 2 hours, all volatiles were removed *in vacuo*. The product was obtained as a pale red solid.

NMR-yield: 97 %.

^{1}H NMR (CD_2Cl_2, 500 MHz, 300 K): δ (ppm) = 7.77 (m, 8 H, BArF$_{24}$; *ortho*), 7.64 (m, 2 H, 9), 7.60 (m, 4 H, BArF$_{24}$; *para*), 7.47 (t, $^{3}J_{HH}$ = 7.8 Hz, 4 H, *p*-Dipp), 7.37 (m, 2 H, 10), 7.21 (m, 4H, *m*-Dipp), 7.20 (m, 4 H, *m*-Dipp), 6.66 (s, 4 H, N-C*H*=C*H*-N), 2.70 (s, 3 H, 6), 2.70 (m, 2 H, *i*Pr-C*H*), 2.58 (m, 2 H, *i*Pr-C*H*), 2.55 (m, 1 H, 3), 2.46 (s, 3 H, 12), 1.06 (m, 12 H, *i*Pr-C*H*$_3$), 1.06 (m, 12 H, *i*Pr-C*H*$_3$), 0.94 (d, $^{3}J_{HH}$ = 6.9 Hz, 12 H, *i*Pr-C*H*$_3$), 0.82 (d, $^{3}J_{HH}$ = 6.9 Hz, 12 H, *i*Pr-C*H*$_3$).

^{13}C{^{1}H} NMR (CD_2Cl_2, 126 MHz, 300 K): δ (ppm) = 162.3 (q, $^{1}J_{CB}$ = 50 Hz, BArF$_{24}$; ipso), 146.5 (*o*-Dipp), 146.4 (*o*-Dipp), 146.4 (11), 142.0 (d, $^{2}J_{CP}$ = 21 Hz, N-*C*-N), 135.6 (8), 135.3 (BArF$_{24}$; *ortho*), 132.2 (*i*-Dipp), 131.2 (*p*-Dipp), 130.6 (10), 129.4 (qq, $^{2}J_{CF}$ = 32 Hz, $^{4}J_{CF}$ = 3 Hz, BArF$_{24}$; *meta*), 128.2 (9), 125.2 (q, $^{1}J_{CF}$ = 274 Hz, BArF$_{24}$; *C*F$_3$), 125.3 (*m*-Dipp), 125.2 (*m*-Dipp), 118.5 (N-*C*H=*C*H-N), 118.0 (m, BArF$_{24}$; *para*), 101.0 (d, $^{1}J_{CP}$ = 115 Hz, 3), 36.3 (6), 29.3 (*i*Pr-*C*H), 29.2 (*i*Pr-*C*H), 24.7 (*i*Pr-*C*H$_3$), 24.4 (*i*Pr-*C*H$_3$), 23.1 (*i*Pr-*C*H$_3$), 22.9 (*i*Pr-*C*H$_3$), 21.8 (12).

^{11}B NMR (CD_2Cl_2, 160 MHz, 300 K): δ (ppm) = -6.6.

^{19}F NMR (CD_2Cl_2, 471 MHz, 300 K): δ (ppm) = -62.8.

^{31}P NMR (CD_2Cl_2, 202 MHz, 300 K): δ (ppm) = -36.4 (d, $^{2}J_{PH}$ = 15 Hz).

HR-MS(ESI): m/z was calculated for $[C_{64}H_{93}N_7O_2PS_2]^+$ as [**2e**]$^+$: 1076.57818 found as: 1076.57843. Fitting isotope pattern: Yes.

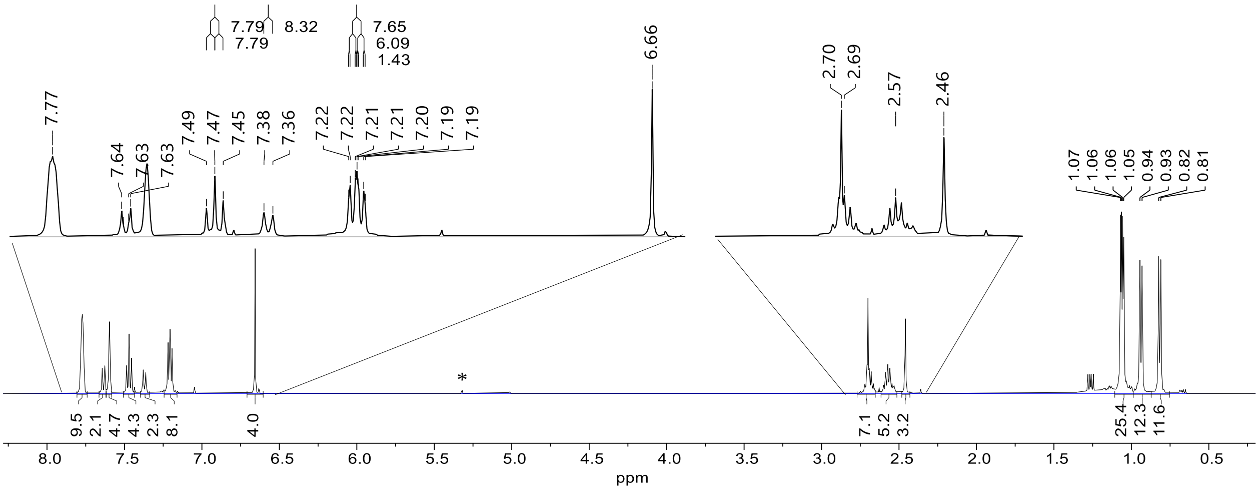

Figure S 25: ^{1}H NMR spectrum of [**2e**][BArF$_{24}$] in CD_2Cl_2. *solvent residue signal.

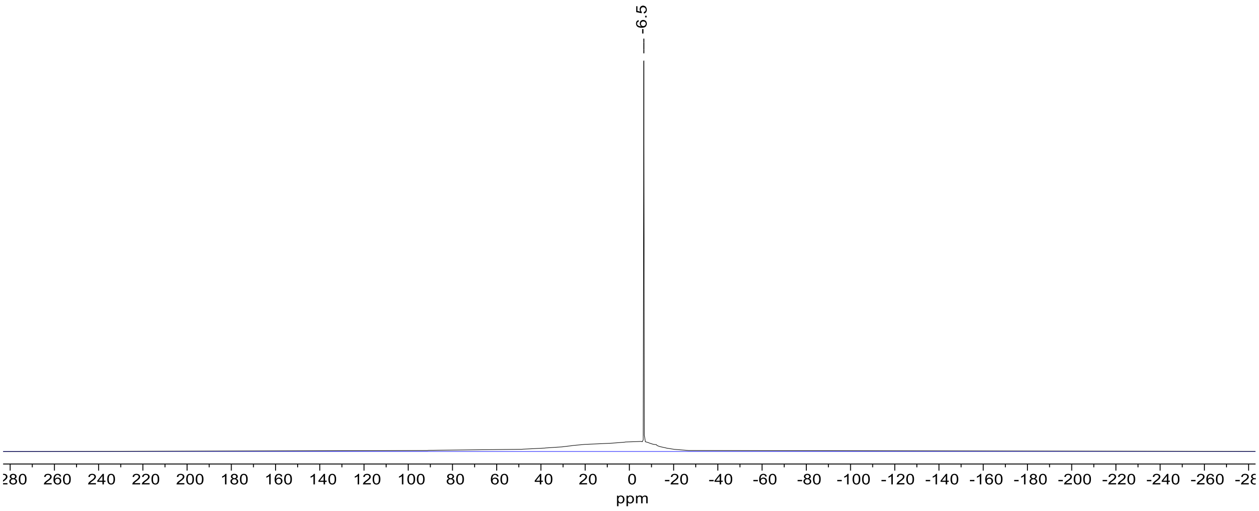

Figure S 26: ^{11}B NMR spectrum of [**2e**][BArF$_{24}$] in CD$_2$Cl$_2$.

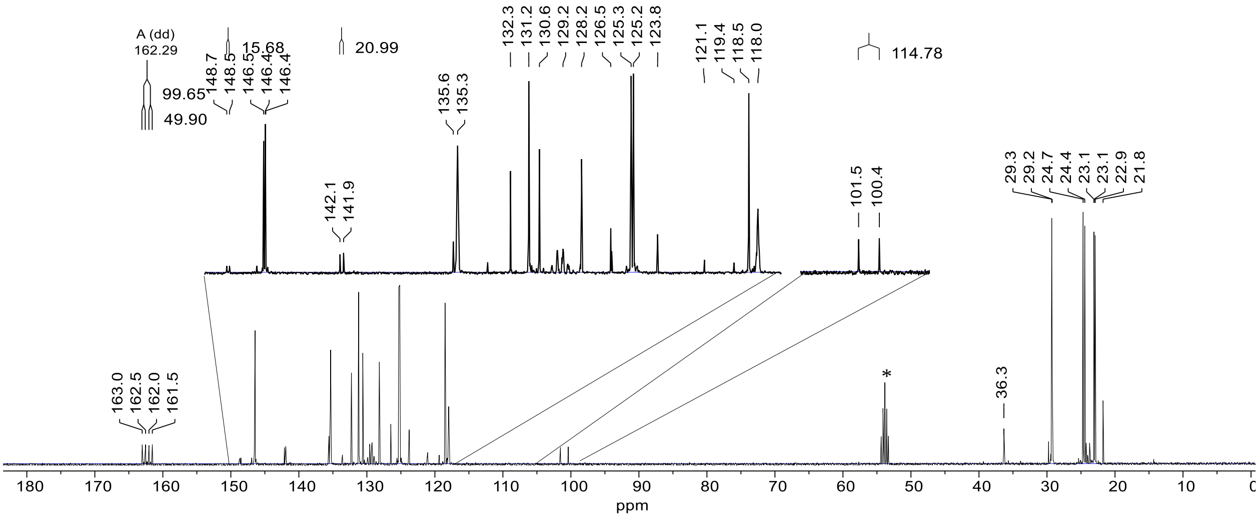

Figure S 27: ^{13}C{^{1}H} NMR spectrum of [**2e**][BArF$_{24}$] in CD$_2$Cl$_2$. *solvent residue signal.

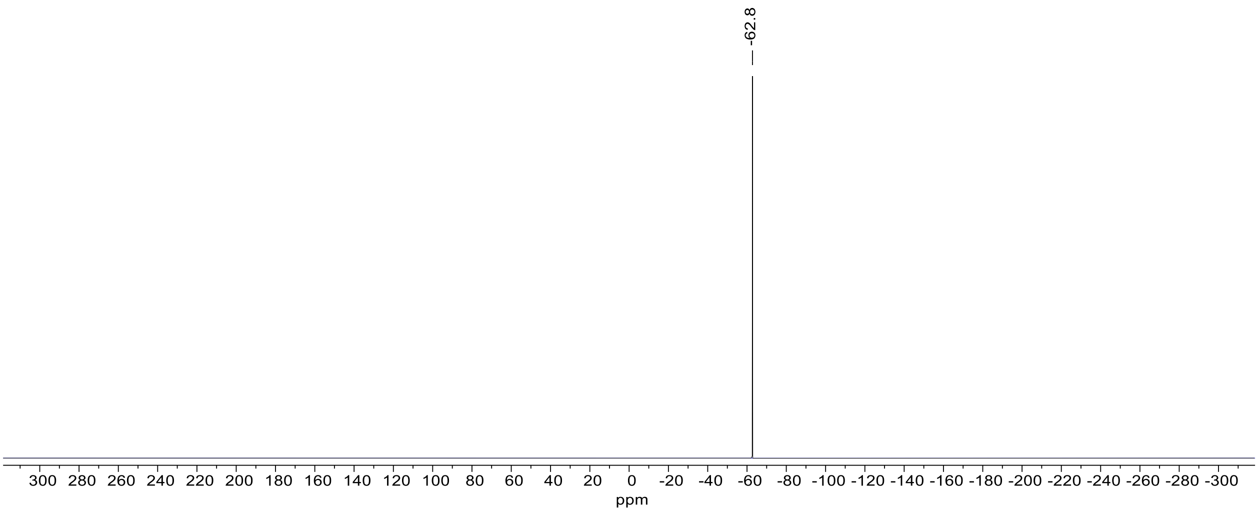

Figure S 28: ^{19}F NMR spectrum of [**2e**][BArF$_{24}$] in CD$_2$Cl$_2$.

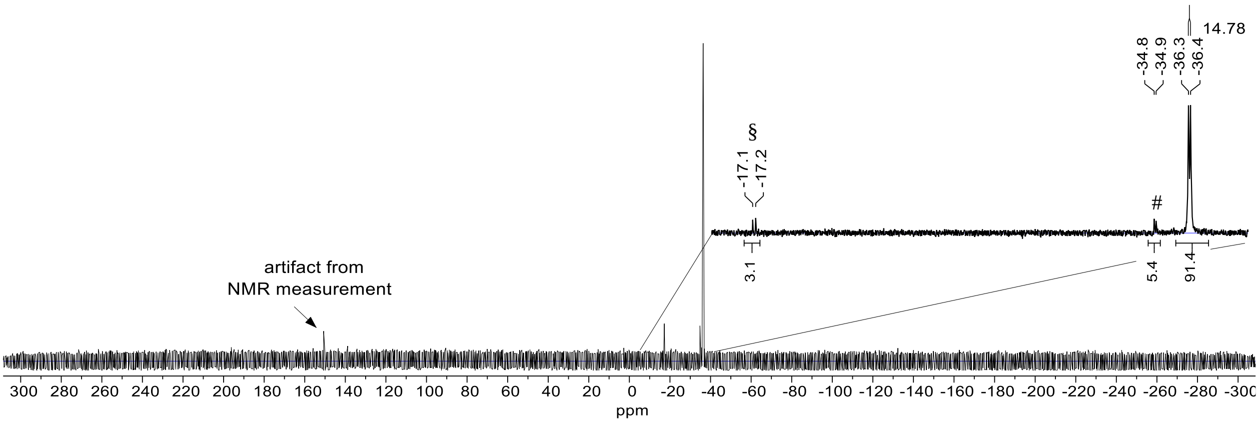

Figure S 29: ^{31}P NMR spectrum of [**2e**][BArF$_{24}$] in CD_2Cl_2. §unidentified impurity. #structural isomer within the Dipp-group.

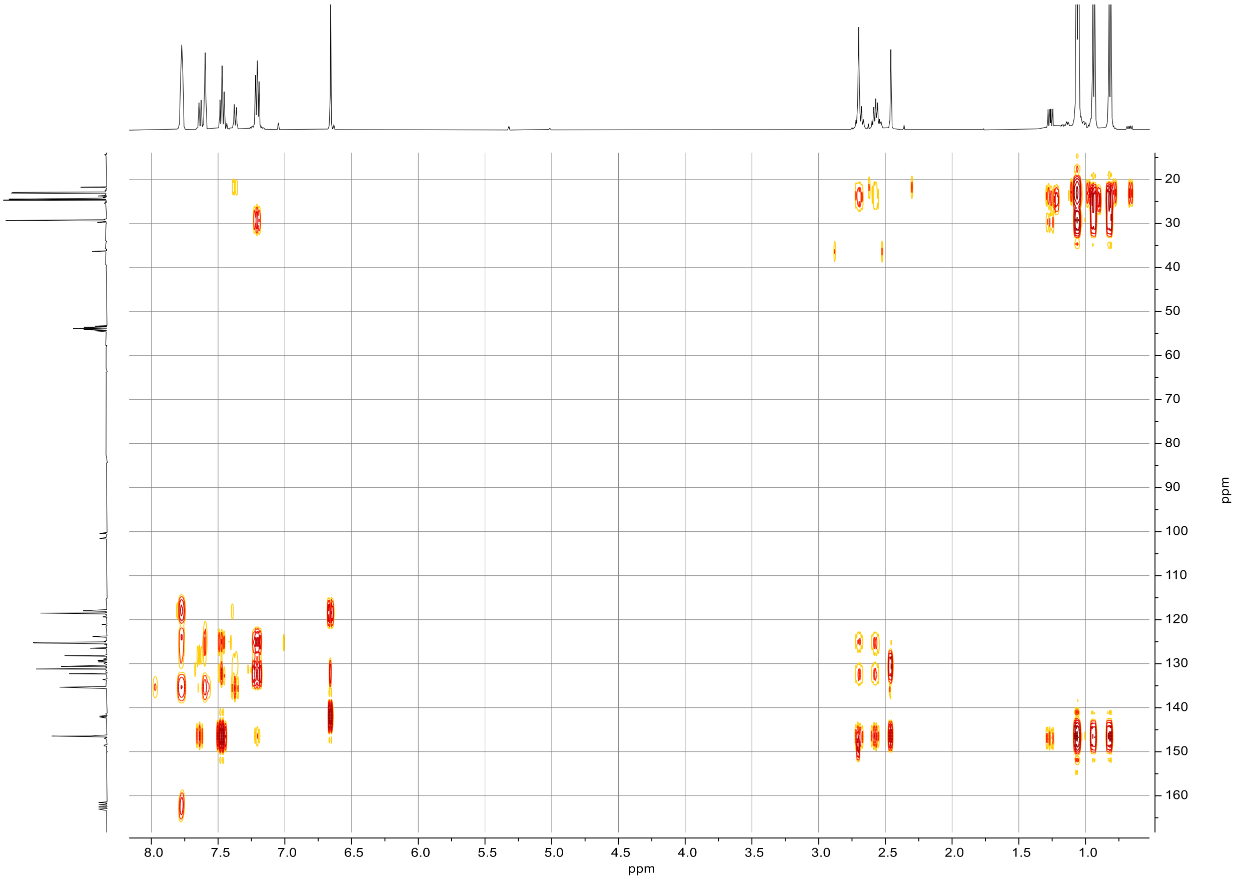

Figure S 30: ^{1}H\$^{13}$C HMBC spectrum of [**2e**][BArF$_{24}$] in CD_2Cl_2.

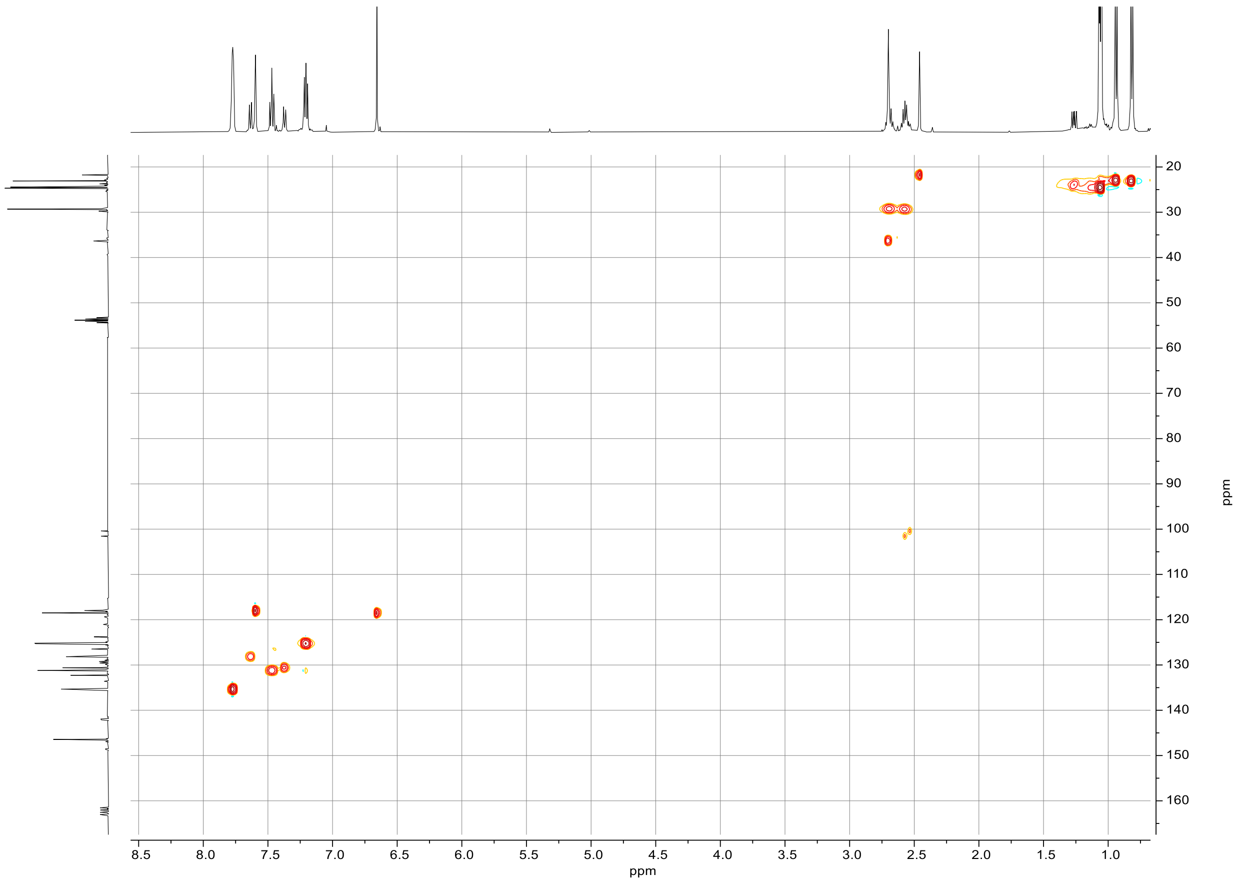

Figure S 31: $^{1}H\backslash^{13}C$ HSQC spectrum of [**2e**][$BArF_{24}$] in CD_2Cl_2.

1.9 Preparation of [2f][BArF24]

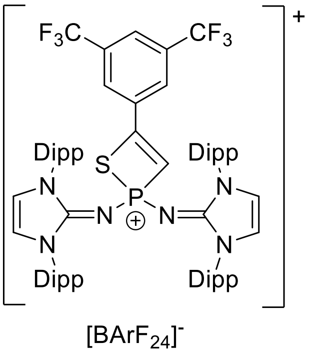

[**1**][BArF24] (0.012 mmol, 20 mg, 1.0 eq.) was dissolved in fluorobenzene and a large excess (one drop) of 1-Ethynyl-3,5-bis(trifluoromethyl)benzene was added to the mixture. The solution was subsequently heated to 120 °C, 140 °C and 180 °C for 16 h at each temperature level.

Note: The reaction requires one equivalent of 1-ethynyl-3,5-bis(trifluoromethyl)benzene. An excess was used owing to difficulties in adding small quantities stoichiometrically.

After heating at 120 °C for 16 h and 140 °C for 16 h, no conversion was observed. After heating at 180 °C for 16 h, 12 % conversion according to ^{31}P NMR was observed.

^{31}P NMR (162 MHz, C_6H_5F): δ (ppm) = -40.6 (d, $^2J_{PH}$= 18 Hz).

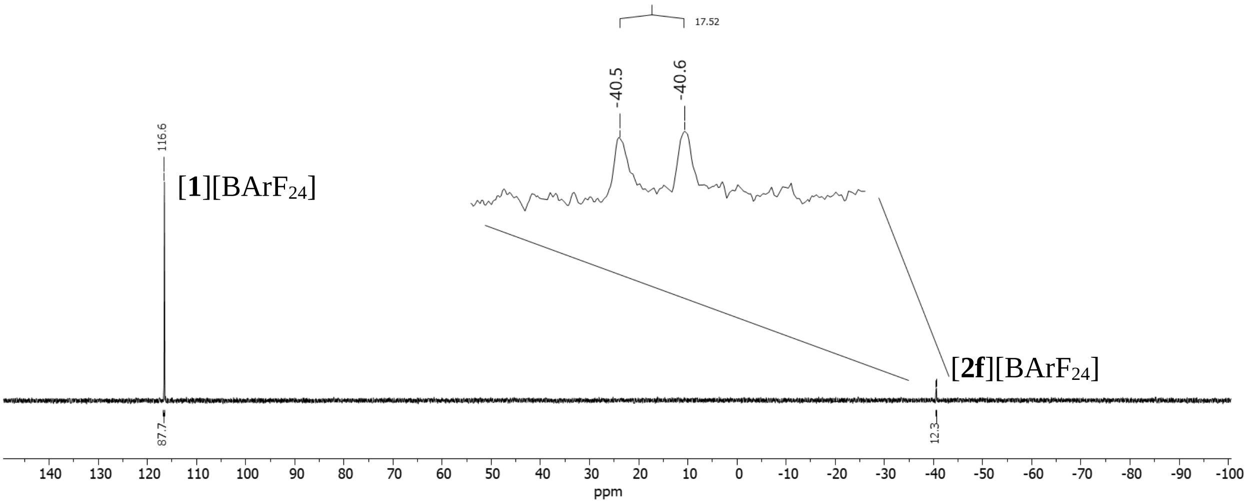

Figure S 32: ^{31}P NMR spectrum of the reaction of [**1**][BArF24] and 1-Ethynyl-3,5-bis(trifluoromethyl)benzene in fluorobenzene after heating at 180 °C for 16 h.

1.10 Preparation of [3a][BArF24]

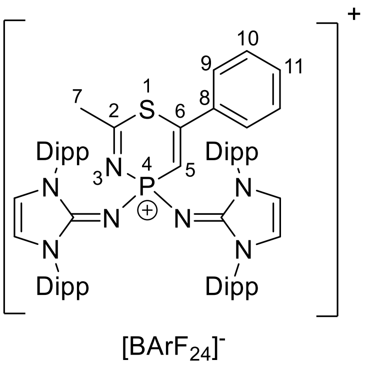

[**2a**][BArF$_{24}$] (0.0818 mmol, 150 mg) was suspended in acetonitrile (1 mL). The mixture was heated at 170 °C for 16 h. While letting the solution cool to room temperature, crystals of [**3a**][BArF$_{24}$] precipitate from the solution. All volatiles were removed *in vacuo.* The product was obtained as a beige solid.

Yield: quantitative.

Remark: Although the reaction cleanly affords [**3a**][BArF$_{24}$], the crystalline [**3a**][BArF$_{24}$] may also be directly isolated by pipetting of the mother liquor and drying the beige crystalline needles *in vacuo* (72 mg, 48% isolated yield).

^{1}H NMR (CD_2Cl_2, 500 MHz, 300 K): δ (ppm) = 7.75 (m, 8 H, BArF$_{24}$; *ortho*), 7.58 (m, 4 H, BArF$_{24}$; *para*), 7.46 (m, 1 H, 11), 7.40 (m, 2 H, 10), 7.32 (t, $^{3}J_{HH}$ = 7.8 Hz, 4 H, *p*-Dipp), 7.15 (d, $^{3}J_{HH}$ = 7.8 Hz, 4 H, *m*-Dipp), 7.13 (d, $^{3}J_{HH}$ = 7.8 Hz, 4 H, *m*-Dipp), 7.07 (m, 2 H, 9), 6.47 (s, 4 H, N-C*H*=C*H*-N), 5.44 (d, $^{2}J_{HP}$ = 8.0 Hz, 1H, 5), 2.63 (m, 4 H, *i*Pr-C*H*), 2.56 (m, 4 H, *i*Pr-C*H*), 1.58 (s, 3H, 7), 1.06 (d, $^{3}J_{HH}$ = 6.9 Hz, 12 H, *i*Pr-C*H*$_3$), 1.03 (d, $^{3}J_{HH}$ = 6.9 Hz, 12 H, *i*Pr-C*H*$_3$), 1.01 (d, $^{3}J_{HH}$ = 6.9 Hz, 12 H, *i*Pr-C*H*$_3$), 0.95 (d, $^{3}J_{HH}$ = 6.9 Hz, 12 H, *i*Pr-C*H*$_3$).

^{13}C{^{1}H} NMR (CD_2Cl_2, 126 MHz, 300 K): δ (ppm) = 168.7 (d, $^{2}J_{CP}$ = 5 Hz, 2), 162.3 (q, $^{1}J_{CB}$ = 50 Hz, BArF$_{24}$; ipso), 147.5 (d, $^{2}J_{CP}$ = 6 Hz, 6), 146.5 (*o*-Dipp), 146.3 (*o*-Dipp), 142.3 (d, $^{2}J_{CP}$ = 30 Hz, N-C-N), 137.2 (d, $^{2}J_{CP}$ = 13 Hz, 8), 135.3 (BArF$_{24}$; *ortho*), 132.7 (*i*-Dipp), 130.9 (*p*-Dipp), 129.4 (qq, $^{2}J_{CF}$ = 32 Hz, $^{4}J_{CF}$ = 3 Hz, BArF$_{24}$; *meta*), 125.3 (*m*-Dipp), 125.0 (*m*-Dipp), 125.1 (q, $^{1}J_{CF}$ = 272 Hz, BArF$_{24}$; *C*F$_3$), 118.3 (N-*C*H=*C*H-N), 117.9 (sept, $^{3}J_{CF}$ = 4 Hz, BArF$_{24}$; *para*), 110.3 (d, $^{2}J_{CP}$ = 141 Hz, 5), 32.0 (d, $^{3}J_{CP}$ = 15 Hz, 7), 29.4 (*i*Pr-*C*H), 29.3 (*i*Pr-*C*H), 24.7 (*i*Pr-*C*H$_3$), 24.4 (*i*Pr-*C*H$_3$), 23.7 (*i*Pr-*C*H$_3$), 23.1 (*i*Pr-*C*H$_3$).

^{11}B NMR (CD_2Cl_2, 160 MHz, 300 K): δ (ppm) = -6.6.

^{19}F NMR (CD_2Cl_2, 471 MHz, 300 K): δ (ppm) = -62.8.

^{31}P NMR (CD_2Cl_2, 162 MHz, 300 K): δ (ppm) = -34.0 (d, $^{2}J_{PH}$ = 8 Hz).

HR-MS(ESI): m/z was calculated for $[C_{60}H_{81}N_7OPS]^+$ as [**3a**]$^+$: 1010.60063 found was: 1010.59982. Fitting isotope pattern: Yes.

Single crystal X-ray diffraction analysis: Single crystals suitable for X-ray diffraction analysis were obtained while cooling down the reaction mixture (*vide supra*).

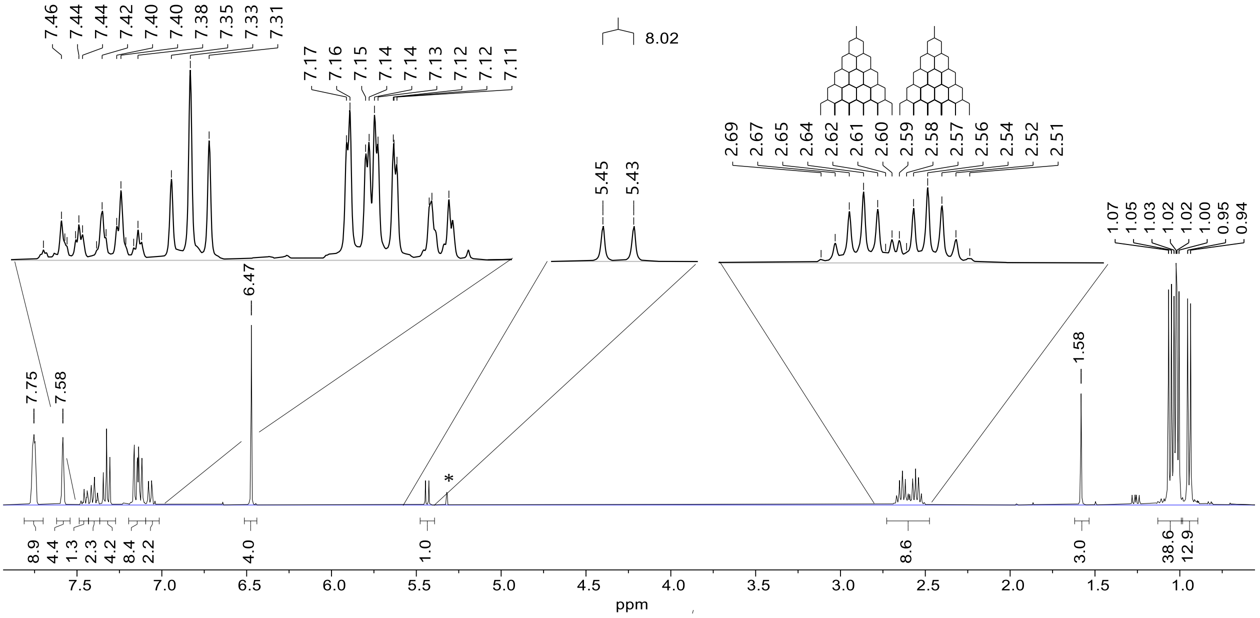

Figure S 33: ^{1}H NMR spectrum of [**3a**][BArF$_{24}$] in CD_2Cl_2. *solvent residue signal.

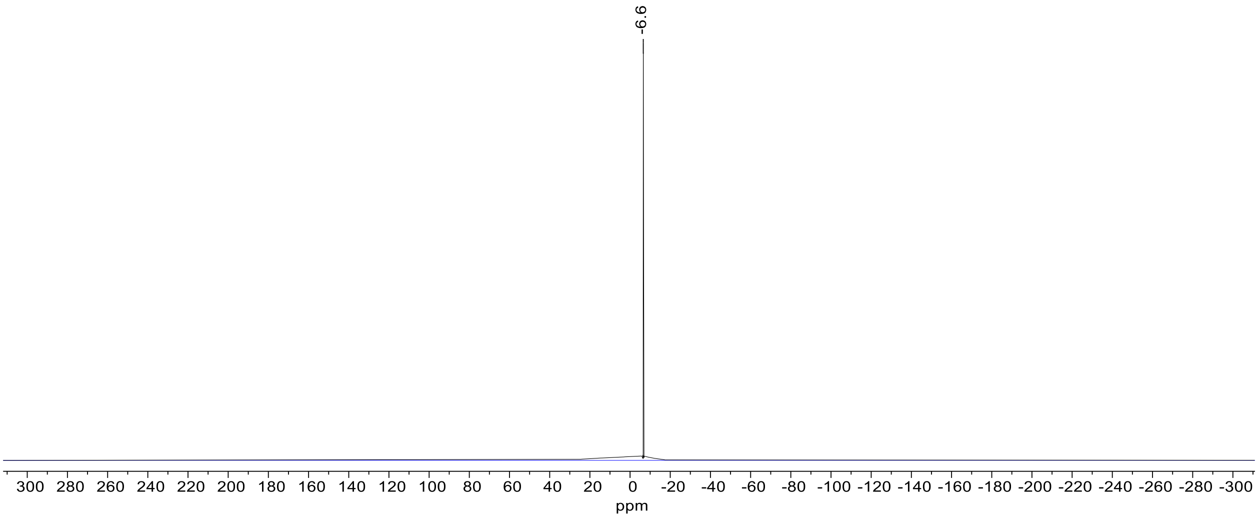

Figure S 34: $^{11}B\{H\}$ NMR spectrum of [**3a**][BArF$_{24}$] in CD_2Cl_2.

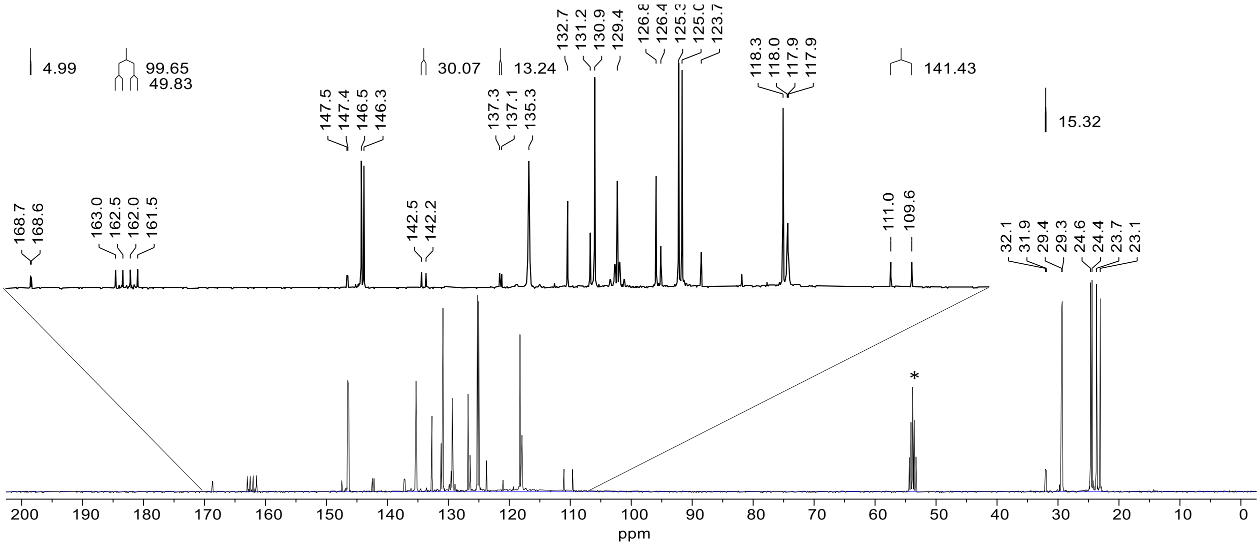

Figure S 35: $^{13}C\{^{1}H\}$ NMR spectrum of [**3a**][$BArF_{24}$] in CD_2Cl_2. *solvent residue signal.

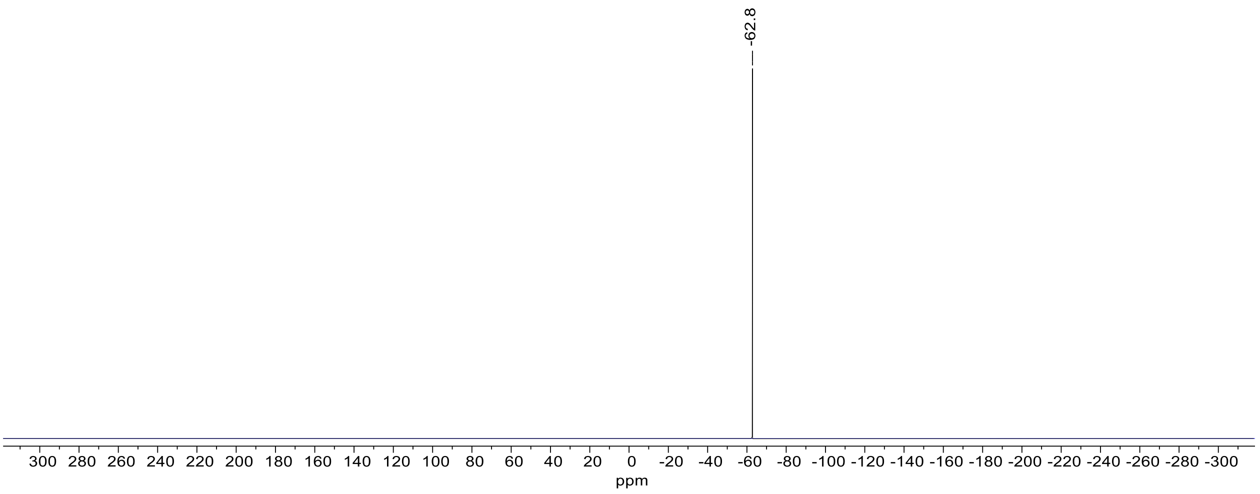

Figure S 36: ^{19}F NMR spectrum of [**3a**][$BArF_{24}$] in CD_2Cl_2.

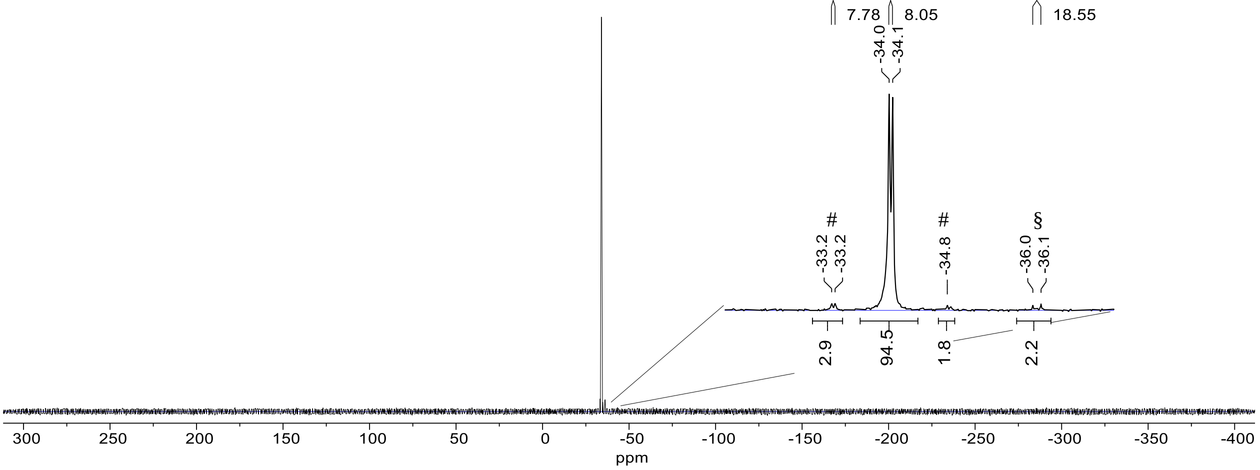

Figure S 37: ^{31}P NMR spectrum of [**3a**][$BArF_{24}$] in CD_2Cl_2. §unidentified impurity. #structural isomers within the Dipp-group.

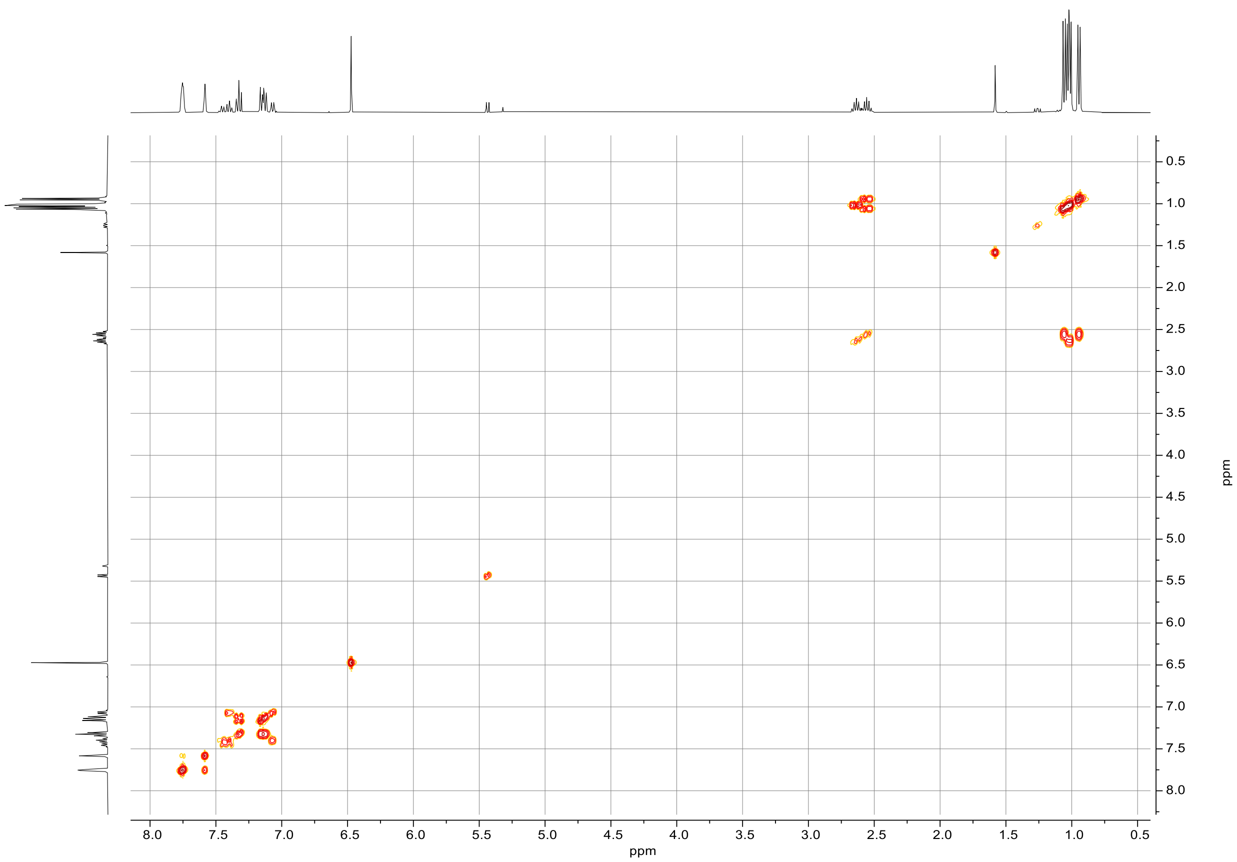

Figure S 38: $^1H/^1H$ COSY spectrum of [**3a**][BArF$_{24}$] in CD_2Cl_2.

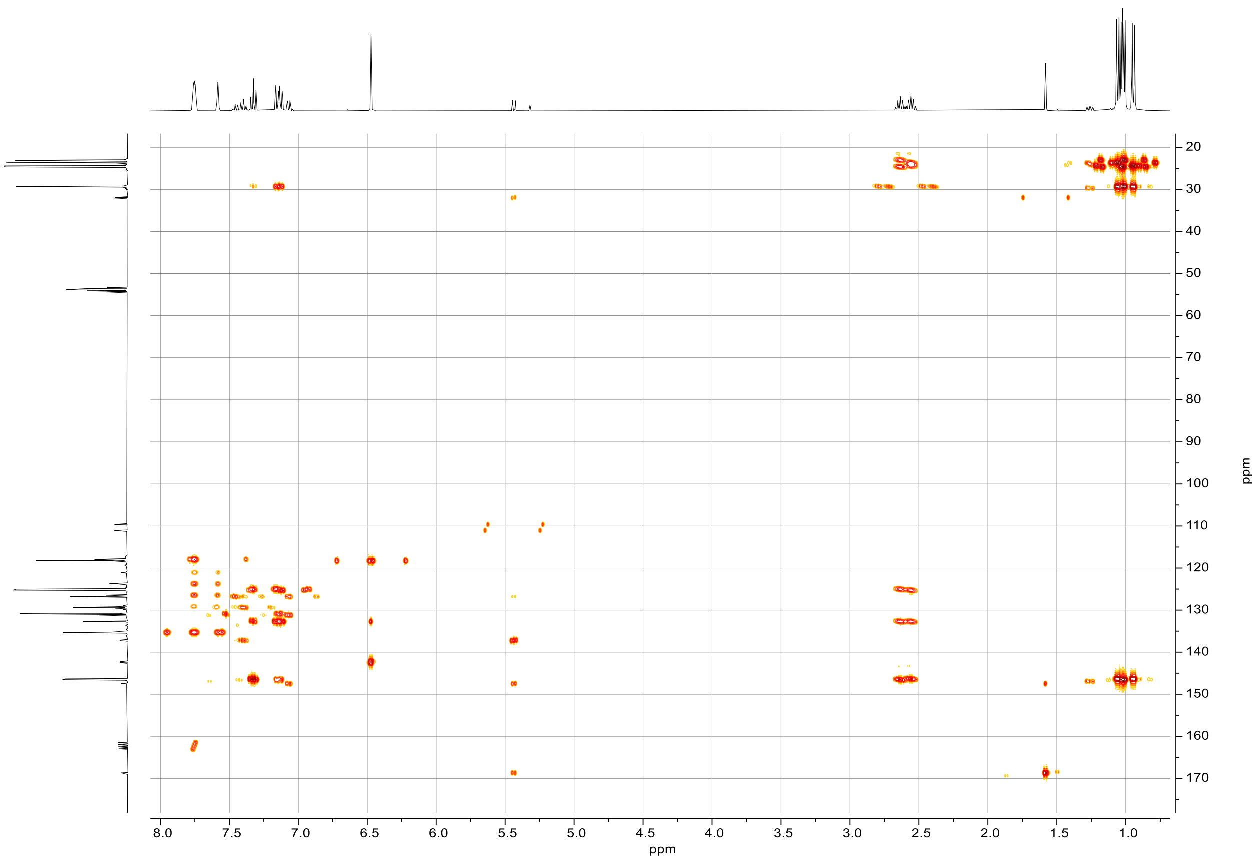

Figure S 39: $^1H\backslash^{13}C$ HMBC spectrum of [**3a**][BArF$_{24}$] in CD_2Cl_2.

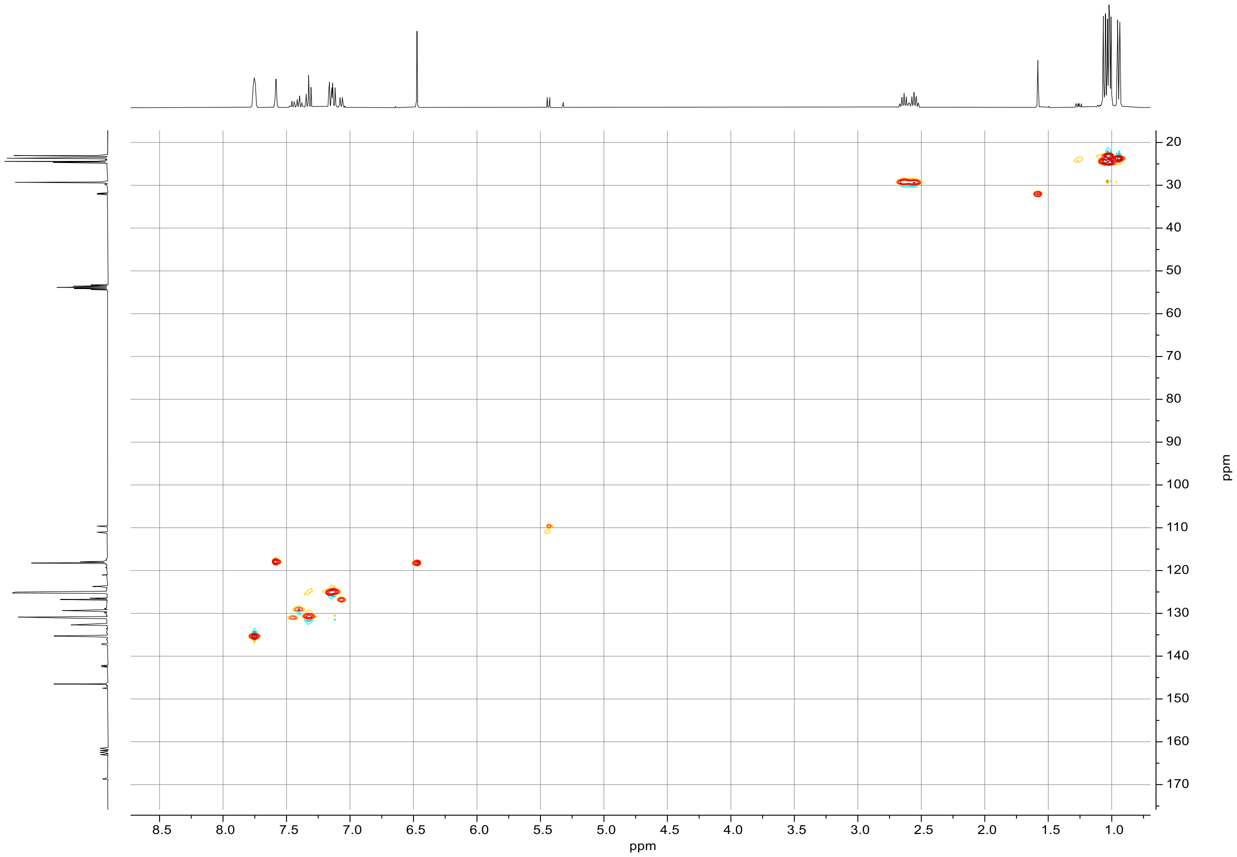

Figure S 40: $^{1}H\backslash^{13}C$ HSQC spectrum of [**3a**][$BArF_{24}$] in CD_2Cl_2.

1.11 Preparation of [3d][$BArF_{24}$]

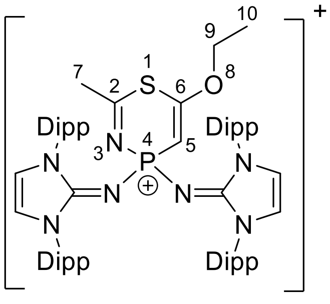

[$BArF_{24}$]$^-$

[**2d**][$BArF_{24}$] (0.0555 mmol, 100 mg) was dissolved in fluorobenzene (0.5 mL) and a large excess of acetonitrile (3 mL) was added. The mixture was left at room temperature overnight in a sealed vessel, which resulted in the formation of colourless crystals. The mother liquor was pipetted off and discarded. The crystals were dried *in vacuo*.

Yield: quantitative.

^{1}H NMR (CD_2Cl_2, 500 MHz, 300 K): δ (ppm) = 7.72 (m, 8 H, $BArF_{24}$; *ortho*), 7.56 (m, 4 H, $BArF_{24}$; *para*), 7.40 (t, $^3J_{HH}$ = 7.7 Hz, 4 H, *p*-Dipp), 7.18 (d, $^3J_{HH}$ = 7.7 Hz, $^4J_{HH}$ = 2.2 Hz, 8 H, *m*-Dipp), 6.44 (s, 4 H, N-C*H*=C*H*-N), 4.18 (d, $^2J_{HP}$ = 2.8 Hz, 1H, 5), 3.47 (m, 2 H, 9), 2.60 (m, 4 H, *i*Pr-C*H*), 2.53 (m, 4 H, *i*Pr-C*H*), 1.51 (s, 3H, 7), 1.25 (t, $^3J_{HH}$ = 7.0 Hz, 2 H, 10), 1.08 (d, $^3J_{HH}$ = 6.9 Hz, 12 H, *i*Pr-CH_3), 1.05 (d, $^3J_{HH}$ = 6.9 Hz, 12 H, *i*Pr-CH_3), 1.02 (d, $^3J_{HH}$ = 6.9 Hz, 12 H, *i*Pr-CH_3), 0.99 (d, $^3J_{HH}$ = 6.9 Hz, 12 H, *i*Pr-CH_3).

^{13}C{^{1}H} NMR (CD_2Cl_2, 126 MHz, 300 K): δ (ppm) = 164.6 (d, $^2J_{CP}$ = 6 Hz, 2), 162.2 (q, $^1J_{CB}$ = 50 Hz, $BArF_{24}$; ipso), 161.0 (d, $^2J_{CP}$ = 17 Hz, 6), 146.6 (*o*-Dipp), 146.4 (*o*-Dipp), 142.4 (d, $^2J_{CP}$ = 29 Hz, N-C-N), 135.2 ($BArF_{24}$; *ortho*), 132.8 (*i*-Dipp), 130.7 (*p*-Dipp), 129.3 (qq, $^2J_{CF}$ = 32 Hz, $^4J_{CF}$ = 3 Hz, $BArF_{24}$; *meta*), 125.2 (*m*-Dipp), 125.0 (q, $^1J_{CF}$ = 272 Hz, $BArF_{24}$; *C*F_3), 124.9 (*m*-Dipp), 118.1 (N-*C*H=*C*H-N), 117.9 (sept, $^3J_{CF}$ = 4 Hz, $BArF_{24}$; *para*), 64.7 (9); 86.3 (d, $^1J_{CP}$ = 147 Hz, 5), 31.2 (d, $^3J_{CP}$ = 17 Hz, 7), 29.4 (*i*Pr-*C*H), 29.2 (*i*Pr-*C*H), 24.7 (*i*Pr-*C*H_3), 24.7 (*i*Pr-*C*H_3), 23.4 (*i*Pr-*C*H_3), 23.2 (*i*Pr-*C*H_3), 14.0 (10).

^{11}B NMR (CD_2Cl_2, 160 MHz, 300 K): δ (ppm) = -6.6.

^{19}F NMR (CD_2Cl_2, 471 MHz, 300 K): δ (ppm) = -62.9.

^{31}P NMR (CD_2Cl_2, 162 MHz, 300 K): δ (ppm) = -22.2 (br).

HR-MS(ESI): m/z was calculated for [$C_{60}H_{81}N_7OPS$]$^+$ as [M]$^+$: 978.59554 found was: 978.59749. Fitting isotope pattern: Yes.

Single crystal X-ray diffraction analysis: Single crystals suitable for X-ray diffraction analysis were obtained during the preparation of [**3d**][$BArF_{24}$] (*vide supra*). A molecular structure was obtained.

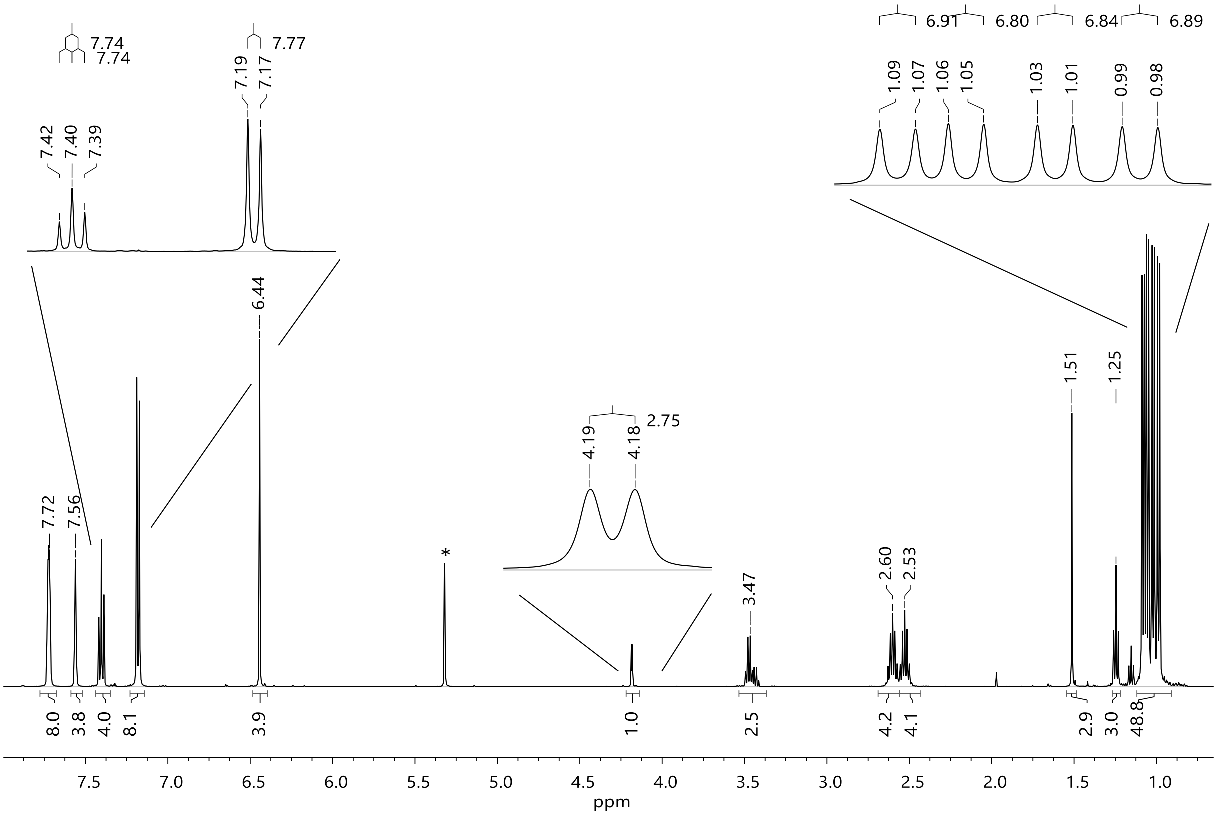

Figure S 41: ^{1}H NMR spectrum of [**3d**][BArF$_{24}$] in CD_2Cl_2. *solvent residue signal.

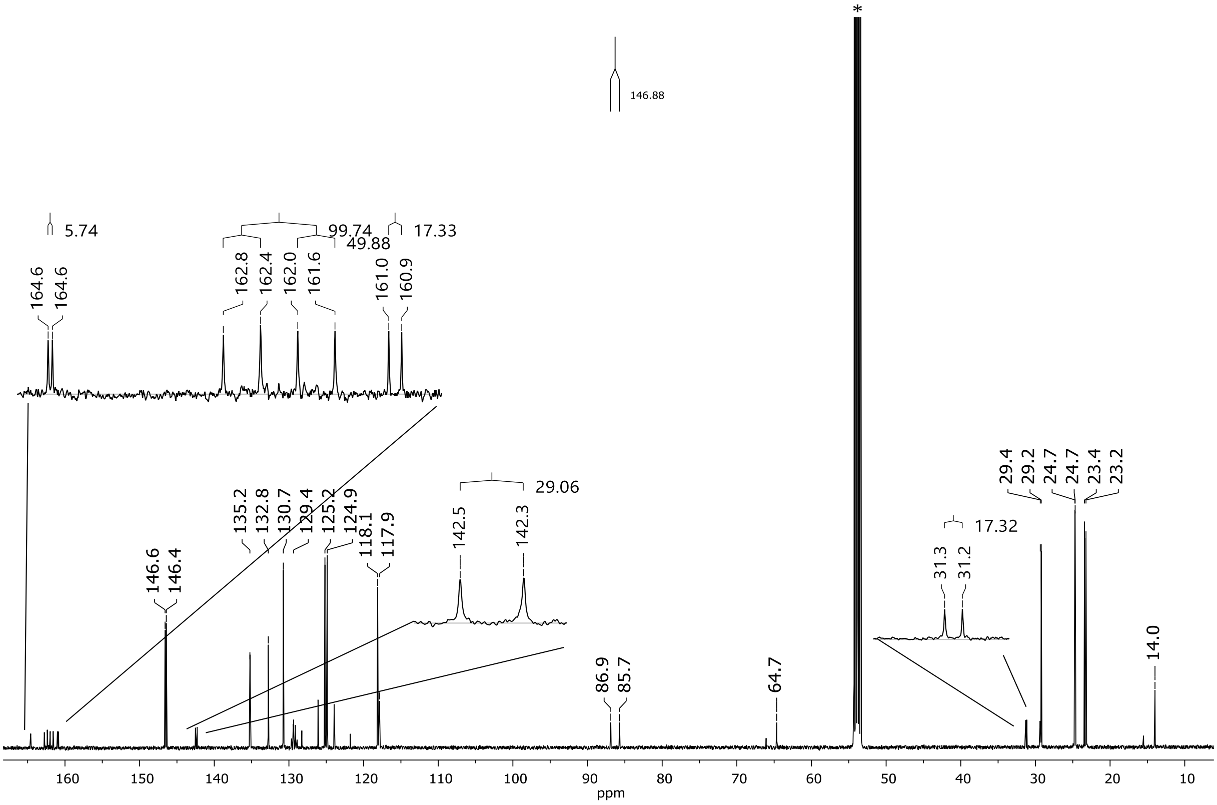

Figure S 42: ^{13}C{^{1}H} NMR spectrum of [**3d**][BArF$_{24}$] in CD_2Cl_2. *solvent residue signal.

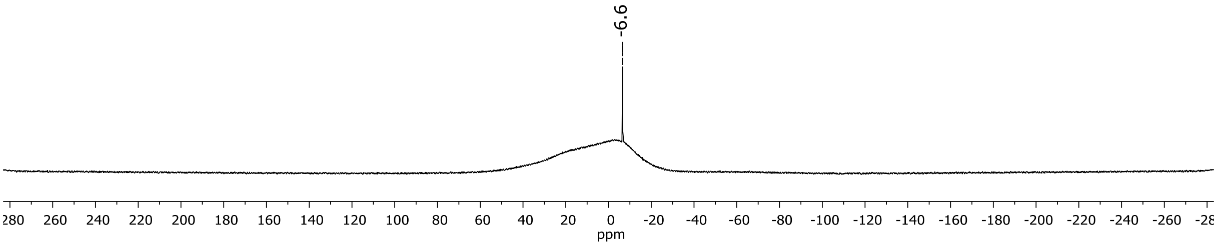

Figure S 43: ^{11}B NMR spectrum of [**3d**][$BArF_{24}$] in CD_2Cl_2.

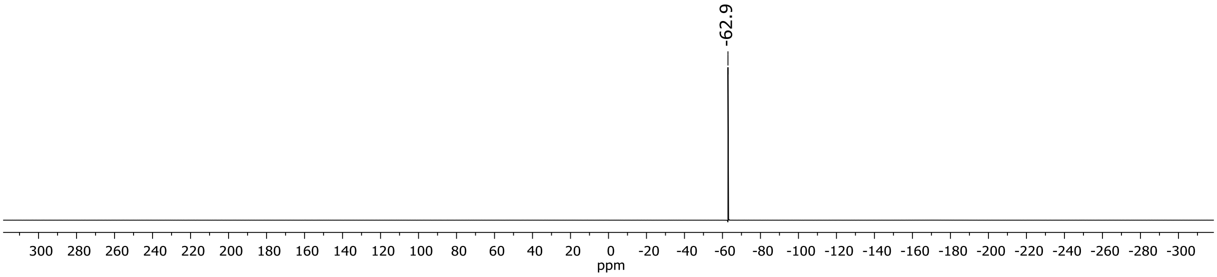

Figure S 44: ^{19}F NMR spectrum of [**3d**][$BArF_{24}$] in CD_2Cl_2.

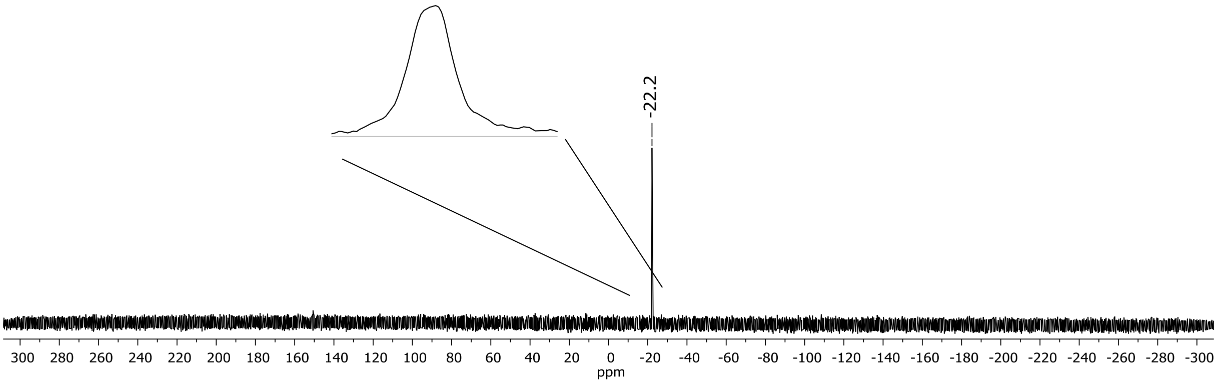

Figure S 45: ^{31}P NMR spectrum of [**3d**][$BArF_{24}$] in CD_2Cl_2.

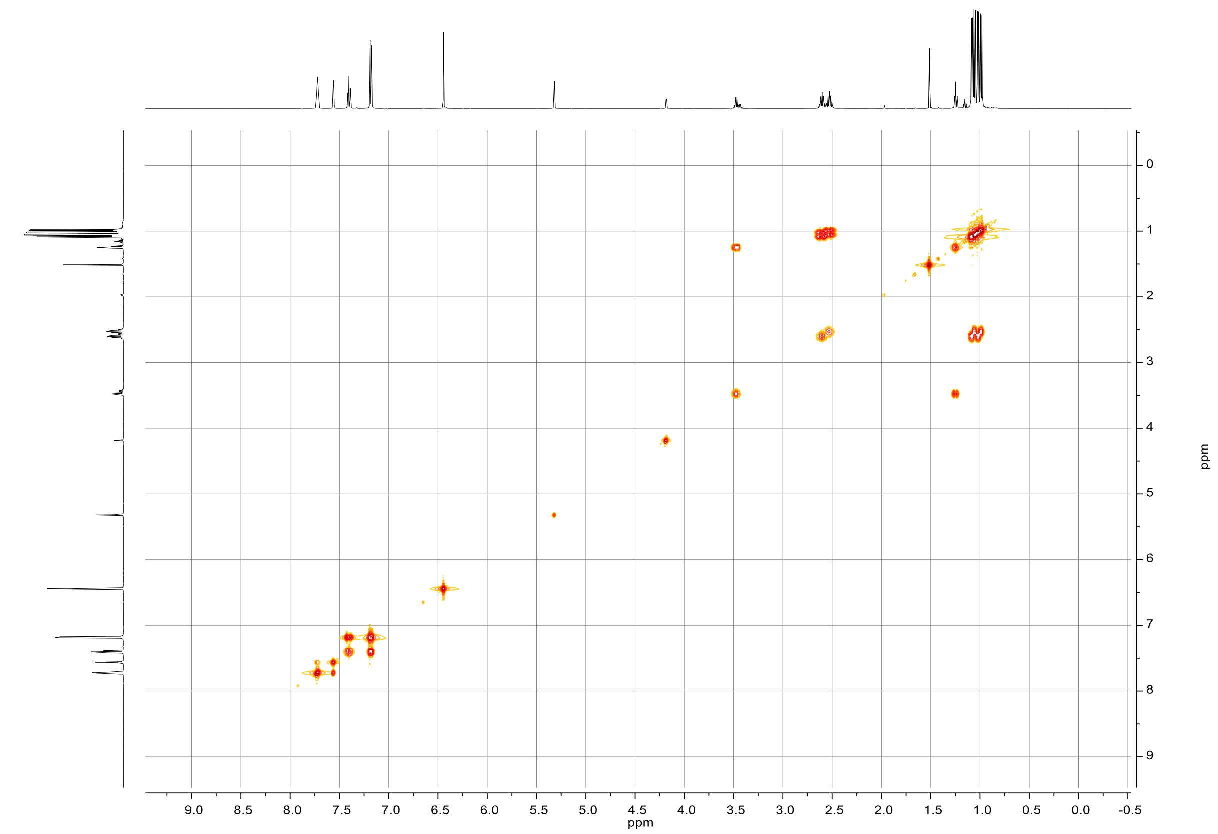

Figure S 46: $^{1}H/^{1}H$ COSY NMR spectrum of [**3d**][$BArF_{24}$] in CD_2Cl_2.

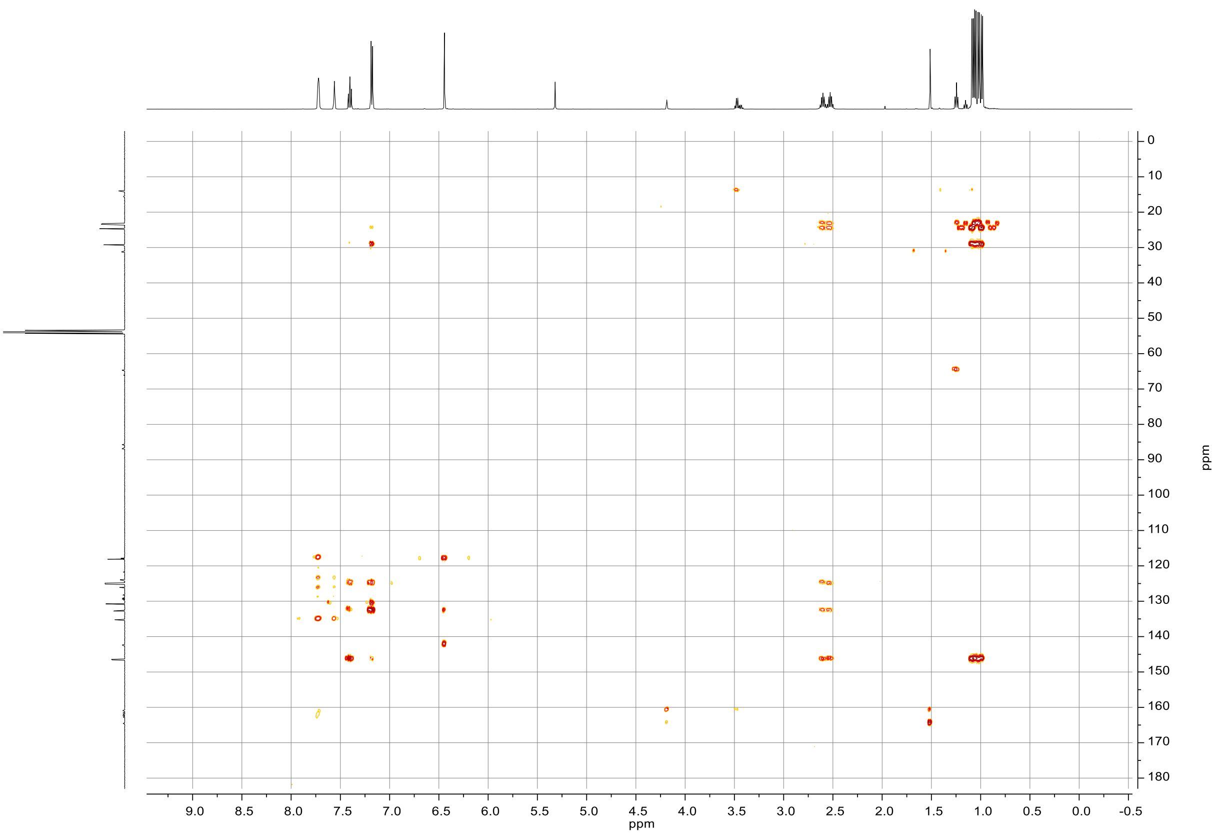

Figure S 47: $^{1}H/^{13}C$ HMBC NMR spectrum of [**3d**][$BArF_{24}$] in CD_2Cl_2.

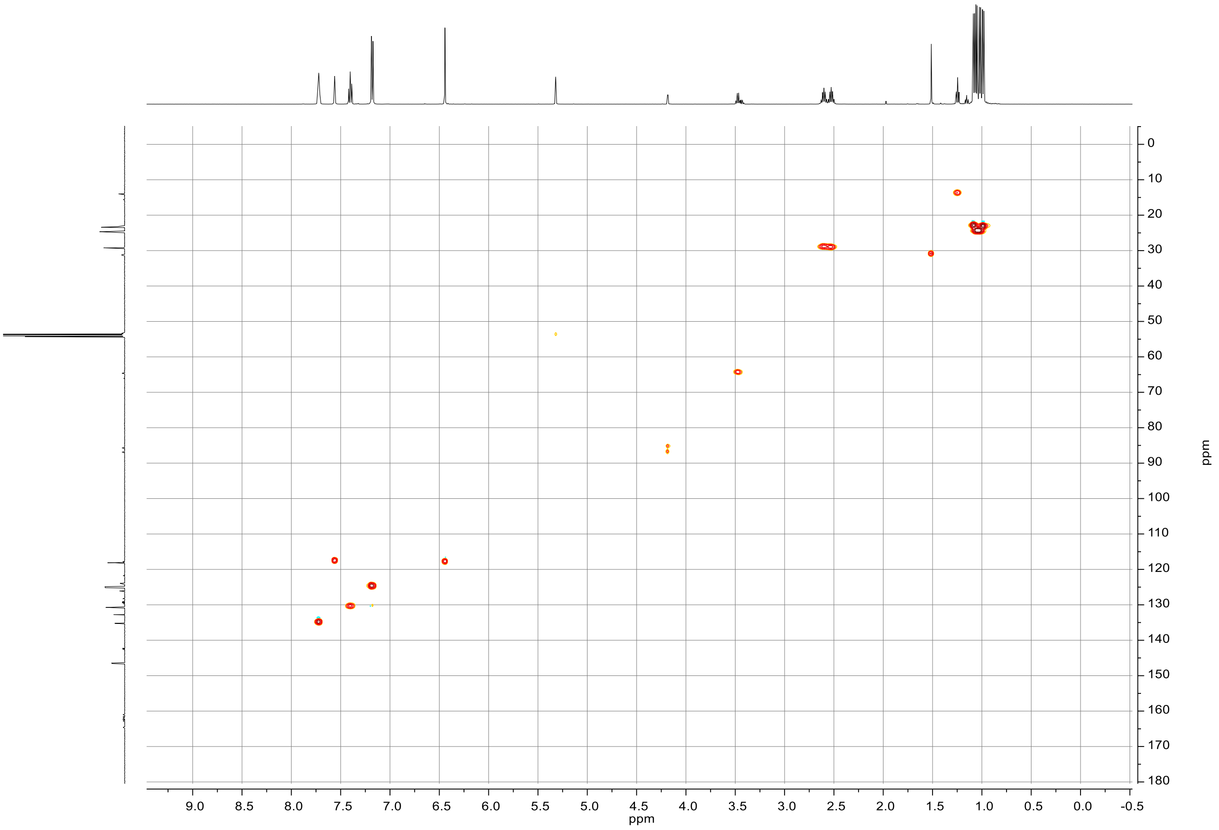

Figure S 48: ^{1}H/^{13}C HSQC NMR spectrum of [**3d**][BArF$_{24}$] in CD_2Cl_2.

2 X-ray Diffraction Studies

General:

Single-crystal X-ray diffraction data were collected on a Bruker AXS detector using Mo-K$_\alpha$ radiation (λ = 0.71073 Å). Crystals were selected under oil, mounted on glass capillaries and then immediately placed in a cold stream of N_2 on a diffractometer. The APEX2 software was used to operate the diffractometer.[2] Using Olex2,[3] the structures were solved with ShelXS or ShelXT structure solution program using and refined with the ShelXL refinement package using Least Squares minimisation.[4]

Crystallographic data have been deposited with the Cambridge Crystallographic Data Centre as supplementary publication no. 2094374 ([**2a**][BArF$_{24}$]), 2094372 ([**2d**][BArF$_{24}$]), 2094373 ([**3a**][BArF$_{24}$]), 2094371 ([**3d**][BArF$_{24}$]). These data can be obtained free of charge via www.ccdc.cam.uk/data_request/cif (or from the CCDC, 12 Union Road, Cambridge CB2 1EZ, UK; fax: (+44) 1223-336-033; or deposit@ccdc.cam.ac.uk).

2.1 **Crystal structure data of compound [2a][BArF24]**

**CCDC deposition number**	2094374
**Empirical formula**	$C_{95}H_{92}BCl_2F_{24}N_6PS$
**Formula weight**	1918.48
**Temperature/K**	100
**Crystal system**	triclinic
**Space group**	P-1
**a/Å**	12.5206(2)
**b/Å**	17.3977(3)
**c/Å**	21.7965(4)
**α/°**	90.7200(10)
**β/°**	103.6090(10)
**γ/°**	92.4100(10)
**Volume/Å^3**	4609.31(14)
**Z**	2

**ρ_{calc}g/cm^3**	1.382
**μ/mm^{-1}**	0.209
**F(000)**	1980.0
**Crystal size/mm^3**	0.318 × 0.241 × 0.194
**Radiation**	MoKα (λ = 0.71073)
**2Θ range for data collection/°**	1.922 to 56.626
**Index ranges**	$-16 \le h \le 16$, $-23 \le k \le 23$, $-29 \le l \le 29$
**Reflections collected**	73272
**Independent reflections**	22859 [R_{int} = 0.0268, R_{sigma} = 0.0283]
**Data/restraints/parameters**	22859/0/1187
**Goodness-of-fit on F^2**	1.030
**Final R indexes [I>=2σ (I)]**	R_1 = 0.0572, wR_2 = 0.1469
**Final R indexes [all data]**	R_1 = 0.0680, wR_2 = 0.1546
**Largest diff. peak/hole / e Å^{-3}**	1.57/-1.06

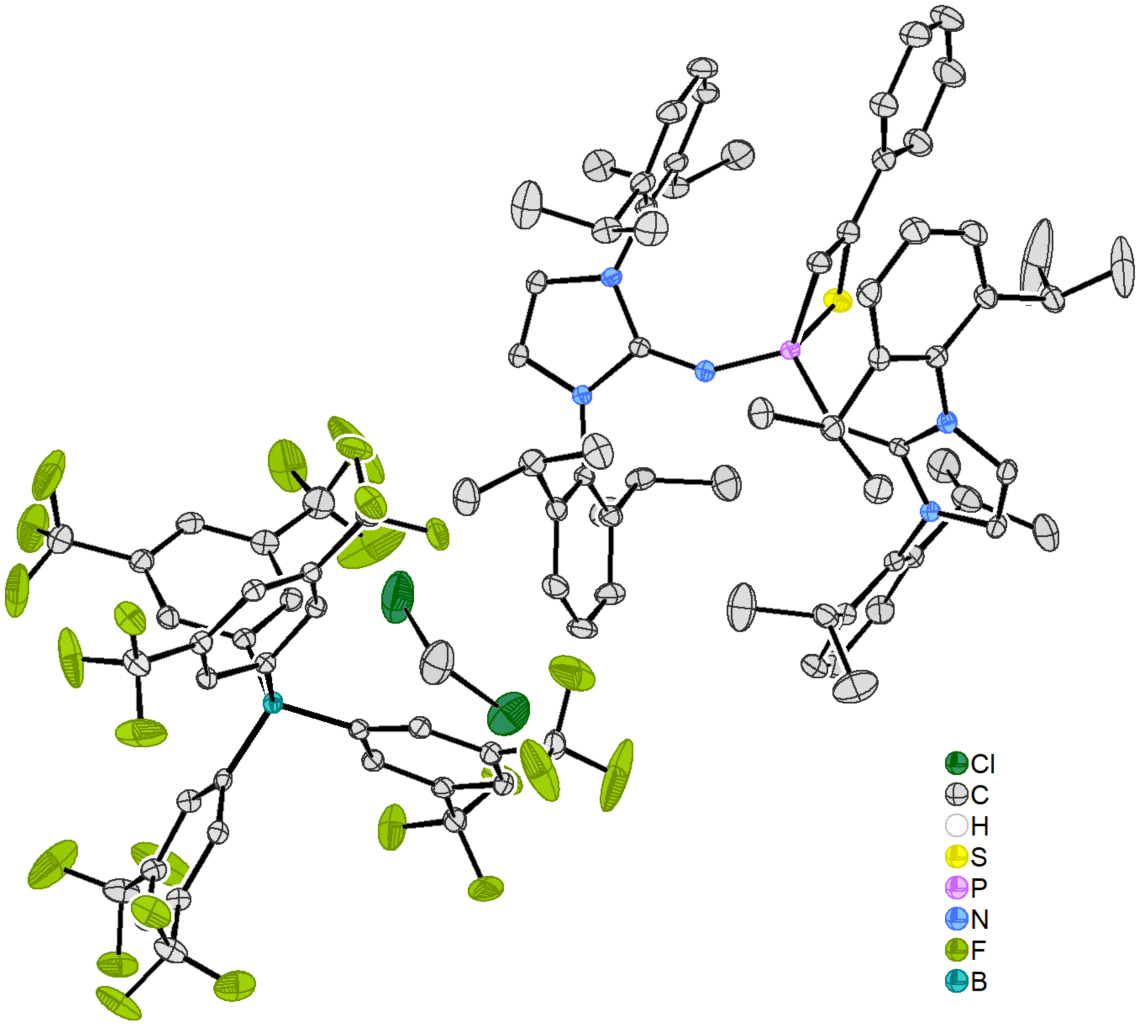

The asymmetric unit contains one molecule of [**2a**][BArF24] and a dichloromethane solvate molecule.

2.2 Crystal structure data of compound [2d][BArF24]

**CCDC deposition number**	209437	**ρ_{calc}g/cm^3**	1.340
**Empirical formula**	$C_{90}H_{89}BF_{24}N_6OPS$	**μ/mm^{-1}**	0.154
**Formula weight**	1800.51	**F(000)**	3724.0
**Temperature/K**	100	**Crystal size/mm^3**	0.509 × 0.401 × 0.211
**Crystal system**	monoclinic	**Radiation**	MoKα (λ = 0.71073)
**Space group**	$P2_1/n$	**2Θ range for data collection/°**	2.73 to 55.884
**a/Å**	18.4629(4)	**Index ranges**	-24 ≤ h ≤ 24, -34 ≤ k ≤ 34, -25 ≤ l ≤ 25
**b/Å**	26.1753(6)	**Reflections collected**	136235
**c/Å**	19.1319(4)	**Independent reflections**	21397 [R_{int} = 0.0489, R_{sigma} = 0.0291]
**α/°**	90	**Data/restraints/parameters**	21397/0/1183
**β/°**	105.1740(10)	**Goodness-of-fit on F^2**	1.017
**γ/°**	90	**Final R indexes [I>=2σ (I)]**	R_1 = 0.0488, wR_2 = 0.1214
**Volume/Å^3**	8923.6(3)	**Final R indexes [all data]**	R_1 = 0.0590, wR_2 = 0.1291
**Z**	4	**Largest diff. peak/hole / e Å^{-3}**	0.91/-0.67

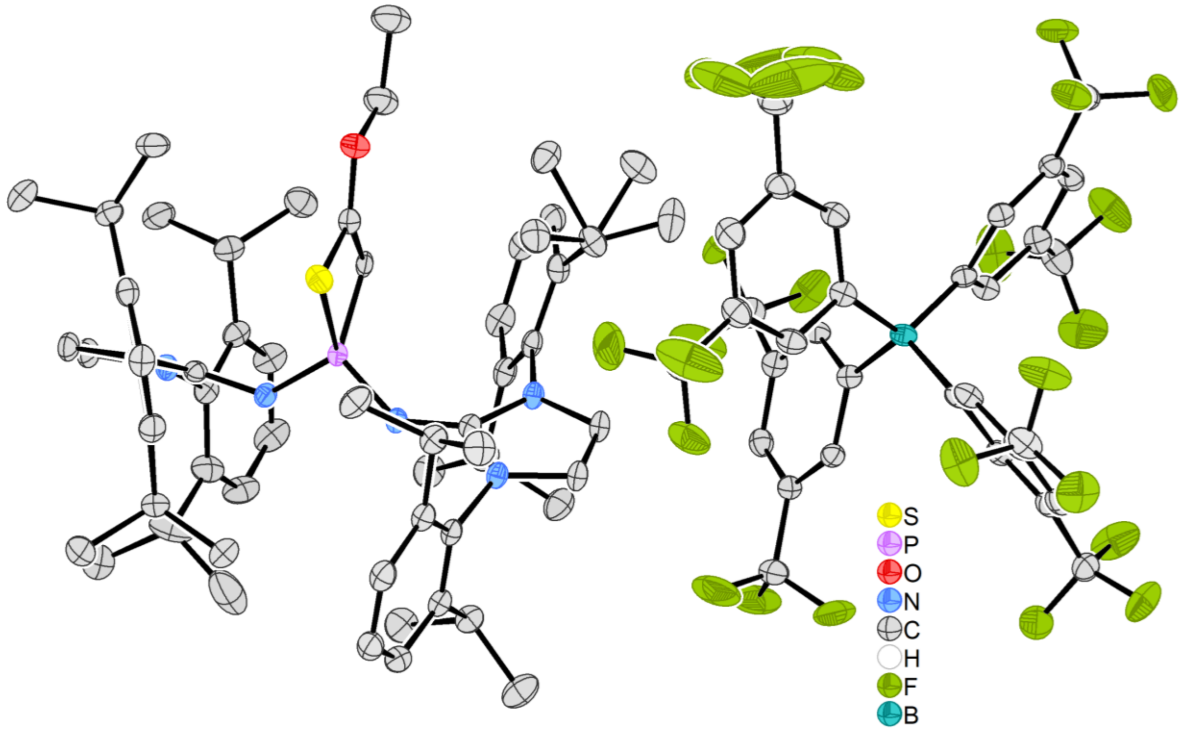

The asymmetric unit contains one molecule of [**2d**][BArF24] and 0.5 molecules of dichloromethane. The dichloromethane molecule is severely disordered and was therefore treated with the solvent mask program implemented in OLEX2. 90 electrons were found in a volume of 566 A^3 in 3 voids per unit cell, which is consistent with the presence of 0.5 dichloromethane per asymmetric unit.

2.3 Crystal structure data of compound [3a][$BArF_{24}$]

**CCDC deposition number**	2094373
**Empirical formula**	$C_{96}H_{93}BF_{24}N_7PS$
**Formula weight**	1874.61
**Temperature/K**	100
**Crystal system**	monoclinic
**Space group**	$P2_1/c$
**a/Å**	14.3601(5)
**b/Å**	16.5099(6)
**c/Å**	38.0457(13)
**α/°**	90
**β/°**	90.795(2)
**γ/°**	90
**Volume/Å^3**	9019.2(5)
**Z**	4

**ρ_{calc}g/cm^3**	1.381
**μ/mm^{-1}**	0.155
**F(000)**	3880.0
**Crystal size/mm^3**	0.605 × 0.378 × 0.3
**Radiation**	MoKα (λ = 0.71073)
**2Θ range for data collection/°**	2.142 to 56.05
**Index ranges**	$-18 \leq h \leq 18$, $-21 \leq k \leq 21$, $-50 \leq l \leq 50$
**Reflections collected**	133693
**Independent reflections**	21694 [R_{int} = 0.0599, R_{sigma} = 0.0382]
**Data/restraints/parameters**	21694/0/1188
**Goodness-of-fit on F^2**	1.063
**Final R indexes [I>=2σ (I)]**	R_1 = 0.0628, wR_2 = 0.1653
**Final R indexes [all data]**	R_1 = 0.0782, wR_2 = 0.1771
**Largest diff. peak/hole / e Å^{-3}**	0.72/-0.34

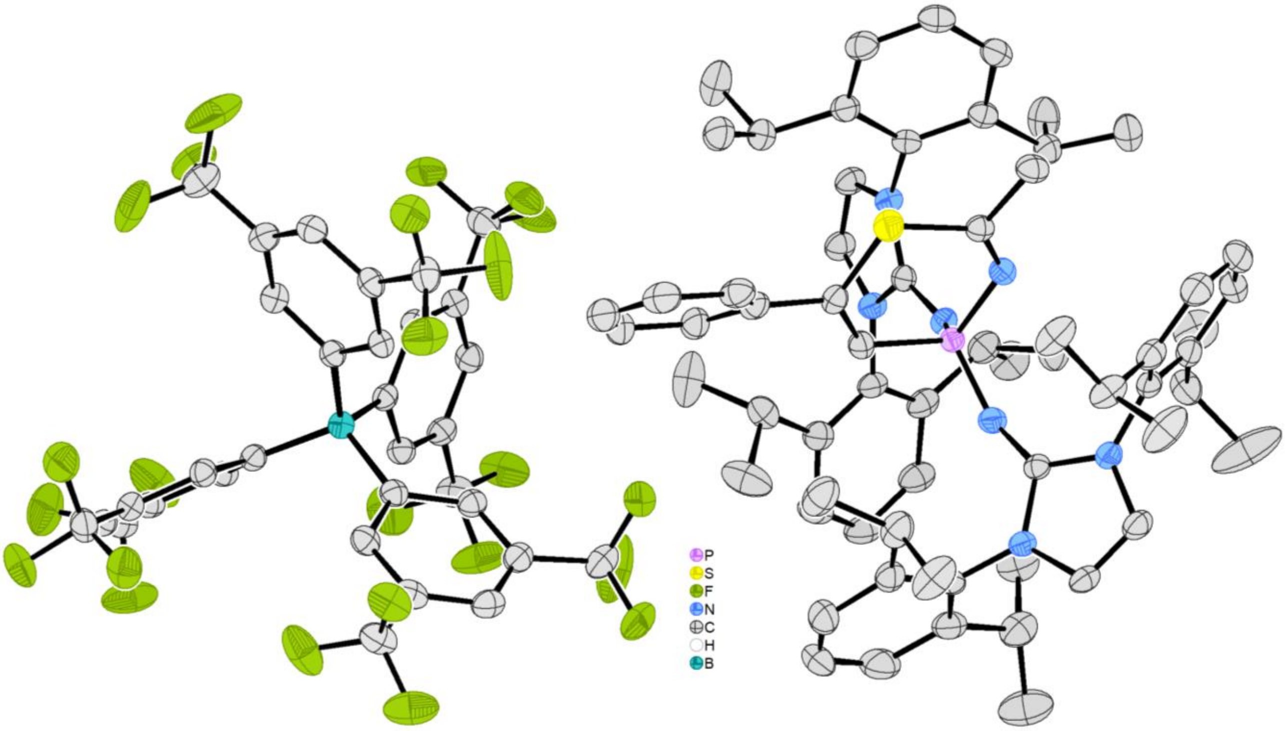

The asymmetric unit contains one molecule of [**3a**][$BArF_{24}$].

2.4 Crystal structure data of compound [3d][BArF$_{24}$]

**CCDC deposition number**	2094371
**Empirical formula**	$C_{92}H_{93}BF_{24}N_7OPS$
**Formula weight**	1842.57
**Temperature/K**	140.0
**Crystal system**	monoclinic
**Space group**	$P2_1/c$
**a/Å**	21.5733(9)
**b/Å**	20.6074(8)
**c/Å**	21.1572(8)
**α/°**	90
**β/°**	105.0070(10)
**γ/°**	90
**Volume/Å^3**	9085.1(6)
**Z**	4

**ρ_{calc}g/cm^3**	1.347
**μ/mm^{-1}**	1.351
**F(000)**	3816.0
**Crystal size/mm^3**	0.87 × 0.439 × 0.08
**Radiation**	CuKα (λ = 1.54178)
**2Θ range for data collection/°**	6.032 to 149.656
**Index ranges**	-24 ≤ h ≤ 25, -25 ≤ k ≤ 25, -26 ≤ l ≤ 26
**Reflections collected**	55413
**Independent reflections**	17434 [R_{int} = 0.0416, R_{sigma} = 0.0385]
**Data/restraints/parameters**	17434/0/1162
**Goodness-of-fit on F^2**	1.017
**Final R indexes [I>=2σ (I)]**	R_1 = 0.0696, wR_2 = 0.1856
**Final R indexes [all data]**	R_1 = 0.0753, wR_2 = 0.1914
**Largest diff. peak/hole / e Å^{-3}**	1.28/-0.73

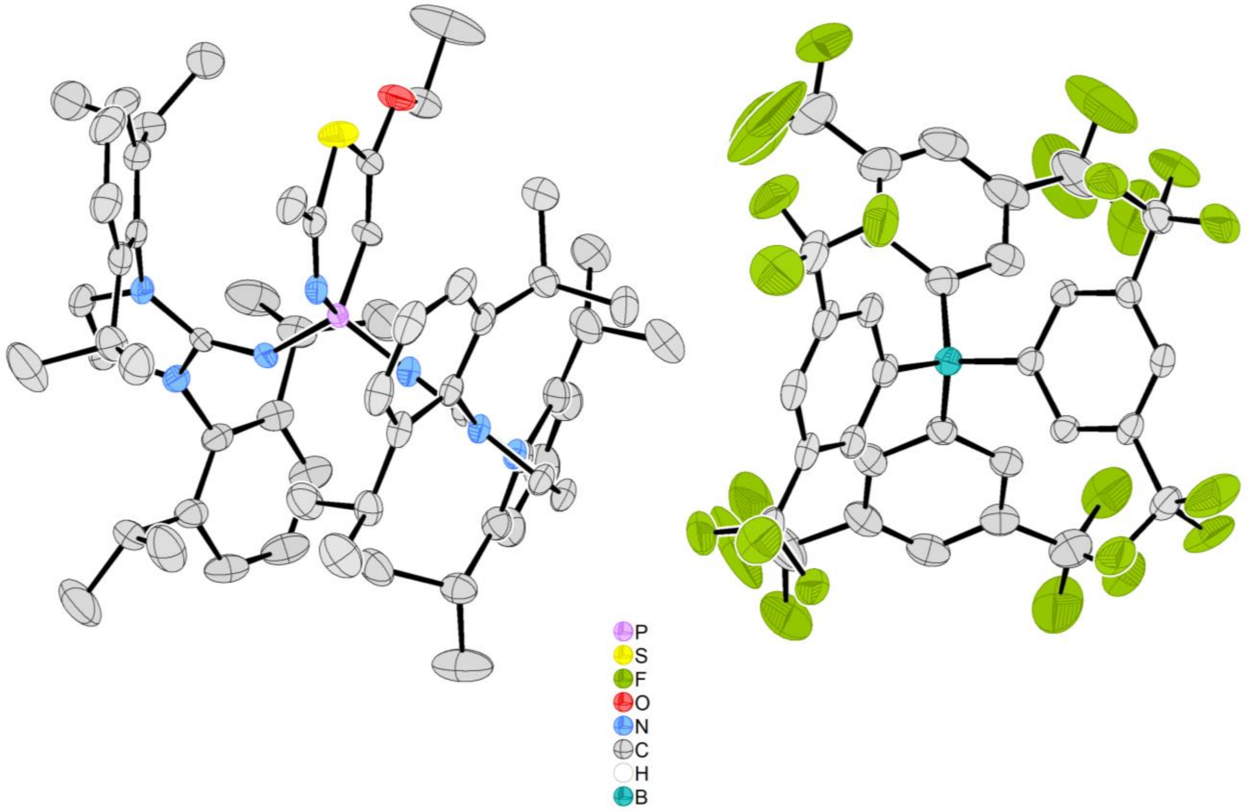

The asymmetric unit contains one molecule of [**3b**][BArF$_{24}$].

3 Computational studies

3.1 General

Geometry optimizations were performed using the B3LYP[5,6] functional in combination with the D3BJ dispersion correction[7] using the def2-SVP[8,9] basis set. Frequency calculations at the same level of theory were performed to confirm the minima (zero imaginary frequencies) and the transition states (one imaginary frequency). Intrinsic reaction coordinate (IRC) calculations were run to confirm that the transition states connect appropriate minima. The total free energies have been calculated as follows:

$$\Delta G = \Delta E_{el} + \Delta E_{ZPE} + \Delta E_{therm} + \Delta E_{entropy} + \Delta E_{solv} \text{ (SMD, fluorobenzene)}$$

The electronic energy ΔE_{el} was obtained using DLPNO-CCSD(T)[10–15] with the def2-TZVPP[8,9] basis set. These results were corrected to include zero-point correction (ΔE_{ZPE}), thermal correction (ΔE_{therm}) and entropy correction ($\Delta E_{entropy}$) all obtained from the geometry optimizations with B3LYP-D3/def2-SVP. In the case of the entropy correction, the results were further corrected to account for small frequencies as suggested by Truhlar[16] with a threshold of 100 cm^{-1} using the GoodVibes program[17] developed by Robert Paton and Ignacio Funes-Ardois. At the end, solvent effects were obtained from B3LYP-D3 calculations with the def2-TZVPP basis set using the SMD model[18] with fluorobenzene as solvent.

Natural Population Analyses (NPA)[19] at the B3LYP-D3/def2-TZVPP level of theory have been performed to obtain natural charges. These calculations have been performed using NBO 3.1 program[20] linked through Gaussian 16.

All DFT calculations have been performed using the Gaussian 16 software.[21] The DLPNO-CCSD(T) calculations have been carried out using the ORCA 4.2 software.[22]

The chalcogenophosphonium model compounds employed in the calculations are shown in Scheme 1.

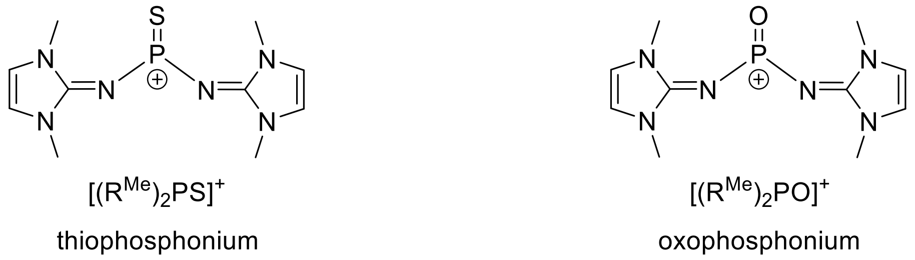

Scheme S 1: Model compounds used in the computational studies in this chapter.

3.2 Calculated energies

Table S1: B3LYP-D3BJ/def2-SVP electronic energies in kcal/mol for reactions of thiophosphonium with different acetylene derivatives. Separated reactants have been used as a reference.

	**HCCPh**	**HCCOEt**	**HCC(CF_3)**
**SR**	0.0	0.0	0.0
**RC0**		-13.5	
**TS0**		-12.7	
**RC**	-15.1	-12.8	-9.6
**TS1**	-1.1	-11.1	16.4
**CF**	-37.7	-46.9	-35.0
**TS2**	-20.9	-36.6	-21.9
**OF**	-21.6	-40.6	-23.4

Table S2: DLPNO-CCSD(T)/def2-TZVPP results including corrections to Gibbs free energy for reactions of thiophosphonium with different acetylene derivatives in kcal/mol. Separated reactants have been used as a reference.

	**HCCNMe$_2$**	**HCCPh**	**HCCOEt**	**HCC(CF_3)**
**SR**	0.0	0.0	0.0	0.0
**RC0**			-15.6	
**TS0**			-14.5	
**RC**	-32.3	-16.7	-13.2	-14.0
**TS1**	-29.3	-0.8	-14.2	14.4
**CF**	-49.2	-39.6	-50.4	-39.7
**TS2**	-35.6	-21.7	-37.5	-24.1
**OF**	-35.9	-21.2	-39.7	-25.0

Table S3: DLPNO-CCSD(T)/def2-TZVPP electronic energies ΔE_{el}, electronic energies including solvent model (ΔE_{solv}), free enthalpy (ΔH) and free energy (ΔG) in kcal/mol for reaction of thiophosphonium model complex with (dimethylamino)acetylene. Corrections have been calculated at the B3LYP-D3 level of theory.

	ΔE_{el}	ΔE_{solv} (SMD, fluorobenzene)	ΔH	ΔG
**SR**	0.0	0.0	0.0	0.0
**RC**	-21.3	-19.8	-18.3	-32.3
**TS1**	-18.1	-15.4	-14.5	-29.3
**CF**	-43.5	-36.4	-34.3	-49.2
**TS2**	-26.8	-21.4	-20.0	-35.6
**OF**	-28.3	-23.3	-21.1	-35.9

Table S4: DLPNO-CCSD(T)/def2-TZVPP electronic energies ΔE_{el}, electronic energies including solvent model (ΔE_{solv}), free enthalpy (ΔH) and free energy (ΔG) in kcal/mol for reaction of thiophosphonium model complex with phenylacetylene. Corrections have been calculated at the B3LYP-D3 level of theory.

	ΔE_{el}	ΔE_{solv} (SMD, fluorobenzene)	ΔH	ΔG
**SR**	0.0	0.0	0.0	0.0
**RC**	-10.6	-4.2	-2.8	-16.7
**TS1**	10.8	14.5	14.5	-0.8
**CF**	-32.1	-25.7	-23.9	-39.6
**TS2**	-10.6	-6.3	-5.3	-21.7
**OF**	-11.4	-7.6	-5.9	-21.2

Table S5: DLPNO-CCSD(T)/def2-TZVPP electronic energies ΔE_{el}, electronic energies including solvent model (ΔE_{solv}), free enthalpy (ΔH) and free energy (ΔG) in kcal/mol for reaction of thiophosphonium model complex with ethoxyacetylene. Corrections have been calculated at the B3LYP-D3 level of theory.

	ΔE_{el}	ΔE_{solv} (SMD, fluorobenzene)	ΔH	ΔG
**SR**	-8.5	-4.2	-3.0	-15.5
**RC0**	-5.7	-0.9	-0.6	-14.5
**TS0**	-5.1	-0.6	0.3	-13.2
**RC**	-3.4	0.5	0.6	-14.2
**TS1**	-42.9	-37.3	-35.5	-50.4
**CF**	-27.2	-22.6	-21.6	-37.5
**TS2**	-31.5	-26.9	-25.1	-39.7
**OF**	-8.5	-4.2	-3.0	-15.5

Table S6: DLPNO-CCSD(T)/def2-TZVPP electronic energies ΔE_{el}, electronic energies including solvent model (ΔE_{solv}), free enthalpy (ΔH) and free energy (ΔG) in kcal/mol for reaction of thiophosphonium model complex with (trifluoromethyl)acetylene. Corrections have been calculated at the B3LYP-D3 level of theory.

	ΔE_{el}	ΔE_{solv} (SMD, fluorobenzene)	ΔH	ΔG
**SR**	0.0	0.0	0.0	0.0
**RC**	-4.4	-2.0	-0.7	-14.0
**TS1**	27.1	29.2	29.1	14.4
**CF**	-29.0	-26.2	-24.4	-39.7
**TS2**	-8.7	-8.8	-7.7	-24.1
**OF**	-11.0	-11.5	-9.7	-25.0

Table S7: DLPNO-CCSD(T)/def2-TZVPP results including corrections to Gibbs free energy in kcal/mol for oxo- and thiophosphonium reactions with phenylacetylene. Separated reactants have been used as a reference. The energies of the oxophophonium reaction have been taken from literature.[23]

	oxophosphonium	thiophosphonium
**SR**	0.0	0.0
**RC**	-19.2	-16.7
**TS1**	-2.4	-0.8
**CF**	-36.6	-39.6
**TS2**	-17.5	-21.7
**OF**	-23.3	-21.2

Table S8: DLPNO-CCSD(T)/def2-TZVPP electronic energies ΔE_{el}, electronic energies including solvent model (ΔE_{solv}), free enthalpy (ΔH) and free energy (ΔG) in kcal/mol for reaction of the oxophosphonium model complex with (dimethylamino)acetylene. Corrections have been calculated at the B3LYP-D3 level of theory.

	ΔE_{el}	ΔE_{solv} (SMD, fluorobenzene)	ΔH	ΔG
**SR**	0.0	0.0	0.0	0.0
**RC**	-27.3	-23.3	-21.7	-36.1
**TS1**	-19.7	-14.1	-13.2	-28.3
**CF**	-42.5	-35.2	-32.8	-47.6
**TS2**	-25.8	-18.4	-16.8	-32.2
**OF**	-33.6	-27.0	-24.6	-38.8

Table S9: DLPNO-CCSD(T)/def2-TZVPP electronic energies ΔE_{el}, electronic energies including solvent model (ΔE_{solv}), free enthalpy (ΔH) and free energy (ΔG) in kcal/mol for reaction of the oxophosphonium model complex with ethoxyacetylene. Corrections have been calculated at the B3LYP-D3 level of theory.

	**ΔE_{el}**	**ΔE_{solv} (SMD, fluorobenzene)**	**ΔH**	**ΔG**
**SR**	0.0	0.0	0.0	0.0
**RC**	-10.2	-5.8	-5.0	-19.3
**TS1**	-7.8	-2.7	-2.5	-17.7
**CF**	-41.7	-35.7	-33.4	-48.8
**TS2**	-25.3	-21.7	-20.3	-36.1
**OF**	-41.4	-36.3	-34.0	-48.2

Table S10: DLPNO-CCSD(T)/def2-TZVPP results including corrections to Gibbs free energy in kcal/mol for oxo- and thiophosphonium reactions with phenylacetylene. Separated reactants have been used as a reference.

	**ethoxyacetylene**		**(dimethylamino)acetylene**	
	**oxophosphonium**	**thiophosphonium**	**oxophosphonium**	**thiophosphonium**
**SR**	0.0	0.0	0.0	0.0
**RC0**		-15.6		
**TS0**		-14.5		
**RC**	-19.3	-13.2	-36.1	-32.3
**TS1**	-17.7	-14.2	-28.3	-29.3
**CF**	-48.8	-50.4	-47.6	-49.2
**TS2**	-36.1	-37.5	-32.3	-35.6
**OF**	-48.2	-39.7	-38.8	-35.9

3.3 **Natural population Analysis**

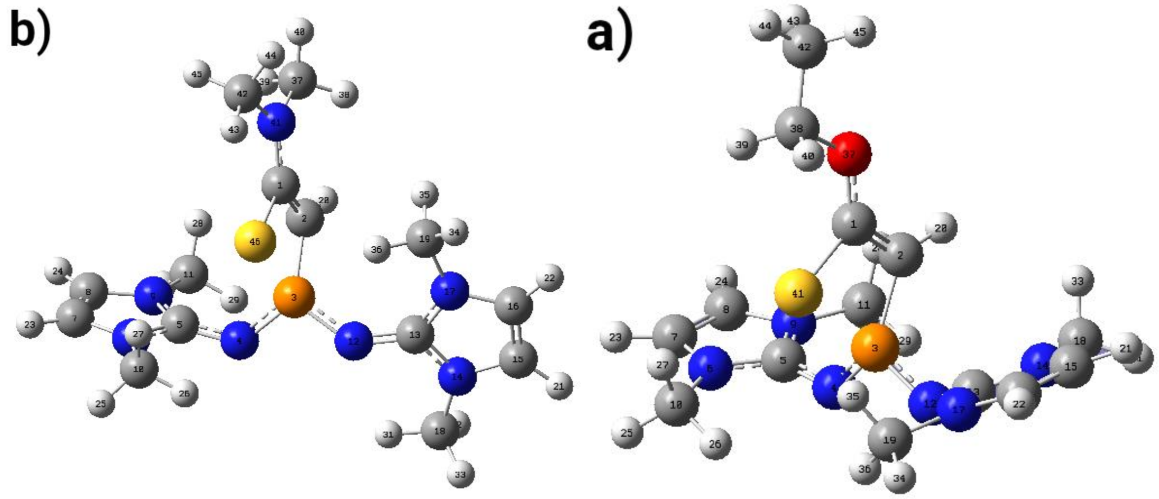

Figure S 49: Closed form complex of thiophosphonium ion with a) etoxyacetylene and b) (dimethylamino)acetylene. The atom labels used in NPA analysis are also shown.

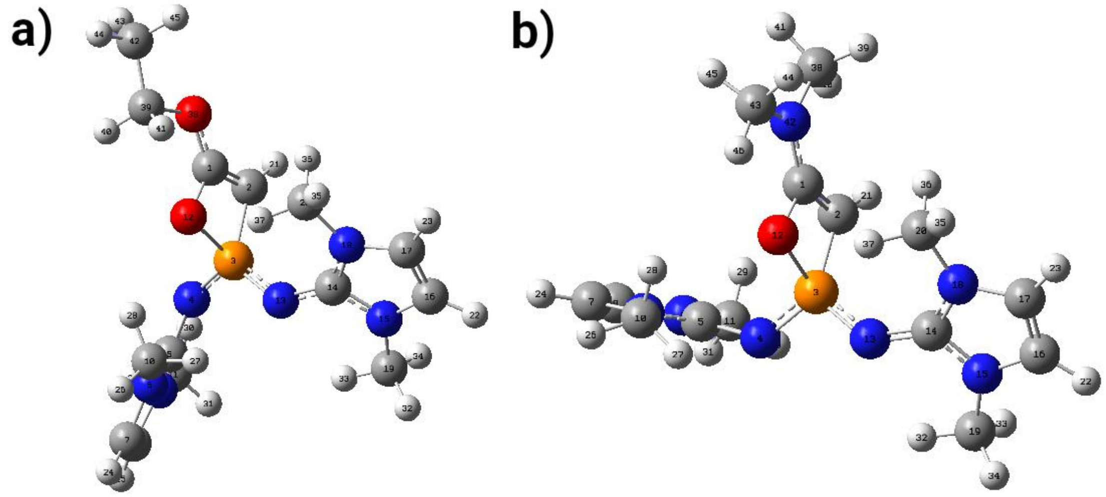

FigureS 50: Closed form complex of oxophosphonium ion with a) etoxyacetylene and b) (dimethylamino)acetylene. The atom labels used in NPA analysis are also shown.

Table S11: NPA charges in *e* (elementary charges) obtained from B3LYP-D3BJ/def2-TZVPP calculations. The atom lables are the same as in Figure S48 and Figure S49, respectively.

	**RC**	**TS1**	**CF**	**TS2**	**OF**
**thiophosphonium**					
etoxyacetylene					
P3	1.721	1.775	1.897	1.962	1.960
S41	-0.673	-0.708	-0.184	-0.280	-0.226
C2	-0.584	-0.667	-0.806	-0.924	-0.902
C1	0.550	0.641	0.258	0.220	0.205
N4	-1.001	-1.014	-1.003	-0.959	-0.974
N12	-1.000	-1.005	-1.003	-0.956	-0.971
(dimethylamino)acetylene					
P3	1.734	1.788	1.894	1.968	1.965
S46	-0.714	-0.663	-0.143	-0.308	-0.280
C2	-0.545	-0.595	-0.851	-0.943	-0.917
C1	0.441	0.452	0.142	0.107	0.092
N4	-1.011	-1.006	-1.004	-0.968	-0.974
N12	-1.019	-1.021	-1.003	-0.960	-0.971
**oxophosphonium**					
etoxyacetylene					
P3	2.249	2.253	2.145	2.036	1.955
O12	-1.126	-1.121	-0.750	-0.724	-0.655
C2	-0.631	-0.673	-0.827	-0.984	-0.963
C1	0.621	0.680	0.646	0.727	0.735
N4	-1.012	-1.015	-1.008	-0.961	-0.979
N13	-1.038	-1.042	-1.046	-0.994	-0.975
(dimethylamino)acetylene					
P3	2.236	2.236	2.153	2.036	1.959
O12	-1.146	-1.071	-0.752	-0.748	-0.694
C2	-0.552	-0.596	-0.892	-1.016	-0.967
C1	0.447	0.505	0.534	0.602	0.619
N4	-1.022	-1.019	-1.013	-0.965	-0.978
N13	-1.044	-1.046	-1.010	-0.963	-0.975

3.4 **Computed Energies (au) and Cartesian Coordinates (Å)**

Species are numbered according to the numbering scheme in the main text. Electronic energies and thermal corrections to enthalpy refer to B3LYP-D3/def2-SVP data. Entropy corrections refer to B3LYP-D3/def2-SVP data corrected for the small frequencies as explained above. All energies are shown in kcal/mol.

Thiophosphonium model compound $[(R^{Me})_2PS]^+$

Separated reactant (SRa)

Electronic energy = -1457.93859593

Thermal correction to enthalpy = 0.293751

Entropy correction = 0.066312

Lowest frequency = 23.43 cm^{-1}

Number of atoms: 34

charge=+1, multiplicity=1

P	0.000019	-0.000601	0.146042
N	1.295293	-0.013416	-0.769603
C	2.578030	0.040109	-0.408017
N	3.229073	1.003455	0.301331
C	4.581462	0.693033	0.356240
C	4.760583	-0.466070	-0.331951
N	3.518288	-0.858273	-0.806480
C	2.607497	2.198272	0.854484
C	3.232485	-2.049741	-1.591496
S	-0.000546	-0.002297	2.080182
N	-1.295091	0.014295	-0.769713
C	-2.577849	-0.039439	-0.408292
N	-3.229228	-1.003522	0.299710
C	-4.581507	-0.692707	0.355074
C	-4.760232	0.467409	-0.331515
N	-3.517803	0.859832	-0.805512
C	-2.608113	-2.199266	0.851390
C	-3.231697	2.052211	-1.589032
H	-5.295300	-1.327162	0.871892
H	-5.659723	1.044056	-0.525748
H	5.295058	1.327009	0.873917
H	5.660282	-1.042117	-0.526997
H	3.381507	2.961145	1.002616
H	1.864019	2.581427	0.141964
H	2.110696	1.975125	1.808754
H	4.003512	-2.173312	-2.363600
H	3.211730	-2.942813	-0.948846
H	2.253333	-1.919045	-2.067940
H	-3.382363	-2.962130	0.998331
H	-1.864599	-2.581672	0.138505
H	-2.111438	-1.977475	1.806038
H	-4.002086	2.176374	-2.361685
H	-3.211761	2.944608	-0.945424
H	-2.252101	1.922286	-2.064766

Reaction of $[(R^{Me})_2PS]^+$ with phenylacetylene

Phenylacetylene

Electronic energy = -308.1999708

Thermal correction to enthalpy = 0.117135

Entropy correction = 0.037712

Lowest frequency = 142.97 cm^{-1}

Number of atoms: 14

charge=0, multiplicity=1

C	-2.215943	0.000101	0.000003
C	-1.514814	-1.209749	-0.000001
C	-0.120721	-1.214351	-0.000036
C	0.594740	-0.000113	0.000149
C	-0.120617	1.214257	0.000015
C	-1.514732	1.209851	-0.000036
C	2.026552	-0.000201	0.000046
C	3.240486	-0.000001	-0.000123
H	4.313652	0.000886	0.000162
H	0.430776	-2.155924	-0.000078
H	0.430929	2.155957	-0.000003
H	-2.058545	-2.157009	-0.000045
H	-2.058369	2.157192	-0.000173
H	-3.308147	0.000138	0.000034

Reactant complex (RCa)

Electronic energy = -1766.16266655

Thermal correction to enthalpy = 0.413148

Entropy correction = 0.081790

Lowest frequency = 12.72 cm^{-1}

Number of atoms: 48

charge=+1, multiplicity=1

C	-4.866409	1.498858	-0.826969
C	-4.814910	1.738835	0.549715

C	-3.585349	1.827185	1.201182
C	-2.384793	1.674470	0.476484
C	-2.444793	1.438293	-0.910833
C	-3.680102	1.349868	-1.552051
C	-1.129243	1.742262	1.161847
C	-0.094278	1.762673	1.798601
P	1.046222	-0.549330	-0.322456
N	2.361952	-0.484200	0.566711
C	3.546543	0.054922	0.277747
N	3.866173	1.330017	-0.085922
C	5.246489	1.440187	-0.194597
C	5.778715	0.227614	0.112585
N	4.723127	-0.619335	0.409045
C	2.934033	2.430067	-0.294067
C	4.823637	-2.022889	0.779064
S	0.931090	0.229805	-2.093619
N	-0.042035	-1.423465	0.412216
C	-1.352269	-1.592084	0.268132
N	-2.230520	-1.509907	1.306890
C	-3.513232	-1.752606	0.849060
C	-3.422962	-2.015863	-0.481621
N	-2.083260	-1.930440	-0.832202
C	-1.862273	-1.204054	2.679393
C	-1.555591	-2.206866	-2.159738
H	0.804089	1.776863	2.386110
H	-3.541583	2.030898	2.272921
H	-1.518212	1.325652	-1.474460
H	-4.376115	-1.713001	1.506073
H	-4.190430	-2.259143	-1.209426
H	5.725030	2.374037	-0.474036
H	6.812210	-0.103939	0.149442
H	-5.738639	1.868360	1.118012
H	-3.716429	1.176038	-2.630101
H	-5.831091	1.437913	-1.335579
H	3.370457	3.348694	0.120246
H	2.719242	2.560665	-1.363289
H	1.991660	2.216354	0.221583
H	5.630454	-2.152520	1.512887
H	3.867983	-2.327577	1.222376
H	5.030352	-2.643809	-0.105653
H	-2.269383	-1.973558	3.350093
H	-0.768718	-1.193572	2.741312
H	-2.249017	-0.216327	2.964895
H	-2.157186	-3.001306	-2.620371
H	-1.575451	-1.305454	-2.786772
H	-0.517320	-2.546139	-2.072299

First transition state (TS1a)

Electronic energy = -1766.14032608

Thermal correction to enthalpy = 0.410926

Entropy correction = 0.079717

Lowest frequency = -226.67 cm^{-1}

Number of atoms: 48

charge=+1, multiplicity=1

C	-4.135902	3.884298	-0.593697
C	-4.441678	2.530408	-0.386301
C	-3.477150	1.673217	0.120650
C	-2.182196	2.171752	0.430289
C	-1.884853	3.544532	0.212779
C	-2.861908	4.388953	-0.293365
C	-1.219123	1.320923	0.963544
C	-0.370681	0.559358	1.541012
P	0.583855	-0.481659	0.237423
N	2.133126	-0.349631	0.636239
C	3.140105	0.408883	0.265957
N	3.210899	1.753525	0.010644
C	4.525243	2.102387	-0.282182
C	5.273656	0.971347	-0.208855
N	4.419003	-0.065605	0.132730
C	2.100570	2.683275	0.131816
C	4.785227	-1.460357	0.303002
S	-0.074239	0.280714	-1.493698
N	0.113027	-1.978368	0.643288
C	-0.929314	-2.643813	0.184799
N	-2.017760	-3.013495	0.931415
C	-2.875425	-3.785001	0.162365
C	-2.312326	-3.901242	-1.070585
N	-1.114820	-3.202466	-1.050991
C	-2.201397	-2.636037	2.319873
C	-0.158974	-3.145838	-2.146439
H	-0.192877	0.407633	2.609421
H	-3.687597	0.613641	0.272683
H	-0.887086	3.917420	0.447707
H	-3.801879	-4.190458	0.558080
H	-2.654318	-4.429455	-1.955634
H	4.807517	3.123983	-0.517537
H	6.335265	0.813640	-0.373878
H	-5.437008	2.153009	-0.627897
H	-2.640007	5.444633	-0.459837
H	-4.899660	4.553844	-0.995847
H	2.462387	3.689468	-0.112636
H	1.293585	2.400773	-0.558582
H	1.716750	2.683277	1.163271
H	5.621548	-1.547112	1.011363
H	3.909236	-1.988581	0.699186
H	5.076324	-1.905532	-0.660589
H	-2.657777	-3.468107	2.872689
H	-1.213562	-2.411415	2.742084
H	-2.842819	-1.745261	2.410337

H	-0.106457	-4.131964	-2.628443
H	-0.442279	-2.375125	-2.874890
H	0.823147	-2.884853	-1.734455

Closed form (CFa)

Electronic energy = -1766.19857172

Thermal correction to enthalpy = 0.413755

Entropy correction = 0.078921

Lowest frequency = 15.08 cm^{-1}

Number of atoms: 48

charge=+1, multiplicity=1

C	3.955793	4.174892	-0.252320
C	3.740705	3.509916	0.957559
C	2.784835	2.497690	1.037091
C	2.029890	2.135415	-0.093813
C	2.256570	2.814377	-1.309100
C	3.210949	3.824249	-1.385455
C	1.024708	1.077907	-0.015840
C	0.188982	0.585532	-0.972963
P	-0.676971	-0.674016	-0.033253
N	-2.245325	-0.541539	0.138913
C	-3.190175	0.372024	0.045641
N	-3.205131	1.695813	0.391274
C	-4.467993	2.224476	0.139788
C	-5.239187	1.221993	-0.353636
N	-4.448878	0.083430	-0.406894
C	-2.095502	2.445055	0.959808
C	-4.866954	-1.229544	-0.869418
S	0.685154	0.198773	1.525427
N	-0.309197	-2.209163	-0.289389
C	0.892725	-2.763422	-0.193934
N	1.815981	-2.904317	-1.188855
C	2.906678	-3.618330	-0.713343
C	2.652358	-3.922869	0.587744
N	1.406872	-3.398014	0.897896
C	1.656545	-2.366051	-2.528148
C	0.740492	-3.464303	2.187011
H	0.100361	0.877569	-2.019705
H	2.619171	1.980349	1.984706
H	1.681298	2.549189	-2.198007
H	3.760736	-3.853678	-1.341360
H	3.244082	-4.471908	1.314162
H	-4.702267	3.264787	0.344284
H	-6.279118	1.216476	-0.666395
H	4.319395	3.780716	1.842985
H	3.377714	4.343437	-2.331567
H	4.704039	4.968022	-0.315104
H	-2.489944	3.177669	1.676123
H	-1.422370	1.762132	1.491969
H	-1.527382	2.969847	0.177780
H	-5.103672	-1.202430	-1.943566
H	-4.038248	-1.926252	-0.694626
H	-5.752679	-1.562296	-0.309469
H	2.049374	-3.081825	-3.262617
H	0.585765	-2.209499	-2.708460
H	2.184955	-1.406298	-2.630702
H	0.872413	-4.464891	2.620100
H	1.147799	-2.706887	2.873197
H	-0.328059	-3.271886	2.027597

Second transition state (TS2a)

Electronic energy = -1766.17186241

Thermal correction to enthalpy = 0.412573

Entropy correction = 0.077788

Lowest frequency = -58.16 cm^{-1}

Number of atoms: 48

charge=+1, multiplicity=1

C	3.596562	-4.349156	0.436549
C	3.671687	-3.705903	-0.803846
C	2.874541	-2.594372	-1.066446
C	1.977974	-2.108235	-0.095049
C	1.911332	-2.767346	1.149835
C	2.717116	-3.874118	1.414339
C	1.137974	-0.918548	-0.370300
C	-0.064231	-0.759358	0.366989
P	-0.889934	0.727282	0.323505
N	-2.470514	0.726623	0.469746
C	-3.344831	-0.190233	0.066906
N	-3.338647	-0.989840	-1.043395
C	-4.518202	-1.725884	-1.077880
C	-5.252151	-1.374142	0.009885
N	-4.524578	-0.421501	0.707027
C	-2.302782	-1.037123	-2.066623
C	-4.931088	0.239967	1.937875
S	1.582716	0.216097	-1.545368
N	-0.300569	2.182605	0.481023
C	0.966241	2.593575	0.388999
N	2.035268	2.288381	1.171897
C	3.137611	3.004555	0.733765
C	2.734056	3.764288	-0.321529
N	1.388137	3.512257	-0.515314
C	2.032682	1.317591	2.253297
C	0.545266	4.023986	-1.584383
H	-0.567151	-1.585420	0.877711
H	2.927927	-2.084930	-2.029830
H	1.244727	-2.391849	1.928487

H	4.108213	2.915285	1.212070
H	3.287077	4.460057	-0.945248
H	-4.729674	-2.428842	-1.878042
H	-6.226889	-1.714412	0.346641
H	4.355799	-4.075526	-1.570755
H	2.663008	-4.365762	2.388133
H	4.224595	-5.219007	0.641752
H	-2.766935	-1.314754	-3.021517
H	-1.833550	-0.050110	-2.179483
H	-1.516467	-1.759284	-1.807452
H	-4.832377	-0.441482	2.796251
H	-4.281580	1.110876	2.086750
H	-5.975444	0.568954	1.850811
H	2.750224	1.633384	3.021266
H	1.030148	1.275387	2.696104
H	2.305854	0.321596	1.875674
H	0.978515	4.955926	-1.967103
H	0.476819	3.281863	-2.393743
H	-0.459496	4.214324	-1.186339

Open form (OFa)

Electronic energy = -1766.17300746

Thermal correction to enthalpy = 0.413715

Entropy correction = 0.079563

Lowest frequency = 14.66 cm^{-1}

Number of atoms: 48

charge=+1, multiplicity=1

C	-1.884610	5.341192	0.415844
C	-2.523604	4.683096	-0.640899
C	-2.281656	3.331685	-0.874559
C	-1.381702	2.612254	-0.064784
C	-0.742626	3.288483	0.994177
C	-0.998360	4.637914	1.236719
C	-1.133083	1.167556	-0.315939
C	0.151021	0.669662	0.077993
P	0.651029	-0.938838	0.126476
N	2.199436	-1.252468	0.286929
C	3.266489	-0.526281	-0.021148
N	3.526740	0.207333	-1.145449
C	4.816175	0.723346	-1.065391
C	5.351216	0.300526	0.109547
N	4.391478	-0.474382	0.745392
C	2.633142	0.335962	-2.286843
C	4.535897	-1.134843	2.033643
S	-2.308311	0.214433	-1.041628
N	-0.212221	-2.252106	0.140065
C	-1.524434	-2.463606	0.262669
N	-2.348669	-2.125449	1.288356
C	-3.614867	-2.635856	1.038861
C	-3.553878	-3.307459	-0.142479
N	-2.250529	-3.210231	-0.603153
C	-1.967880	-1.275735	2.402034
C	-1.740511	-3.655549	-1.888433
H	0.955410	1.367162	0.328173
H	-2.781951	2.809032	-1.691016
H	-0.068436	2.746014	1.659777
H	-4.443119	-2.478113	1.722708
H	-4.320028	-3.847566	-0.690234
H	5.236897	1.338806	-1.854915
H	6.328388	0.480692	0.547711
H	-3.215552	5.228639	-1.286215
H	-0.509240	5.141600	2.073428
H	-2.080157	6.399746	0.601268
H	3.224049	0.624170	-3.164839
H	2.153996	-0.632536	-2.489372
H	1.852111	1.087560	-2.104260
H	4.494194	-0.401704	2.853228
H	3.713591	-1.852191	2.141604
H	5.495603	-1.668182	2.069543
H	-2.692107	-1.407912	3.214893
H	-0.972455	-1.567447	2.763775
H	-1.955828	-0.220719	2.089735
H	-2.393365	-4.444737	-2.280278
H	-1.715171	-2.807139	-2.589730
H	-0.723306	-4.046498	-1.757130

Reaction of $[(R^{Me})_2PS]^+$ with ethoxyacetylene

Ethoxyacetylene

Electronic energy = -231.005748969

Thermal correction to enthalpy = 0.095999

Entropy correction = 0.035671

Lowest frequency = 66.09 cm^{-1}

Number of atoms: 11

charge=0, multiplicity=1

C	-1.358299	-0.176876	-0.000088
C	-2.528032	0.139494	0.000010
H	-3.566220	0.404352	-0.000205
O	-0.112821	-0.537845	0.000110
C	0.864786	0.529256	0.000065
H	0.697703	1.155004	-0.892993
H	0.697837	1.154906	0.893220
C	2.238753	-0.099163	-0.000079
H	2.379956	-0.726947	-0.892764
H	3.009962	0.686263	-0.000073

H	2.380079	-0.727081	0.892493

Reactant Complex (RC0b)

Electronic energy = -1688.96585652

Thermal correction to enthalpy = 0.391765

Entropy correction = 0.081927

Lowest frequency = 10.62 cm^{-1}

Number of atoms: 45

charge=+1, multiplicity=1

C	0.320548	2.430764	0.840660
C	0.085917	1.702413	1.787821
P	-0.021358	-0.649001	0.065574
N	1.278592	-1.184114	0.800678
C	2.548466	-1.011365	0.433858
N	3.191057	-1.505263	-0.661634
C	4.533056	-1.158246	-0.599587
C	4.716843	-0.453659	0.549588
N	3.487824	-0.375613	1.184672
C	2.569863	-2.319708	-1.695300
C	3.208924	0.286310	2.450210
N	-1.322617	-1.098834	0.859553
C	-2.588762	-0.954190	0.471746
N	-3.529707	-0.250532	1.157294
C	-4.757477	-0.384893	0.527678
C	-4.569920	-1.189785	-0.552990
N	-3.227899	-1.542355	-0.578240
C	-3.244727	0.525197	2.354339
C	-2.597617	-2.441723	-1.532866
H	-0.096421	1.164775	2.696090
H	-5.654897	0.096889	0.904061
H	-5.272947	-1.548749	-1.298614
H	5.238325	-1.449496	-1.372113
H	5.612654	-0.006895	0.970388
H	3.338165	-2.953282	-2.155798
H	1.807355	-2.960550	-1.232886
H	2.091359	-1.687592	-2.455800
H	2.925687	1.336021	2.288037
H	2.374867	-0.235009	2.935672
H	4.101100	0.232173	3.086872
H	-4.179066	0.683894	2.906461
H	-2.538282	-0.035823	2.979851
H	-2.796786	1.495222	2.093712
H	-3.370425	-3.074953	-1.985704
H	-2.067951	-1.874303	-2.310749
H	-1.878247	-3.079712	-1.001397
O	0.612063	3.141215	-0.182550
C	-0.494503	3.672896	-0.982200
H	-1.186505	2.840964	-1.181372
H	-1.003440	4.436533	-0.372862
S	-0.049232	0.130946	-1.715486
C	0.090790	4.240706	-2.250036
H	0.595984	3.453432	-2.828609
H	-0.713649	4.667777	-2.867845
H	0.815538	5.037512	-2.026595

First transition state (TS0b)

Electronic energy = -1688.96459986

Thermal correction to enthalpy = 0.390304

Entropy correction = 0.079708

Lowest frequency = -108.05 cm^{-1}

Number of atoms: 45

charge=+1, multiplicity=1

C	0.322279	2.363695	0.910140
C	0.087274	1.364266	1.607410
P	-0.017907	-0.466618	0.155616
N	1.268686	-1.199378	0.757185
C	2.533689	-1.038329	0.389624
N	3.144358	-1.461893	-0.755112
C	4.500027	-1.175922	-0.687840
C	4.730844	-0.578454	0.512267
N	3.514553	-0.503381	1.172930
C	2.476964	-2.162903	-1.841305
C	3.281401	0.053034	2.494528
N	-1.326764	-1.073009	0.849245
C	-2.580681	-0.968198	0.431645
N	-3.585750	-0.359369	1.124890
C	-4.784814	-0.522894	0.447659
C	-4.517719	-1.248515	-0.671589
N	-3.158093	-1.525008	-0.672698
C	-3.381214	0.349642	2.375654
C	-2.453624	-2.339820	-1.650550
H	-0.105090	0.971091	2.593458
H	-5.720174	-0.117360	0.821525
H	-5.176719	-1.600072	-1.459770
H	5.181849	-1.430127	-1.493826
H	5.651534	-0.207829	0.952889
H	3.189878	-2.853307	-2.310355
H	1.638134	-2.735075	-1.424777
H	2.084280	-1.453566	-2.582129
H	3.116011	1.139421	2.441472

H	2.387347	-0.425747	2.912841
H	4.147228	-0.156822	3.136139
H	-4.322403	0.365785	2.939377
H	-2.613999	-0.178518	2.956780
H	-3.044784	1.381808	2.194305
H	-3.164694	-3.039271	-2.108126
H	-1.985785	-1.709804	-2.419250
H	-1.669012	-2.908722	-1.133766
O	0.603498	3.213687	0.030469
C	-0.490005	3.798622	-0.794519
H	-1.217328	2.992922	-0.960959
H	-0.931359	4.600601	-0.185177
S	-0.059104	0.318774	-1.642904
C	0.128922	4.295360	-2.072712
H	0.574933	3.461435	-2.633187
H	-0.653880	4.757022	-2.693960
H	0.901328	5.052306	-1.872577

Reactant Complex (RCb)

Electronic energy = -1688.96477622

Thermal correction to enthalpy = 0.391082

Entropy correction = 0.080518

Lowest frequency = 12.31 cm^{-1}

Number of atoms: 45

charge=+1, multiplicity=1

C	0.402022	2.372108	0.919818
C	0.122302	1.321403	1.547487
P	-0.038328	-0.352434	0.225149
N	1.222477	-1.185729	0.767103
C	2.486017	-1.082845	0.385379
N	3.058731	-1.511471	-0.778675
C	4.429117	-1.303169	-0.725186
C	4.710368	-0.748384	0.484436
N	3.509195	-0.620620	1.165013
C	2.340509	-2.152969	-1.868897
C	3.326488	-0.088264	2.503189
N	-1.366226	-0.963052	0.894980
C	-2.602041	-0.925020	0.426278
N	-3.664298	-0.348780	1.064090
C	-4.826043	-0.582602	0.343278
C	-4.480061	-1.318094	-0.747251
N	-3.110361	-1.531671	-0.687728
C	-3.545442	0.396979	2.303105
C	-2.333821	-2.344468	-1.610717
H	-0.090450	1.024338	2.567606
H	-5.793702	-0.213636	0.669806
H	-5.089612	-1.716087	-1.552940
H	5.084431	-1.575741	-1.547027
H	5.656864	-0.441287	0.919216
H	2.999286	-2.891860	-2.343775
H	1.461327	-2.661522	-1.453592
H	1.998613	-1.411871	-2.603528
H	3.264713	1.010519	2.487324
H	2.390455	-0.496327	2.904692
H	4.166244	-0.397544	3.139675
H	-4.483194	0.317104	2.867851
H	-2.725260	-0.035271	2.890818
H	-3.327346	1.458689	2.108652
H	-2.993161	-3.096270	-2.063150
H	-1.870121	-1.720748	-2.386829
H	-1.540250	-2.852226	-1.045778
O	0.713339	3.254318	0.101134
C	-0.345845	3.894406	-0.754134
H	-1.121947	3.130421	-0.890556
H	-0.718119	4.741290	-0.161195
S	-0.081514	0.409139	-1.595542
C	0.311189	4.301063	-2.043131
H	0.694740	3.417748	-2.572843
H	-0.439289	4.794195	-2.680386
H	1.133032	5.010215	-1.867074

First transition state (TS1b)

Electronic energy = -1688.96208076

Thermal correction to enthalpy = 0.389907

Entropy correction = 0.078412

Lowest frequency = -249.64 cm^{-1}

Number of atoms: 45

charge=+1, multiplicity=1

C	0.429231	2.321413	0.781631
C	0.260545	1.260219	1.478769
P	-0.032630	-0.245191	0.314686
N	1.190337	-1.244536	0.636397
C	2.461362	-1.152365	0.289086
N	3.036776	-1.407126	-0.925480
C	4.415975	-1.295322	-0.820450
C	4.702888	-0.974492	0.469303
N	3.496180	-0.891530	1.147670
C	2.315247	-1.799042	-2.125462
C	3.317245	-0.594527	2.556503
N	-1.380580	-0.864530	0.945769

C	-2.567034	-1.007767	0.391277
N	-3.745377	-0.576867	0.939053
C	-4.808023	-0.974238	0.139879
C	-4.286303	-1.660422	-0.910794
N	-2.907454	-1.685652	-0.748355
C	-3.834050	0.184327	2.169888
C	-1.968658	-2.405646	-1.593426
H	0.215731	1.174706	2.567226
H	-5.838169	-0.738604	0.389634
H	-4.774403	-2.139778	-1.753990
H	5.071791	-1.461002	-1.669950
H	5.656082	-0.805335	0.961301
H	2.890886	-2.570243	-2.655416
H	1.342194	-2.204831	-1.826093
H	2.138441	-0.934380	-2.778466
H	3.383965	0.488229	2.745680
H	2.324160	-0.956506	2.852279
H	4.086387	-1.112006	3.145909
H	-4.618137	-0.235242	2.815711
H	-2.865043	0.112071	2.678844
H	-4.061952	1.242016	1.964683
H	-2.472430	-3.283721	-2.018817
H	-1.583278	-1.760283	-2.394171
H	-1.127295	-2.739915	-0.970947
O	0.656322	3.371491	0.176051
C	-0.464798	4.091333	-0.541541
H	-0.971395	3.293252	-1.104088
H	-1.111062	4.484973	0.255027
S	-0.125948	0.652368	-1.480956
C	0.163323	5.147781	-1.403180
H	0.830981	4.697435	-2.151512
H	-0.639260	5.681644	-1.935591
H	0.724972	5.879746	-0.804916

Closed form (CFb)

Electronic energy = -1689.01914569

Thermal correction to enthalpy = 0.392668

Entropy correction = 0.078256

Lowest frequency = 13.09 cm^{-1}

Number of atoms: 45

charge=+1, multiplicity=1

C	-0.000076	1.701082	-0.444539
C	0.000193	0.620242	-1.281950
P	-0.000012	-0.692763	-0.082662
N	-1.298620	-1.600274	0.075800
C	-2.566640	-1.214895	0.032941
N	-3.359116	-0.930213	1.105134
C	-4.649272	-0.661821	0.671854
C	-4.655882	-0.790991	-0.682083
N	-3.367492	-1.133915	-1.068846
C	-2.883798	-0.908275	2.478368
C	-2.914340	-1.379259	-2.426709
N	1.298608	-1.600163	0.076196
C	2.566662	-1.214939	0.033131
N	3.367445	-1.134320	-1.068737
C	4.655898	-0.791429	-0.682153
C	4.649396	-0.661916	0.671752
N	3.359245	-0.930071	1.105191
C	2.914195	-1.379982	-2.426508
C	2.883968	-0.907685	2.478439
H	0.000509	0.619537	-2.371340
H	5.462839	-0.678874	-1.400056
H	5.449482	-0.411786	1.362073
H	-5.449284	-0.411786	1.362296
H	-5.462862	-0.678166	-1.399902
H	-3.681955	-1.257314	3.146314
H	-2.021601	-1.583653	2.551882
H	-2.570869	0.106182	2.765949
H	-2.697963	-0.433390	-2.945582
H	-2.000647	-1.984867	-2.379003
H	-3.686650	-1.931429	-2.978720
H	3.686376	-1.932464	-2.978387
H	2.000390	-1.985405	-2.378591
H	2.697982	-0.434228	-2.945661
H	3.682547	-1.255426	3.146550
H	2.569987	0.106644	2.765333
H	2.022478	-1.583904	2.552515
O	-0.000052	2.960848	-0.807775
C	-0.000703	4.012637	0.186236
H	-0.895305	3.890549	0.818356
H	0.891896	3.889250	0.820932
S	-0.000496	1.146697	1.270197
C	0.001290	5.339054	-0.536481
H	-0.891021	5.441226	-1.171607
H	0.000759	6.158577	0.197629
H	0.895622	5.439992	-1.168957

Second transition state (TS2b)

Electronic energy = -1689.00273929

Thermal correction to enthalpy = 0.391452

Entropy correction = 0.076600

Lowest frequency = -97.49 cm^{-1}

Number of atoms: 45

charge=+1, multiplicity=1

C	0.925661	1.586720	0.066182
C	-0.122096	0.970807	0.791786
P	-0.510222	-0.610543	0.294050
N	0.452451	-1.865477	0.231379
C	1.785567	-1.861519	0.181175
N	2.503897	-2.381957	-0.846694
C	3.853741	-2.252350	-0.577426
C	3.966128	-1.664652	0.645543
N	2.682049	-1.433070	1.111627
C	1.910566	-2.889656	-2.073846
C	2.342301	-0.810919	2.381419
N	-2.026713	-1.070150	0.259090
C	-3.122922	-0.355155	0.022077
N	-4.330243	-0.650012	0.579087
C	-5.292108	0.219150	0.088026
C	-4.672975	1.050153	-0.790844
N	-3.330398	0.691212	-0.832557
C	-4.545754	-1.714314	1.548017
C	-2.333791	1.318178	-1.690359
H	-0.698754	1.482966	1.566480
H	-6.330083	0.167216	0.403005
H	-5.066341	1.860869	-1.396744
H	4.616132	-2.592522	-1.271803
H	4.844929	-1.405022	1.228079
H	2.513682	-3.725799	-2.450265
H	0.894276	-3.234102	-1.847867
H	1.861926	-2.092582	-2.830245
H	2.282289	0.281936	2.277011
H	1.369447	-1.190443	2.716639
H	3.105574	-1.074646	3.124803
H	-5.466361	-2.257690	1.296352
H	-3.688983	-2.397266	1.500806
H	-4.629602	-1.303365	2.565422
H	-1.843240	2.157185	-1.178324
H	-1.557676	0.593659	-1.971104
H	-2.829975	1.673129	-2.602766
O	1.376141	2.721289	0.585649
C	2.388612	3.487831	-0.099531
H	3.302102	2.876027	-0.164895
H	2.047085	3.673821	-1.130548
S	1.508578	0.904745	-1.380251
C	2.605009	4.765725	0.678269
H	2.939747	4.552966	1.704771
H	3.376696	5.373759	0.182672
H	1.679136	5.358001	0.730157

Open form (OFb)

Electronic energy = -1689.00900360

Thermal correction to enthalpy = 0.392747

Entropy correction = 0.078599

Lowest frequency = 23.46 cm^{-1}

Number of atoms: 45

charge=+1, multiplicity=1

C	-0.917792	1.831830	-0.081602
C	0.260125	1.024432	-0.261067
P	0.430560	-0.627405	-0.043575
N	-0.668702	-1.717174	0.212358
C	-1.988947	-1.768992	0.031040
N	-2.880765	-2.089810	0.998164
C	-4.153427	-2.136124	0.447851
C	-4.027843	-1.874253	-0.880676
N	-2.678712	-1.667085	-1.133589
C	-2.536580	-2.168498	2.406786
C	-2.086403	-1.247504	-2.389364
N	1.885009	-1.259539	-0.110871
C	3.081451	-0.708734	0.050314
N	4.157835	-0.985185	-0.737118
C	5.273014	-0.317507	-0.250078
C	4.882121	0.371102	0.853748
N	3.525679	0.123882	1.039120
C	4.116158	-1.845905	-1.909192
C	2.722625	0.585802	2.160626
H	1.184089	1.539220	-0.541006
H	6.244070	-0.392516	-0.730418
H	5.445978	1.009030	1.527722
H	-5.031467	-2.360111	1.045791
H	-4.774547	-1.825034	-1.667151
H	-3.313079	-2.733395	2.936797
H	-1.571441	-2.680277	2.512700
H	-2.458686	-1.154368	2.828844
H	-1.869947	-0.168488	-2.360236
H	-1.159179	-1.809784	-2.567599
H	-2.789812	-1.456300	-3.204321
H	4.959235	-2.549612	-1.880885
H	3.172571	-2.404047	-1.887136
H	4.168918	-1.248760	-2.831987
H	2.073941	1.424947	1.871072
H	2.096357	-0.240135	2.526938
H	3.394190	0.901843	2.968176
O	-0.638815	3.112376	-0.319981

C	-1.661811	4.119791	-0.198909
H	-2.489394	3.859674	-0.877955
H	-2.063952	4.088060	0.826187
S	-2.430297	1.285525	0.377065
C	-1.033504	5.453419	-0.534944
H	-0.638566	5.457483	-1.562064
H	-1.789671	6.248614	-0.453113
H	-0.210282	5.688837	0.156433

Reaction of $[(R^{Me})_2PS]^+$ with (trifluoromethyl)acetylene

(Trifluoromethyl)acetylene

Electronic energy = -414.060922299

Thermal correction to enthalpy = 0.039941

Entropy correction = 0.035257

Lowest frequency = 184.11 cm^{-1}

Number of atoms: 11

charge=0, multiplicity=1

C	1.130037	0.000063	-0.000174
C	2.337224	0.000190	0.000114
H	3.412082	0.000046	-0.000139
C	-0.338737	0.000072	0.000058
F	-0.821761	0.647448	-1.069638
F	-0.821722	0.602537	1.095577
F	-0.821320	-1.250207	-0.025924

Reactant complex (RCc)

Electronic energy = -1872.01489265

Thermal correction to enthalpy = 0.335778

Entropy correction = 0.080407

Lowest frequency = 15.74 cm^{-1}

Number of atoms: 41

charge=+1, multiplicity=1

C	0.557522	2.401075	1.012353
C	-0.133126	2.074541	1.947508
P	-0.344498	-0.723896	-0.168920
N	0.884498	-1.151632	0.738850
C	2.151366	-1.370305	0.379890
N	2.642344	-2.233569	-0.554392
C	4.029846	-2.194083	-0.527767
C	4.392116	-1.308920	0.438193
N	3.226642	-0.813508	0.999942
C	1.844409	-3.122870	-1.386223
C	3.151257	0.159388	2.077794
N	-1.659443	-0.644522	0.718606
C	-2.917172	-0.390184	0.353085
N	-3.967000	-1.179941	0.715197
C	-5.147965	-0.623079	0.252016
C	-4.822329	0.528179	-0.393430
N	-3.442707	0.671128	-0.323003
C	-3.837012	-2.428635	1.450846
C	-2.704596	1.807452	-0.860012
H	-0.746646	1.774839	2.778695
H	-6.112946	-1.090501	0.424564
H	-5.448128	1.263416	-0.890442
H	4.633511	-2.802983	-1.193999
H	5.373966	-0.989715	0.774820
H	2.457294	-3.990418	-1.660668
H	0.975888	-3.471283	-0.811213
H	1.491424	-2.606614	-2.289112
H	3.477130	1.147386	1.724394
H	2.109131	0.219010	2.408986
H	3.788019	-0.164428	2.912546
H	-2.840407	-2.453396	1.907606
H	-4.604893	-2.475270	2.234751
H	-3.951507	-3.290144	0.775787
H	-3.312298	2.713227	-0.735696
H	-1.769031	1.928894	-0.306273
H	-2.465808	1.652869	-1.920704
C	1.389622	2.758052	-0.145060
F	2.172202	3.804339	0.115387
F	0.629974	3.048842	-1.207315
F	2.189205	1.723746	-0.482846
S	-0.270226	-0.373634	-2.068053

First transition state (TS1c)

Electronic energy = -1871.97336998

Thermal correction to enthalpy = 0.333528

Entropy correction = 0.078199

Lowest frequency = -451.53 cm^{-1}

Number of atoms: 41

charge=+1, multiplicity=1

C	-0.160658	1.841479	0.810049
C	-0.241867	0.809839	1.503742
P	0.105312	-0.558115	-0.131813
N	1.321573	-1.426915	0.438161
C	2.609520	-1.194669	0.174288

N	3.289899	-1.461509	-0.973615
C	4.629768	-1.155164	-0.793246
C	4.775731	-0.701113	0.481415
N	3.523395	-0.737843	1.073446
C	2.702904	-2.013889	-2.187040
C	3.189083	-0.320430	2.424422
N	-1.238652	-1.387293	0.005263
C	-2.547279	-1.234647	-0.021467
N	-3.388782	-1.920692	0.815032
C	-4.700817	-1.604011	0.519160
C	-4.676990	-0.712633	-0.510497
N	-3.350491	-0.492206	-0.842886
C	-2.943235	-2.855257	1.836752
C	-2.892498	0.368210	-1.926952
H	-0.420330	0.367596	2.477842
H	-5.537382	-2.038485	1.058663
H	-5.488782	-0.221181	-1.038587
H	5.362471	-1.293456	-1.582697
H	5.658010	-0.361312	1.015617
H	3.432473	-2.681325	-2.663874
H	1.809551	-2.589561	-1.914682
H	2.413127	-1.210429	-2.877369
H	2.937504	0.750687	2.452891
H	2.324123	-0.905058	2.762750
H	4.043284	-0.512300	3.086190
H	-1.851338	-2.930197	1.769062
H	-3.232022	-2.496066	2.835462
H	-3.389839	-3.844623	1.662825
H	-3.730127	0.533986	-2.615358
H	-2.531429	1.330952	-1.541718
H	-2.065328	-0.117110	-2.458220
C	-0.236489	3.248203	0.324306
F	-0.514878	4.020137	1.382868
F	-1.218314	3.395600	-0.569889
F	0.902478	3.660982	-0.213792
S	0.501323	0.751803	-1.578855

Closed form (CFc)

Electronic energy = -1872.05524825

Thermal correction to enthalpy = 0.336575

Entropy correction = 0.077161

Lowest frequency = 15.38 cm^{-1}

Number of atoms: 41

charge=+1, multiplicity=1

C	-0.334054	1.764605	-0.105252
C	0.093115	0.889013	-1.036847
P	0.099411	-0.611977	-0.020180
N	-1.037998	-1.699535	-0.251897
C	-2.353924	-1.538112	-0.136842
N	-3.104407	-1.789169	0.971124
C	-4.444523	-1.585328	0.678492
C	-4.521036	-1.215546	-0.628451
N	-3.225543	-1.190512	-1.124695
C	-2.554914	-2.177261	2.259842
C	-2.828525	-0.838484	-2.477838
N	1.499568	-1.298666	0.214072
C	2.783037	-1.006943	0.075463
N	3.690965	-1.891201	-0.432051
C	4.957738	-1.326771	-0.403635
C	4.832929	-0.084690	0.131263
N	3.488054	0.107305	0.431651
C	3.355566	-3.218749	-0.923109
C	2.944067	1.297998	1.068292
H	0.364298	1.077426	-2.076322
H	5.833699	-1.859391	-0.761994
H	5.577966	0.678802	0.334060
H	-5.224779	-1.726430	1.420561
H	-5.380755	-0.976011	-1.247201
H	-3.185685	-2.955323	2.709939
H	-1.545588	-2.575234	2.096099
H	-2.501986	-1.310330	2.935248
H	-2.769311	0.253825	-2.597321
H	-1.843106	-1.279256	-2.673079
H	-3.556424	-1.245178	-3.192182
H	3.977780	-3.972334	-0.420072
H	2.298755	-3.405448	-0.697996
H	3.516304	-3.279020	-2.009758
H	3.674089	1.680981	1.793101
H	2.721013	2.081854	0.330339
H	2.021824	1.040574	1.602020
C	-0.470248	3.258346	-0.270033
F	-0.419812	3.596267	-1.562644
F	0.540049	3.877241	0.362475
F	-1.617690	3.703479	0.243246
S	-0.620126	0.961328	1.457754

Second transition state (TS2c)

Electronic energy = -1872.03443138

Thermal correction to enthalpy = 0.335405

Entropy correction = 0.075370

Lowest frequency = -77.61 cm^{-1}

Number of atoms: 41

charge=+1, multiplicity=1

C	0.823354	1.555753	-0.195807
C	-0.181225	0.949119	0.562789
P	-0.467525	-0.721109	0.271113
N	0.597219	-1.879807	0.310944
C	1.930634	-1.794704	0.234917
N	2.658949	-2.383444	-0.745956
C	4.002638	-2.149603	-0.521643
C	4.098746	-1.426379	0.628618
N	2.811096	-1.216101	1.093343
C	2.068970	-3.052055	-1.896284
C	2.461931	-0.473572	2.296155
N	-1.950540	-1.263600	0.296730
C	-3.093522	-0.633002	0.025014
N	-4.272635	-0.957766	0.620121
C	-5.289274	-0.184060	0.083687
C	-4.730829	0.615880	-0.862858
N	-3.370190	0.334570	-0.899308
C	-4.415194	-1.959416	1.667932
C	-2.425569	0.951101	-1.822881
H	-0.830119	1.486601	1.259086
H	-6.318514	-0.272093	0.418985
H	-5.178816	1.356404	-1.518810
H	4.772886	-2.516170	-1.193631
H	4.968907	-1.049849	1.157956
H	2.795509	-3.762565	-2.308830
H	1.167600	-3.586147	-1.570771
H	1.793148	-2.311745	-2.661683
H	2.402310	0.601904	2.080529
H	1.491992	-0.827059	2.665830
H	3.224300	-0.657951	3.063889
H	-5.307790	-2.567964	1.471315
H	-3.523281	-2.597220	1.655978
H	-4.508550	-1.478956	2.653344
H	-2.957713	1.209127	-2.747280
H	-1.975014	1.852801	-1.386137
H	-1.618033	0.248087	-2.069564
C	1.237178	2.960025	0.256357
F	2.144223	2.854996	1.252072
F	0.182324	3.635841	0.748355
F	1.772901	3.683943	-0.714069
S	1.592168	0.845307	-1.505076

Open form (OFc)

Electronic energy = -1872.03684165

Thermal correction to enthalpy = 0.336598

Entropy correction = 0.077151

Lowest frequency = 16.83 cm^{-1}

Number of atoms: 41

charge=+1, multiplicity=1

C	0.929324	1.668905	-0.228109
C	-0.228714	0.944928	0.149059
P	-0.432276	-0.730658	0.041733
N	0.691932	-1.820780	-0.056221
C	2.017085	-1.751736	0.108486
N	2.904537	-2.295114	-0.757802
C	4.187539	-2.129910	-0.262697
C	4.077999	-1.501361	0.938795
N	2.727417	-1.276852	1.166125
C	2.532538	-2.814727	-2.063781
C	2.160507	-0.591449	2.316199
N	-1.884940	-1.345085	0.143350
C	-3.085771	-0.800203	-0.037743
N	-4.170961	-1.121086	0.717973
C	-5.283540	-0.444948	0.241138
C	-4.881474	0.291287	-0.828052
N	-3.520175	0.066546	-1.000676
C	-4.140007	-2.026873	1.857628
C	-2.714507	0.601692	-2.089744
H	-1.104684	1.478389	0.527548
H	-6.261582	-0.548238	0.701851
H	-5.440961	0.949403	-1.486205
H	5.063259	-2.475429	-0.803719
H	4.838439	-1.195420	1.650765
H	3.330816	-3.471926	-2.429092
H	1.597773	-3.382072	-1.971098
H	2.382963	-1.981690	-2.767580
H	1.959099	0.463759	2.080896
H	1.228456	-1.088624	2.617317
H	2.872524	-0.649150	3.148368
H	-4.997357	-2.711145	1.804733
H	-3.207318	-2.601519	1.812187
H	-4.178887	-1.463114	2.801739
H	-3.380954	0.872566	-2.917844
H	-2.143889	1.485478	-1.771156
H	-2.014299	-0.167925	-2.442690
C	0.900207	3.128561	0.258103
F	1.242202	3.155344	1.562332
F	-0.342677	3.645341	0.161263
F	1.723576	3.920735	-0.406097
S	2.266941	1.128459	-1.042776

Reaction of [$(R^{Me})_2PS]^+$ with (dimethylamino)acetylene

(Dimethylamino)acetylene

Electronic energy = -211.161226151

Thermal correction to enthalpy = 0.108084

Entropy correction = 0.036486

Lowest frequency = 151.46 cm^{-1}

Number of atoms: 12

charge=0, multiplicity=1

C	0.995259	0.000000	-0.065631
C	2.204922	0.000000	0.060495
H	3.272784	0.000003	0.149809
N	-0.338541	0.000000	-0.226785
C	-1.053000	1.235072	0.059498
H	-0.476698	2.089906	-0.319438
H	-2.030690	1.220006	-0.447974
H	-1.226662	1.383168	1.144671
C	-1.052998	-1.235073	0.059499
H	-2.030693	-1.220003	-0.447966
H	-0.476701	-2.089906	-0.319448
H	-1.226651	-1.383174	1.144672

Reactant complex (RCd)

Electronic energy = -1669.14415978

Thermal correction to enthalpy = 0.404247

Entropy correction = 0.080504

Lowest frequency = 20.18 cm^{-1}

Number of atoms: 46

charge=+1, multiplicity=1

C	-0.119653	2.776018	-0.893100
C	-0.021044	1.642459	-1.487560
P	-0.031185	0.130134	-0.302800
N	-1.397678	-0.531933	-0.883151
C	-2.391273	-1.094091	-0.231986
N	-2.367935	-1.996648	0.801159
C	-3.667705	-2.372071	1.123251
C	-4.503676	-1.714773	0.279669
N	-3.715516	-0.928430	-0.550898
C	-1.161851	-2.544194	1.398998
C	-4.192334	-0.059959	-1.607731
N	1.231559	-0.742253	-0.820251
C	2.458988	-0.936630	-0.396757
N	3.072237	-2.163991	-0.415622
C	4.380900	-2.047244	0.031084
C	4.590891	-0.738638	0.324411
N	3.407351	-0.056062	0.057519
C	2.408338	-3.387582	-0.823619
C	3.226520	1.381357	0.138736
H	0.006813	1.458723	-2.564056
H	5.045157	-2.903261	0.102475
H	5.474056	-0.230061	0.698710
H	-3.879360	-3.077896	1.920680
H	-5.586043	-1.737098	0.196084
H	-1.385053	-3.543822	1.794524
H	-0.388765	-2.628072	0.623094
H	-0.781520	-1.892109	2.197149
H	-4.821750	0.744526	-1.196775
H	-3.314519	0.373631	-2.101975
H	-4.775851	-0.632224	-2.344356
H	1.516088	-3.110616	-1.398945
H	3.082399	-3.986008	-1.452025
H	2.105946	-3.983809	0.051794
H	4.172955	1.835653	0.457108
H	2.954967	1.781976	-0.850351
H	2.432287	1.621538	0.859962
C	0.882774	4.449897	0.495008
H	1.798731	4.166789	-0.034567
H	0.735046	5.537000	0.437284
H	0.945171	4.129484	1.545855
N	-0.269375	3.770358	-0.122231
C	-1.603818	4.119184	0.402954
H	-2.371039	3.638201	-0.213507
H	-1.669548	3.737811	1.433087
H	-1.726766	5.210551	0.384575
S	-0.034279	0.827338	1.563149

First transition state (TS1d)

Electronic energy = -1669.14072721

Thermal correction to enthalpy = 0.403147

Entropy correction = 0.079314

Lowest frequency = -224.08 cm^{-1}

Number of atoms: 46

charge=+1, multiplicity=1

C	0.034179	2.721102	-0.615524
C	0.038282	1.692823	-1.415164
P	-0.044367	0.152655	-0.406588
N	-1.465632	-0.455674	-0.887598

C	-2.445425	-1.013790	-0.215279
N	-2.416269	-1.850450	0.872623
C	-3.713292	-2.232330	1.203216
C	-4.551071	-1.645699	0.311489
N	-3.768943	-0.895452	-0.557298
C	-1.209997	-2.325449	1.528876
C	-4.251029	-0.106688	-1.673564
N	1.153096	-0.841188	-0.836203
C	2.365461	-1.108385	-0.409599
N	2.875711	-2.380480	-0.344313
C	4.195419	-2.340417	0.082978
C	4.515027	-1.035912	0.280251
N	3.388490	-0.279036	-0.028318
C	2.114189	-3.571236	-0.671203
C	3.324271	1.170874	-0.041325
H	0.032919	1.763416	-2.509567
H	4.789215	-3.240782	0.208830
H	5.441538	-0.576415	0.610679
H	-3.920323	-2.890434	2.041571
H	-5.631716	-1.694667	0.217654
H	-1.425855	-3.286963	2.012874
H	-0.426165	-2.472850	0.773872
H	-0.846847	-1.601797	2.271850
H	-4.950624	0.668228	-1.325334
H	-3.381619	0.365698	-2.146300
H	-4.759098	-0.747110	-2.410299
H	1.236709	-3.260699	-1.252007
H	2.730144	-4.255053	-1.271556
H	1.782442	-4.091329	0.241230
H	4.324155	1.567371	0.174006
H	3.009663	1.521297	-1.035918
H	2.605938	1.525872	0.710891
C	1.182801	4.490372	0.485473
H	2.065143	4.160365	-0.074812
H	1.101637	5.584339	0.441900
H	1.266071	4.157219	1.533700
N	-0.023904	3.894467	-0.103708
C	-1.317194	4.387696	0.395838
H	-2.124070	3.994379	-0.233233
H	-1.453505	4.028641	1.429684
H	-1.317906	5.485152	0.368508
S	0.039381	1.010200	1.439721

Closed form (CFd)

Electronic energy = -1669.17846859

Thermal correction to enthalpy = 0.405159

Entropy correction = 0.079074

Lowest frequency = 18.35 cm^{-1}

Number of atoms: 46

charge=+1, multiplicity=1

C	0.448762	1.944558	0.161935
C	0.000401	0.987413	1.053678
P	-0.130100	-0.415259	-0.016253
N	0.951456	-1.594732	0.061693
C	2.269128	-1.462170	0.071479
N	3.101091	-1.563144	-1.005692
C	4.419730	-1.464134	-0.587896
C	4.405282	-1.309538	0.763359
N	3.076077	-1.309617	1.163006
C	2.640143	-1.731146	-2.372854
C	2.586749	-1.182378	2.523912
N	-1.548343	-1.084192	-0.270574
C	-2.817604	-0.771981	-0.114172
N	-3.754653	-1.685537	0.289434
C	-5.008181	-1.092780	0.317955
C	-4.852470	0.197199	-0.075100
N	-3.500948	0.391426	-0.346920
C	-3.449321	-3.064386	0.631283
C	-2.929468	1.634306	-0.837483
H	-0.200522	1.080595	2.119161
H	-5.898633	-1.641717	0.609582
H	-5.579671	0.994264	-0.196590
H	5.251161	-1.521073	-1.284148
H	5.221362	-1.210494	1.472885
H	3.286600	-2.449480	-2.894794
H	1.613949	-2.118726	-2.338610
H	2.646474	-0.769200	-2.906445
H	2.559697	-0.127812	2.835791
H	1.569844	-1.592497	2.560264
H	3.234832	-1.751433	3.204108
H	-2.394969	-3.243275	0.387681
H	-3.613445	-3.242596	1.704743
H	-4.084027	-3.746693	0.047916
H	-3.652826	2.121285	-1.505010
H	-2.682772	2.312895	-0.007694
H	-2.014797	1.421644	-1.403624
C	0.582513	3.764612	1.725324
H	-0.388037	3.474270	2.155695
H	1.383539	3.389006	2.386621
H	0.635119	4.860404	1.699388
N	0.714015	3.244023	0.371701
C	1.335244	4.102287	-0.626168
H	1.276747	3.631972	-1.615576
H	0.812290	5.070029	-0.670015

H	2.397423	4.289582	-0.390288
S	0.594740	1.181198	-1.461082

Second transition state (TS2d)

Electronic energy = -1669.15769601

Thermal correction to enthalpy = 0.404033

Entropy correction = 0.077963

Lowest frequency = -72.85 cm^{-1}

Number of atoms: 46

charge=+1, multiplicity=1

C	0.854895	1.804840	-0.120705
C	-0.198940	1.131511	0.589463
P	-0.388909	-0.530596	0.345099
N	0.684807	-1.689667	0.393091
C	2.011146	-1.615256	0.288772
N	2.723666	-2.211927	-0.699834
C	4.073037	-1.985840	-0.496303
C	4.191407	-1.260847	0.649741
N	2.911161	-1.045566	1.134455
C	2.110765	-2.865761	-1.844696
C	2.565834	-0.268873	2.311060
N	-1.857621	-1.137240	0.429115
C	-3.003390	-0.590969	0.041698
N	-4.196937	-0.859427	0.642699
C	-5.216411	-0.206704	-0.034942
C	-4.647822	0.463604	-1.070407
N	-3.278394	0.222575	-1.024322
C	-4.347796	-1.704325	1.817129
C	-2.313780	0.717647	-1.996170
H	-0.996470	1.643566	1.133209
H	-6.254120	-0.280579	0.276396
H	-5.092920	1.084753	-1.841851
H	4.830731	-2.362554	-1.176675
H	5.072226	-0.890739	1.165462
H	2.816526	-3.593034	-2.264654
H	1.199990	-3.378453	-1.511034
H	1.843770	-2.118726	-2.607023
H	2.429583	0.789615	2.045186
H	1.631524	-0.655753	2.735949
H	3.366494	-0.365714	3.055408
H	-3.417861	-2.270272	1.949781
H	-4.536450	-1.094996	2.713957
H	-5.184486	-2.399959	1.666072
H	-2.829681	0.878481	-2.951348
H	-1.846969	1.652958	-1.658709
H	-1.513231	-0.018828	-2.149231
C	0.594664	3.703097	1.411095
H	-0.393611	4.143751	1.188034
H	0.479415	2.980738	2.230948
H	1.256313	4.509525	1.754444
N	1.185107	3.065678	0.241295
C	2.111782	3.882476	-0.532116
H	2.285910	3.412879	-1.506787
H	1.687363	4.888850	-0.672613
H	3.079744	3.979307	-0.011604
S	1.625311	0.986596	-1.414813

Open form (OFd)

Electronic energy = -1669.15940153

Thermal correction to enthalpy = 0.405303

Entropy correction = 0.079323

Lowest frequency = 16.32 cm^{-1}

Number of atoms: 46

charge=+1, multiplicity=1

C	-0.869710	1.979674	0.025380
C	0.276471	1.133151	-0.240482
P	0.352433	-0.537663	-0.135775
N	-0.778053	-1.619555	-0.005897
C	-2.108306	-1.581473	-0.098168
N	-2.952175	-1.969846	0.887174
C	-4.257834	-1.892463	0.426592
C	-4.204088	-1.486495	-0.870426
N	-2.865198	-1.315556	-1.192774
C	-2.518611	-2.212724	2.251352
C	-2.334309	-0.770697	-2.427074
N	1.783037	-1.224385	-0.263638
C	2.993581	-0.770574	0.019765
N	4.098668	-1.023579	-0.737850
C	5.219845	-0.485918	-0.119897
C	4.805115	0.099188	1.033424
N	3.427576	-0.080700	1.119321
C	4.077970	-1.751310	-1.996770
C	2.589157	0.292011	2.247339
H	1.248398	1.559576	-0.497039
H	6.211666	-0.568998	-0.554262
H	5.364378	0.620611	1.804441
H	-5.106509	-2.140215	1.056640
H	-4.996303	-1.311980	-1.591786
H	-3.312294	-2.741741	2.792556
H	-1.608451	-2.826361	2.237678
H	-2.303076	-1.252200	2.745052

H	-2.090905	0.294176	-2.289438
H	-1.431229	-1.325238	-2.716760
H	-3.085952	-0.876280	-3.218680
H	3.090411	-2.216121	-2.102291
H	4.256827	-1.070611	-2.842716
H	4.850995	-2.532143	-1.987294
H	3.232077	0.498666	3.111629
H	1.979004	1.177973	2.020294
H	1.919237	-0.542998	2.499004
C	0.447860	3.842053	-0.899290
H	1.324392	3.831736	-0.227698
H	0.699842	3.278545	-1.811040
H	0.260314	4.882036	-1.188928
N	-0.742660	3.301075	-0.254238
C	-1.834108	4.215658	0.072511
H	-2.135120	4.095355	1.123312
H	-1.500571	5.245702	-0.100686
H	-2.723728	4.006078	-0.542810
S	-2.307903	1.365420	0.681702

Oxophosphonium model compound $[(R^{Me})_2PO]^+$

Separated reactant (SRe)

Electronic energy = -1135.01284990

Thermal correction to enthalpy = 0.295388

Entropy correction = 0.065167

Lowest frequency = 22.83 cm^{-1}

Number of atoms: 34

charge=+1, multiplicity=1

P	0.000021	-0.310347	0.000008
N	-1.296453	0.585745	0.009537
C	-2.587960	0.253187	-0.032481
N	-3.229247	-0.679586	-0.791093
C	-4.594467	-0.601410	-0.548310
C	-4.790821	0.387929	0.363575
N	-3.546301	0.914729	0.671536
C	-2.588095	-1.561639	-1.759726
C	-3.278624	1.992649	1.612231
O	0.000201	-1.802916	-0.000085
N	1.296430	0.585868	-0.009229
C	2.587950	0.253320	0.032528
N	3.229309	-0.679115	0.791495
C	4.594494	-0.601165	0.548441
C	4.790748	0.387704	-0.363971
N	3.546198	0.914419	-0.672000
C	2.588165	-1.560687	1.760564
C	3.278437	1.992021	-1.613011
H	5.303320	-1.254040	1.048860
H	5.703439	0.761386	-0.818564
H	-5.303247	-1.254485	-1.048533
H	-5.703568	0.761902	0.817818
H	-3.368798	-2.122045	-2.286895
H	-2.026588	-0.962753	-2.492472
H	-1.899927	-2.252909	-1.256064
H	-3.982042	2.817562	1.435754
H	-3.383680	1.637055	2.648169
H	-2.252215	2.341511	1.447964
H	3.368870	-2.120691	2.288158
H	2.026470	-0.961461	2.492884
H	1.900220	-2.252387	1.257197
H	3.981650	2.817155	-1.436709
H	3.383710	1.636120	-2.648818
H	2.251931	2.340719	-1.448951

Reaction of $[(R^{Me})_2PO]^+$ with ethoxyacetylene

Reactant complex (RCe)

Electronic energy = -1366.04720751

Thermal correction to enthalpy = 0.392684

Entropy correction = 0.077910

Lowest frequency = 14.25 cm^{-1}

Number of atoms: 45

charge=+1, multiplicity=1

C	-2.420383	-1.345617	-1.217301
C	-1.248718	-0.995439	-1.550440
P	-0.318682	-0.509498	0.152712
N	0.899235	-1.556011	0.035888
C	2.191869	-1.356769	0.164070
N	2.869610	-0.612349	1.096902
C	4.239407	-0.762680	0.904519
C	4.413690	-1.608344	-0.143534
N	3.151701	-1.972082	-0.594783
C	2.246957	0.116371	2.188477
C	2.861162	-2.866666	-1.698985
O	-1.368007	-0.781495	1.199291
N	0.214283	0.986697	-0.135160
C	-0.289692	2.201544	-0.157874
N	0.477907	3.296618	-0.475188
C	-0.294642	4.447444	-0.429387
C	-1.553443	4.071492	-0.090277
N	-1.549946	2.690457	0.084479

C	1.895035	3.234426	-0.778850
C	-2.735816	1.913878	0.420436
H	-0.698971	-1.024401	-2.487186
H	0.119674	5.429350	-0.637778
H	-2.454971	4.660311	0.049241
H	4.966078	-0.264195	1.538961
H	5.321575	-1.986262	-0.604083
H	2.916338	0.097551	3.058627
H	2.031362	1.155535	1.903926
H	1.301242	-0.374370	2.457667
H	3.427176	-3.802463	-1.586558
H	1.786920	-3.087055	-1.676270
H	3.120871	-2.400340	-2.662077
H	2.491655	3.657107	0.044673
H	2.162971	2.179965	-0.915236
H	2.107914	3.793380	-1.701266
H	-3.388161	2.527539	1.055450
H	-3.289116	1.633412	-0.490145
H	-2.445454	1.006732	0.965585
O	-3.499777	-1.592118	-0.675250
C	-3.604540	-2.792079	0.259345
H	-3.302917	-3.659086	-0.343860
H	-2.857341	-2.562347	1.031557
C	-5.019365	-2.848697	0.754025
H	-5.734764	-3.003577	-0.066620
H	-5.104511	-3.698553	1.449383
H	-5.283726	-1.933348	1.302681

First transition state (TS1e)

Electronic energy = -1366.04369146

Thermal correction to enthalpy = 0.391674

Entropy correction = 0.076722

Lowest frequency = -310.18 cm^{-1}

Number of atoms: 45

charge=+1, multiplicity=1

C	-2.400485	-1.297654	-1.059946
C	-1.321319	-0.897180	-1.637718
P	-0.279039	-0.488797	-0.121600
N	0.982884	-1.473901	-0.273003
C	2.230084	-1.328349	0.109963
N	2.735521	-0.739894	1.242720
C	4.119674	-0.887167	1.264769
C	4.474270	-1.575612	0.150129
N	3.309361	-1.844695	-0.557698
C	1.935555	-0.143722	2.298408
C	3.218148	-2.565197	-1.812942
O	-1.346489	-0.893354	0.909328
N	0.211240	1.044517	-0.197616
C	-0.305821	2.251099	-0.142237
N	0.407590	3.366586	-0.513419
C	-0.364504	4.504830	-0.339328
C	-1.569220	4.103136	0.138842
N	-1.531670	2.717764	0.266925
C	1.773641	3.332954	-1.000799
C	-2.647563	1.920073	0.754539
H	-1.072136	-0.907935	-2.700059
H	0.011448	5.497607	-0.568145
H	-2.451822	4.675985	0.406923
H	4.724892	-0.495894	2.076903
H	5.449302	-1.899829	-0.201019
H	2.453416	-0.277293	3.257354
H	1.763096	0.925940	2.113464
H	0.960796	-0.649421	2.347164
H	3.666379	-3.564897	-1.714408
H	2.154940	-2.667634	-2.061244
H	3.732026	-2.015516	-2.616295
H	2.448064	3.849170	-0.300620
H	2.070843	2.280939	-1.084226
H	1.839388	3.815030	-1.987293
H	-3.146917	2.466039	1.566418
H	-3.376886	1.737734	-0.051150
H	-2.279372	0.957230	1.130836
O	-3.497636	-1.656789	-0.629039
C	-3.578847	-2.924869	0.199541
H	-3.211972	-3.727402	-0.455180
H	-2.865123	-2.729649	1.011847
C	-5.004920	-3.089849	0.635926
H	-5.682860	-3.212296	-0.221136
H	-5.070095	-3.996768	1.257225
H	-5.335444	-2.235644	1.244078

Closed form (CFe)

Electronic energy = -1366.09975869

Thermal correction to enthalpy = 0.395002

Entropy correction = 0.076252

Lowest frequency = 22.18 cm^{-1}

Number of atoms: 45

charge=+1, multiplicity=1

C	-1.767217	-1.568619	-0.749101
C	-1.224495	-0.767824	-1.716817
P	0.003599	-0.295943	-0.553077
N	1.450015	-0.921211	-0.733149
C	2.566984	-0.823048	-0.035568
N	2.750349	-0.740858	1.316550

C	4.112987	-0.728721	1.597854
C	4.772495	-0.811578	0.413573
N	3.813917	-0.870860	-0.589687
C	1.674252	-0.730192	2.294663
C	4.071056	-0.979379	-2.015578
O	-1.022329	-1.420824	0.385197
N	0.025090	1.158467	0.115804
C	-0.837192	2.165767	0.097022
N	-0.495941	3.447979	-0.230707
C	-1.597627	4.278614	-0.087470
C	-2.631569	3.505847	0.334894
N	-2.156142	2.204715	0.454124
C	0.826249	3.855133	-0.674286
C	-2.937612	1.073888	0.927929
H	-1.452080	-0.612795	-2.765240
H	-1.548456	5.343018	-0.297175
H	-3.660649	3.763611	0.566541
H	4.488156	-0.668303	2.614961
H	5.835962	-0.836561	0.195636
H	2.097107	-0.934710	3.285684
H	1.163655	0.242984	2.299546
H	0.942290	-1.512777	2.049986
H	4.711452	-1.849665	-2.218723
H	3.107583	-1.110007	-2.522372
H	4.563736	-0.069640	-2.390256
H	1.194659	4.683035	-0.051852
H	1.494842	2.992510	-0.571559
H	0.799006	4.174882	-1.726727
H	-3.635598	1.419201	1.701876
H	-3.503388	0.613669	0.104405
H	-2.261922	0.324889	1.356062
O	-2.808468	-2.349137	-0.754682
C	-3.084228	-3.179092	0.408009
H	-2.197452	-3.799681	0.606154
H	-3.240779	-2.520197	1.275723
C	-4.305446	-4.010155	0.095303
H	-4.129084	-4.653721	-0.779028
H	-4.543387	-4.652215	0.956651
H	-5.176099	-3.370154	-0.111380

Second transition state (TS2e)

Electronic energy = -1366.08314128

Thermal correction to enthalpy = 0.393695

Entropy correction = 0.075644

Lowest frequency = -139.53 cm^{-1}

Number of atoms: 45

charge=+1, multiplicity=1

C	-1.904945	-1.542577	-0.440311
C	-1.429678	-0.585122	-1.380797
P	0.052392	-0.095638	-0.724362
N	1.317523	-1.036729	-0.761698
C	2.424694	-1.077312	-0.027272
N	2.570358	-0.944896	1.320582
C	3.903120	-1.151078	1.656206
C	4.576075	-1.417991	0.505597
N	3.652628	-1.377982	-0.529868
C	1.479998	-0.691268	2.253226
C	3.925425	-1.610872	-1.939849
O	-1.290971	-1.670975	0.649916
N	0.371625	1.384758	-0.223401
C	-0.471210	2.403849	-0.020787
N	-0.189663	3.679715	-0.404653
C	-1.213004	4.522022	-0.000477
C	-2.128073	3.756341	0.650623
N	-1.663211	2.447428	0.639104
C	0.995723	4.068189	-1.153820
C	-2.357897	1.327935	1.262343
H	-1.899740	-0.231804	-2.295667
H	-1.198661	5.587990	-0.207674
H	-3.066285	4.024493	1.127195
H	4.249326	-1.101308	2.684185
H	5.625141	-1.638899	0.332848
H	1.779439	-1.043864	3.247753
H	1.253811	0.384363	2.296315
H	0.585520	-1.240381	1.928501
H	4.587839	-2.480350	-2.046554
H	2.972339	-1.811513	-2.444145
H	4.401666	-0.729903	-2.396150
H	1.395248	5.006510	-0.746525
H	1.741664	3.271375	-1.049943
H	0.755047	4.204823	-2.218919
H	-2.777417	1.661665	2.220806
H	-3.162055	0.964102	0.607726
H	-1.665123	0.497545	1.439229
O	-2.999310	-2.230305	-0.728397
C	-3.485665	-3.163212	0.262264
H	-2.715858	-3.933471	0.424747
H	-3.619967	-2.629109	1.216254
C	-4.779364	-3.750668	-0.253931
H	-4.619795	-4.273504	-1.208774
H	-5.177092	-4.473646	0.474250
H	-5.534699	-2.965770	-0.410738

Open form (OFe)

Electronic energy = -1366.10730780

Thermal correction to enthalpy = 0.395053

Entropy correction = 0.078164

Lowest frequency = 16.11 cm^{-1}

Number of atoms: 45

charge=+1, multiplicity=1

C	-1.252682	1.606889	-0.147903
C	0.096364	1.075927	-0.301190
P	0.460775	-0.526367	-0.010488
N	-0.567161	-1.673959	0.308102
C	-1.890684	-1.743656	0.139784
N	-2.789547	-1.930167	1.134056
C	-4.061761	-2.025125	0.588241
C	-3.929614	-1.925657	-0.760761
N	-2.576599	-1.769657	-1.030178
C	-2.456665	-1.845354	2.544653
C	-1.972216	-1.536332	-2.327546
O	-2.241110	0.951652	0.152108
N	1.956389	-1.049251	-0.045528
C	3.113347	-0.408724	0.062677
N	4.197233	-0.653737	-0.724480
C	5.267656	0.117797	-0.293058
C	4.841320	0.839439	0.776025
N	3.508047	0.508214	0.995447
C	4.203381	-1.580852	-1.845456
C	2.683957	0.971821	2.100736
H	0.913567	1.740703	-0.585092
H	6.236005	0.084029	-0.783407
H	5.365824	1.553398	1.403940
H	-4.943796	-2.164589	1.205605
H	-4.672823	-1.964849	-1.551118
H	-3.191002	-2.416556	3.126297
H	-1.456560	-2.269353	2.699738
H	-2.462250	-0.793623	2.870071
H	-1.757001	-0.465673	-2.464354
H	-1.038943	-2.110915	-2.407024
H	-2.662905	-1.870990	-3.111038
H	5.095000	-2.220258	-1.791110
H	3.302683	-2.202512	-1.777062
H	4.202144	-1.035261	-2.801089
H	3.334679	1.419216	2.861747
H	1.945309	1.714172	1.765850
H	2.154910	0.117347	2.546790
O	-1.290457	2.926183	-0.372732
C	-2.572836	3.574644	-0.251365
H	-3.279899	3.091441	-0.944434
H	-2.956935	3.410073	0.767975
C	-2.381529	5.042376	-0.560677
H	-1.997580	5.183120	-1.582356
H	-3.343598	5.570162	-0.477484
H	-1.672699	5.504503	0.142972

Reaction of $[(R^{Me})_2PO]^+$ with (dimethylamino)acetylene

Reactant complex (RCf)

Electronic energy = -1346.22612773

Thermal correction to enthalpy = 0.405989

Entropy correction = 0.078766

Lowest frequency = 16.42 cm^{-1}

Number of atoms: 46

charge=+1, multiplicity=1

C	1.295374	2.684175	-0.990772
C	0.663039	1.669647	-1.456826
P	0.027890	0.604416	0.009617
N	-1.559130	0.808783	-0.231748
C	-2.582515	0.058848	0.095184
N	-2.742473	-0.826334	1.134790
C	-4.032202	-1.352437	1.107107
C	-4.680423	-0.786755	0.058672
N	-3.787389	0.078784	-0.562196
C	-1.738596	-1.115297	2.142399
C	-4.061296	0.895708	-1.727064
O	0.666056	1.251608	1.221255
N	0.442070	-0.905181	-0.412977
C	1.484560	-1.688109	-0.252984
N	1.883746	-2.596182	-1.206259
C	2.950785	-3.345944	-0.733810
C	3.224231	-2.906187	0.520775
N	2.328017	-1.883562	0.815500
C	1.259079	-2.727750	-2.508268
C	2.218119	-1.252505	2.124364
H	0.457904	1.417326	-2.497534
H	3.422786	-4.119572	-1.332183
H	3.977449	-3.226486	1.234401
H	-4.371288	-2.076553	1.841609
H	-5.696200	-0.920829	-0.300802
H	-2.241470	-1.401481	3.075577
H	-1.072579	-1.929001	1.819673
H	-1.133367	-0.216351	2.325332
H	-4.884982	1.596860	-1.524058
H	-3.151252	1.463493	-1.955044
H	-4.327647	0.265649	-2.589383
H	0.338856	-2.130965	-2.495289
H	1.924834	-2.358980	-3.304257
H	1.012612	-3.781169	-2.704670
H	1.625488	-1.883848	2.806130
H	3.225776	-1.131673	2.544935
H	1.743967	-0.267336	2.021326

C	1.040542	4.666669	0.306565
H	1.596836	5.610781	0.229421
H	0.087727	4.740730	-0.229066
H	0.850560	4.416572	1.361210
N	1.843688	3.575509	-0.275888
C	3.199389	3.382538	0.267314
H	3.753128	4.329472	0.210125
H	3.098274	3.058237	1.313930
H	3.715573	2.607699	-0.310160

First transition state (TS1f)

Electronic energy = -1346.21593509

Thermal correction to enthalpy = 0.404966

Entropy correction = 0.077535

Lowest frequency = -309.66 cm^{-1}

Number of atoms: 46

charge=+1, multiplicity=1

C	1.386411	2.374485	-0.726567
C	0.765706	1.552086	-1.533231
P	-0.052676	0.517501	-0.262046
N	-1.631063	0.702465	-0.496235
C	-2.679634	0.117186	0.031215
N	-2.839141	-0.491516	1.251571
C	-4.155166	-0.928313	1.386828
C	-4.817219	-0.580577	0.255183
N	-3.906938	0.060760	-0.576713
C	-1.798583	-0.619541	2.255526
C	-4.187125	0.604668	-1.891199
O	0.617079	1.362770	0.876615
N	0.352619	-1.038890	-0.357996
C	1.445677	-1.746996	-0.171786
N	1.871668	-2.719825	-1.041502
C	2.988769	-3.362067	-0.526438
C	3.264533	-2.786826	0.671829
N	2.319672	-1.788254	0.886619
C	1.226345	-3.010901	-2.308070
C	2.188914	-1.033232	2.125222
H	0.781192	1.576690	-2.625208
H	3.490166	-4.164463	-1.059433
H	4.049382	-2.994949	1.392884
H	-4.499353	-1.442233	2.279120
H	-5.853215	-0.730887	-0.033050
H	-2.267946	-0.744772	3.239746
H	-1.149077	-1.481581	2.044397
H	-1.181586	0.290229	2.266905
H	-4.932953	1.411400	-1.826413
H	-3.249666	1.010149	-2.290128
H	-4.562649	-0.182747	-2.561727
H	0.280002	-2.456838	-2.332730
H	1.858420	-2.695848	-3.152693
H	1.024844	-4.088589	-2.390192
H	1.618576	-1.611325	2.870206
H	3.191574	-0.834308	2.527272
H	1.683302	-0.080261	1.923035
C	1.404623	4.602789	0.109135
H	2.052493	5.465002	-0.101315
H	0.462560	4.703949	-0.442494
H	1.190721	4.549465	1.188621
N	2.089604	3.373528	-0.317206
C	3.430259	3.136273	0.231720
H	4.067022	4.004837	0.013526
H	3.362914	2.990402	1.322037
H	3.860766	2.239925	-0.230558

Closed form (CFf)

Electronic energy = -1346.25647286

Thermal correction to enthalpy = 0.407349

Entropy correction = 0.078022

Lowest frequency = 19.05 cm^{-1}

Number of atoms: 46

charge=+1, multiplicity=1

C	-0.258767	1.779449	-0.468141
C	0.043033	0.777940	-1.371673
P	0.061587	-0.366532	-0.031808
N	-1.124714	-1.392835	0.236055
C	-2.430938	-1.191320	0.191782
N	-3.216203	-0.677954	1.183862
C	-4.548542	-0.730549	0.796278
C	-4.587824	-1.288029	-0.443117
N	-3.278272	-1.570965	-0.809055
C	-2.698984	-0.191907	2.452173
C	-2.843159	-2.168116	-2.058797
O	-0.314898	1.183239	0.766803
N	1.411894	-1.038877	0.459059
C	2.693172	-0.807131	0.255402
N	3.594560	-1.784889	-0.063976
C	4.870006	-1.243371	-0.133971
C	4.762173	0.080701	0.147470
N	3.418256	0.345532	0.392856
C	3.237907	-3.175928	-0.285142
C	2.877551	1.635967	0.782410
H	0.212164	0.759126	-2.442022
H	5.740162	-1.847407	-0.373210
H	5.518946	0.857329	0.203018

H	-5.349858	-0.377121	1.438269
H	-5.429315	-1.513505	-1.091337
H	-3.466875	-0.317014	3.226053
H	-1.811663	-0.783110	2.715038
H	-2.404435	0.864242	2.378737
H	-2.869225	-1.431649	-2.876205
H	-1.813951	-2.522676	-1.923654
H	-3.492299	-3.017638	-2.311798
H	2.190841	-3.304664	0.015171
H	3.351068	-3.442476	-1.346954
H	3.878754	-3.828881	0.324074
H	3.609885	2.158377	1.412168
H	2.649985	2.251625	-0.100595
H	1.954773	1.482073	1.353648
C	-0.406548	3.750243	-1.855512
H	0.578687	4.222457	-2.016228
H	-0.587781	3.024941	-2.658884
H	-1.178789	4.531666	-1.916906
N	-0.481719	3.094512	-0.561210
C	-0.672846	3.934506	0.611451
H	0.145864	4.669123	0.701040
H	-1.623009	4.487892	0.533693
H	-0.692571	3.312968	1.512755

Second transition state (TS2f)

Electronic energy = -1346.23914410

Thermal correction to enthalpy = 0.406032

Entropy correction = 0.077029

Lowest frequency = -145.84 cm^{-1}

Number of atoms: 46

charge=+1, multiplicity=1

C	-0.758659	1.652038	-0.210849
C	0.013528	0.809051	-1.079238
P	0.275925	-0.582242	-0.124884
N	-0.806205	-1.661915	0.287855
C	-2.127296	-1.480485	0.209633
N	-2.952746	-1.237071	1.257318
C	-4.260661	-1.175036	0.805754
C	-4.237559	-1.401013	-0.536613
N	-2.910908	-1.590939	-0.896470
C	-2.483434	-0.970735	2.609284
C	-2.400785	-1.856182	-2.232158
O	-0.921743	1.198396	0.972999
N	1.727492	-1.160910	0.141549
C	2.903912	-0.543349	0.152466
N	4.053766	-1.140066	-0.270265
C	5.124425	-0.283082	-0.065790
C	4.630825	0.849128	0.499659
N	3.256486	0.684779	0.636484
C	4.114931	-2.473489	-0.848706
C	2.368505	1.669397	1.238412
H	0.557908	1.052025	-1.989616
H	6.140537	-0.556386	-0.334262
H	5.132534	1.755187	0.825830
H	-5.093136	-0.986401	1.476924
H	-5.045151	-1.457847	-1.260187
H	-3.274386	-1.234933	3.322070
H	-1.594244	-1.586385	2.795202
H	-2.208811	0.089099	2.699585
H	-2.030177	-0.930190	-2.695398
H	-1.574877	-2.576476	-2.162932
H	-3.204112	-2.284432	-2.844320
H	3.197291	-3.007199	-0.572664
H	4.190218	-2.420815	-1.945490
H	4.987677	-3.006402	-0.448271
H	2.886904	2.144639	2.081869
H	2.078518	2.432438	0.502188
H	1.452937	1.190609	1.606801
C	-1.184011	3.344783	-1.942020
H	-0.355491	4.066149	-2.061398
H	-1.038187	2.516416	-2.646426
H	-2.120182	3.858190	-2.211350
N	-1.278068	2.844454	-0.583244
C	-1.911913	3.710925	0.397399
H	-1.436232	4.706182	0.393962
H	-2.983841	3.842323	0.170586
H	-1.810187	3.260648	1.390589

Open form (OFf)

Electronic energy = -1346.25277216

Thermal correction to enthalpy = 0.407370

Entropy correction = 0.078909

Lowest frequency = 21.10 cm^{-1}

Number of atoms: 46

charge=+1, multiplicity=1

C	-1.202600	1.772746	-0.166755
C	0.110809	1.135878	-0.341870
P	0.332877	-0.488191	-0.023094
N	-0.767944	-1.565957	0.299639
C	-2.096239	-1.515744	0.157069
N	-2.994102	-1.518719	1.168052
C	-4.276034	-1.533105	0.639399
C	-4.154744	-1.577741	-0.713787
N	-2.796170	-1.584068	-1.002200

C	-2.630111	-1.270532	2.550741
C	-2.184866	-1.545495	-2.316688
O	-2.169020	1.129150	0.269283
N	1.786504	-1.127833	-0.067382
C	2.989257	-0.598664	0.092198
N	4.075406	-0.929476	-0.662163
C	5.196374	-0.267634	-0.178831
C	4.800371	0.473577	0.888120
N	3.434494	0.264631	1.055164
C	4.037645	-1.839217	-1.796078
C	2.618573	0.777287	2.143664
H	1.007626	1.674045	-0.646769
H	6.174711	-0.383995	-0.635537
H	5.365899	1.124988	1.547616
H	-5.158774	-1.521153	1.271197
H	-4.909147	-1.622825	-1.493108
H	-3.443102	-1.607294	3.205678
H	-1.714592	-1.827988	2.786880
H	-2.452478	-0.193401	2.691144
H	-1.881476	-0.518103	-2.571118
H	-1.302068	-2.199345	-2.328115
H	-2.906146	-1.905448	-3.060547
H	3.071313	-2.357343	-1.782212
H	4.145572	-1.287107	-2.741880
H	4.848865	-2.575048	-1.708548
H	3.279721	1.171681	2.924774
H	1.941902	1.571648	1.797119
H	2.017460	-0.039570	2.569113
C	-0.200143	3.883800	-1.005427
H	0.545164	4.121485	-0.224653
H	0.310508	3.375997	-1.837506
H	-0.589186	4.834293	-1.393517
N	-1.309644	3.096126	-0.496578
C	-2.562735	3.790050	-0.239116
H	-2.424818	4.587265	0.511694
H	-2.949821	4.248412	-1.164234
H	-3.291294	3.064316	0.137122

4 References

(1) Löwe, P.; Witteler, T.; Dielmann, F. Lewis base-free thiophosphonium ion: a cationic sulfur atom transfer reagent. *Chem. Commun.* **2021**, *57* (41), 5043–5046.

(2) *APEX2 Version 2.1 - 0*; Bruker AXS Inc. Madison, 2004.

(3) Dolomanov, O. V.; Bourhis, L. J.; Gildea, R. J.; Howard, J. A. K.; Puschmann, H. OLEX2 : a complete structure solution, refinement and analysis program. *J. Appl. Crystallogr.* **2009**, *42* (2), 339–341.

(4) Sheldrick, G. M. A short history of SHELX. *Acta crystallographica. Section A, Foundations of crystallography* **2008**, *64* (Pt 1), 112–122.

(5) Lee; Yang; Parr. Development of the Colle-Salvetti correlation-energy formula into a functional of the electron density. *Physical review. B, Condensed matter* **1988**, *37* (2), 785–789.

(6) Becke, A. D. Density-functional thermochemistry. III. The role of exact exchange. *J. Chem. Phys.* **1993**, *98* (7), 5648–5652.

(7) Grimme, S.; Ehrlich, S.; Goerigk, L. Effect of the damping function in dispersion corrected density functional theory. *J. Comput. Chem.* **2011**, *32* (7), 1456–1465.

(8) Weigend, F.; Ahlrichs, R. Balanced basis sets of split valence, triple zeta valence and quadruple zeta valence quality for H to Rn: Design and assessment of accuracy. *Phys. Chem. Chem. Phys* **2005**, *7* (18), 3297–3305.

(9) Eichkorn, K.; Weigend, F.; Treutler, O.; Ahlrichs, R. Auxiliary basis sets for main row atoms and transition metals and their use to approximate Coulomb potentials. *Theor. Chem. Acc.* **1997** (97), 119–124.

(10) Hansen, A.; Liakos, D. G.; Neese, F. Efficient and accurate local single reference correlation methods for high-spin open-shell molecules using pair natural orbitals. *J. Chem. Phys.* **2011**, *135* (21), 214102.

(11) Riplinger, C.; Neese, F. An efficient and near linear scaling pair natural orbital based local coupled cluster method. *J. Chem. Phys.* **2013**, *138* (3), 34106.

(12) Riplinger, C.; Sandhoefer, B.; Hansen, A.; Neese, F. Natural triple excitations in local coupled cluster calculations with pair natural orbitals. *J. Chem. Phys.* **2013**, *139* (13), 134101.

(13) Riplinger, C.; Pinski, P.; Becker, U.; Valeev, E. F.; Neese, F. Sparse maps—A systematic infrastructure for reduced-scaling electronic structure methods. II. Linear scaling domain based pair natural orbital coupled cluster theory. *J. Chem. Phys.* **2016**, *144* (2), 24109.

(14) Saitow, M.; Becker, U.; Riplinger, C.; Valeev, E. F.; Neese, F. A new near-linear scaling, efficient and accurate, open-shell domain-based local pair natural orbital coupled cluster singles and doubles theory. *J. Chem. Phys.* **2017**, *146* (16), 164105.

(15) Saitow, M.; Neese, F. Accurate spin-densities based on the domain-based local pair-natural orbital coupled-cluster theory. *J. Chem. Phys.* **2018**, *149* (3), 34104.

(16) Ribeiro, R. F.; Marenich, A. V.; Cramer, C. J.; Truhlar, D. G. Use of solution-phase vibrational frequencies in continuum models for the free energy of solvation. *J. Phys. Chem. B* **2011**, *115* (49), 14556–14562.

(17) Funes-Ardois, I.; Paton, R. *GoodVibes v1.*, 2016.

(18) Marenich, A. V.; Cramer, C. J.; Truhlar, D. G. Universal solvation model based on solute electron density and on a continuum model of the solvent defined by the bulk dielectric constant and atomic surface tensions. *J. Phys. Chem. B* **2009**, *113* (18), 6378–6396.

(19) Reed, A. E.; Weinstock, R. B.; Weinhold, F. Natural population analysis. *J. Chem. Phys.* **1985**, *83* (2), 735–746.

(20) Glendening, E. D.; Carpenter, J. E.; Reed, A. E.; Weinhold, F. *NBO Version 3.1*.

(21) Gaussian Inc.: Wallingford CT. *Gaussian 09*; Frisch, M. J.; Trucks, G. W.; Schlegel, H. B.; Scuseria, G. E.; Robb, M. A.; Cheeseman, J. R.; Scalmani, G.; Barone, V.; Mennucci, B.; Petersson, G. A.; Nakatsuji, H.; Caricato, M.; Hratchian, H. P.; Izmaylov, A. F.; Bloino, J.; Zheng, G.; Sonnenberg, J. L.; Hada, M.; Ehara, M.; Toyota, K.; Fukuda, R.; Hasegawa, J.; Ishida, M.; Nakajima, T.; Honda, Y.; Kitao, O.; Nakai, H.; Vreven, T.; Montgomery, J. A., Jr.; Peralta, J. E.; Ogliaro, F.; Bearpark, M. J.; Heyd, J. J.; Brothers, E. N.; Kudin, K. N.; Staroverov, V. N.; Kobayashi, R.; Normand, J.; Raghavachari, K.; Rendell, A. P.; Burant, J. C.; Iyengar, S. S.; Tomasi, J.; Cossi, M.; Rega, N.; Millam, J. M.; Klene, M.; Knox, J. E.; Cross, J. B.; Bakken, V.; Adamo, C.; Jaramillo, J.; Gomperts, R.; Stratmann, R. E.; Yazyev, A. J.; Austin, A. J.; Cammi, R.; Pomelli, J. W.; Ochterski, J. W.; Martin, R. L.; Morokuma, K.; Zakrzewski, V. G.; Voth, G. A.; Salvador, P.; Dannenberg, J. J.; Dapprich, S.; Daniels, A. D.; Farkas, Ö.; Foresman, J. B.; Ortiz, J. V.; Cioslowski, J.; Fox, D. J., 2009.

(22) Neese, F. Software update: the ORCA program system, version 4.0. *WIREs Comput. Mol. Sci.* **2018**, *8* (1), e1327.

(23) Löwe, P.; Feldt, M.; Wünsche, M. A.; Wilm, L. F. B.; Dielmann, F. Oxophosphonium-Alkyne Cycloaddition Reactions: Reversible Formation of 1,2-Oxaphosphetes and Six-membered Phosphorus Heterocycles. *J. Am. Chem. Soc.* **2020**, *142* (21), 9818–9826.
